# Incandescent temporal metamaterials

**DOI:** 10.1038/s41467-023-40281-2

**Published:** 2023-08-01

**Authors:** J. Enrique Vázquez-Lozano, Iñigo Liberal

**Affiliations:** Department of Electrical, Electronic and Communications Engineering, Institute of Smart Cities (ISC), Universidad Pública de Navarra (UPNA), 31006 Pamplona, Spain

**Keywords:** Nanophotonics and plasmonics, Metamaterials

## Abstract

Regarded as a promising alternative to spatially shaping matter, time-varying media can be seized to control and manipulate wave phenomena, including thermal radiation. Here, based upon the framework of macroscopic quantum electrodynamics, we elaborate a comprehensive quantum theoretical formulation that lies the basis for investigating thermal emission effects in time-modulated media. Our theory unveils unique physical features brought about by time-varying media: nontrivial correlations between fluctuating electromagnetic currents at different frequencies and positions, thermal radiation overcoming the black-body spectrum, and quantum vacuum amplification effects at finite temperature. We illustrate how these features lead to striking phenomena and innovative thermal emitters, specifically, showing that the time-modulation releases strong field fluctuations confined within epsilon-near-zero (ENZ) bodies, and that, in turn, it enables a narrowband (partially coherent) emission spanning the whole range of wavevectors, from near to far-field regimes.

## Abstract (web summary)

Here the authors employ quantum electrodynamics to formulate thermal emission effects in time-modulated media, resulting in innovative thermal emitters. They show that time modulation induces strong field fluctuations in epsilon-near-zero bodies, enabling narrowband emission across the entire range of wavevectors, from near to far-field regimes.

## Introduction

On the basis of the latest scientific and technological breakthroughs in nanophotonics and material science, the development of metamaterials have brought forth an ideal playground for engineering innovative forms of light-matter interactions^1^. In the quest for reaching an increasing control over wave phenomena, a recent burgeoning approach consists in harnessing time as an additional degree of freedom to be exploited^2^. This revival of time-varying media^3^, has in turn boosted the discovery of new physics and applications associated with time-dependent optical phenomena^4^, ultimately giving rise to the emerging field of temporal metamaterials^5,6^. The realization of this class of time-dependent materials is concomitantly tied to the prevalence of a temporal modulation over the constitutive parameters characterizing the response of matter, which, in general, should be externally driven.

Another research area where structuring matter and shaping their optical properties is attracting a great deal of attention is the engineering of thermal emission^7–9^. As a basic mechanism of heat transfer^10^, whereby an incandescent object at finite temperature emits (thermal) light^11^, thermal radiation is of fundamental interest. Likewise, it is also the basis of multiple technological applications including heat and energy management^12,13^, light sources^14^, sensing^15^, and communications^16^. In sharp contrast to the behavior of (non-thermal) light emanating from coherent sources, such as lasers or antennas, thermal light is characterized for displaying a broadband spectrum as well as an almost isotropic and unpolarized field distribution. Due to these properties, ultimately abridged into its inherently incoherent nature, the control and manipulation of thermal fields has long been (and continues to be) a challenging issue. Significant efforts have been made to explore and stretch out the physical limits of thermal radiation (imposed by Planck’s^17–19^ and Kirchhoff’s radiation laws^20,21^), mainly by looking into the distinctive features occurring at the nanoscale^22–25^. Practical implementations have mostly been based on metamaterials^26^, metasurfaces^27^, photonic crystals^28^, or subwavelength structures such as gratings^29^, to name a few. In this sense, besides affording a better far-field thermal emission performance (e.g., via the so-called thermal extraction schemes^30^), nanophotonic engineering has stimulated the unveiling of a plethora of near-field thermal effects^31^. Akin to customary coherent optical sources, the near-field radiation of thermal emitters greatly differs from that in the far-field regime^32–34^. This is essentially due to the existence of evanescent modes, which are dominant in the near-field and negligible in the far-field^35^. Apart from modifying the spectral distribution, the evanescent contribution gives access to additional channels over the frequency-wavevector (*ω*-*k*) space, thus strengthening thermal fields by several orders of magnitude^29,32–34^.

**Fig. 1:**
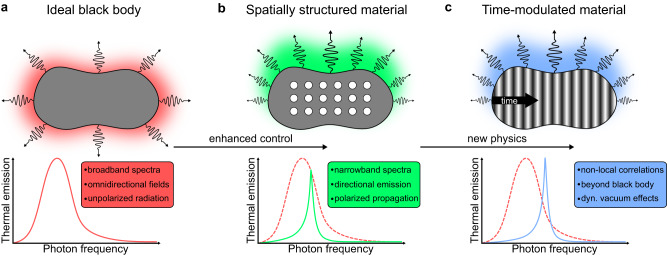
Breakthroughs in thermal emission engineering. **a** According to Planck’s law, the broadband emission spectrum (tied to isotropic and unpolarized thermal radiation) of a black body in thermal equilibrium only depends on the temperature. **b** Thermal emission can be controlled by structuring the matter, enabling narrowband, directive, and polarized radiation. **c** Modulating temporally the optical properties of a medium enables more sophisticated ways to obtain similar effects, yielding the emergence of new physics, such as non-local correlations, overcoming the black-body spectrum, or dynamical vacuum effects.

Just like in the field of nanophotonics, it seems natural to think that passing from spatially structured to time-modulated materials could revolutionize the field of thermal engineering [see Fig. 1]. A clear example comes through the grating structures^29^, whose temporal analog could similarly open new opportunities^36^. Moreover, the temporal dimension owns in itself some fundamental attributes tied to the principle of causality^37^. Nonetheless, the topic of thermal emission in time-varying media is at a very incipient stage^38–40^ and the underlying physics is not fully understood as yet.

Upon this ground, here we put forward a quantum formalism to address near- and far-field thermal emission in time-varying media. Noteworthily, this theoretical formulation would also be extensible to purely quantum phenomena (e.g., Casimir forces^41^ and dynamical vacuum amplification effects^42–44^) at finite temperature. Our formalism allows us to unveil some distinctive thermal properties associated with time-modulated materials, including fluctuating currents with non-local (space and frequency) correlations, and far-field thermal emission beyond the black-body spectrum. In turn, these properties lead to unprecedented wave phenomena empowered by the time-modulation, such as the releasing of fluctuations trapped within a material body, or the narrowband near-to-far field thermal linking.

## Results

### Semiclassical approach to thermal radiation from fluctuating currents

**Fig. 2:**
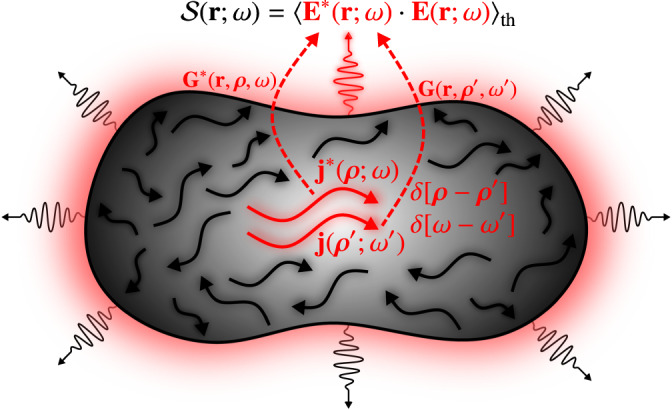
Thermal emission from EM fluctuations. Schematic representation of the fluctuating density currents moving inside a hot body. The solid red arrows represent two particular electric currents which are locally correlated, releasing so, via the corresponding Green’s function (dashed red arrows), the emission of thermal fields out the material.

Theoretical modeling of thermal emission is typically carried out within the framework of fluctuational electrodynamics^45^. According to this semiclassical approach, the incandescence, i.e., the emission of thermal radiation emanating from a hot body at temperature *T*, can be simply understood as a result of the radiation emitted by the fluctuating electromagnetic (EM) currents (mathematically characterized by means of the current density correlations) [see Fig. 2]. The theoretical cornerstone of this formalism is the fluctuation-dissipation theorem (FDT)^46–48^, which, in this context, provides with a relationship between the correlations of the fluctuating EM currents and the dissipative features associated to the response function:

1 $${\langle {{{{{{{{\bf{j}}}}}}}}}^{*}({{{{{{{\boldsymbol{\rho }}}}}}}};\omega )\cdot {{{{{{{\bf{j}}}}}}}}({{{{{{{{\boldsymbol{\rho }}}}}}}}}^{{\prime} };{\omega }^{{\prime} })\rangle }_{{{{{{{{\rm{th}}}}}}}}}=4\pi {\varepsilon }_{0}{\varepsilon }^{{\prime\prime} }({{{{{{{\boldsymbol{\rho }}}}}}}},\, \, \omega )\hslash {\omega }^{2}\Theta (\omega,\, \, T)\delta [\omega -{\omega }^{{\prime} }]\delta [{{{{{{{\boldsymbol{\rho }}}}}}}}-{{{{{{{{\boldsymbol{\rho }}}}}}}}}^{{\prime} }],\,$$

where the brackets 〈⋯〉_th_ denote a thermal ensemble average, $\Theta (\omega,\, T)={[{e}^{\hslash \omega /({k}_{B}T)}-1]}^{-1}$, and $\varepsilon ({{{{{{{\boldsymbol{\rho }}}}}}}},\, \, \omega )={\varepsilon }^{{\prime} }({{{{{{{\boldsymbol{\rho }}}}}}}},\, \, \omega )+i{\varepsilon }^{{\prime\prime} }({{{{{{{\boldsymbol{\rho }}}}}}}},\, \, \omega )$ is the lossy and dispersive permittivity of the body (i.e., the linear response function). A very important feature of Eq. (1) is that thermally fluctuating currents at different frequencies and positions are uncorrelated, describing the stochastic nature of thermal fields.

Once the fluctuating currents are known, the spectral energy density, ${{{{{{{\mathcal{S}}}}}}}}({{{{{{{\bf{r}}}}}}}};\omega )={\langle {{{{{{{{\bf{E}}}}}}}}}^{*}({{{{{{{\bf{r}}}}}}}};\omega )\cdot {{{{{{{\bf{E}}}}}}}}({{{{{{{\bf{r}}}}}}}};\omega )\rangle }_{{{{{{{{\rm{th}}}}}}}}}$, at a given position, **r**, and frequency, *ω*, can be directly found from the connection between fields and currents **E**(**r**; *ω*) = *iωμ*_0_ ∫ *d*^3^ ***ρ*** **G**(**r**, ***ρ***, *ω*) ⋅ **j**(***ρ***; *ω*) via the dyadic Green’s function of the body, **G**(**r**, ***ρ***, *ω*) [see Fig. 2], where, for convenience in the succeeding developments, ***ρ*** will henceforth stands for the spatial variables associated to the radiation sources, i.e., the fluctuating EM currents.

Fluctuational electrodynamics has proven to be a very successful theory that has made possible groundbreaking advances in engineering thermal fields with photonic nanostructures. However, as a semiclassical theory, it does not allow for the simultaneous modeling of quantum vacuum and thermal fluctuations, particularly in their interaction with dynamical systems. In addition, its extension and applicability to time-varying media is not rigorously justified^38,39^. In the following, from a first-principles approach, we introduce a full-quantum formalism to thermal emission in time-varying media, which enables the calculation of quantum and thermally fluctuating current correlations, and thermal fields, without the need of any additional assumptions beyond those implicitly set in the Hamiltonian of the system.

### Quantum approach to thermal emission in time-varying media

To establish a general theoretical framework for extending the study of thermal emission and the behavior of thermal (and zero-point quantum vacuum) fluctuating currents and fields in time-varying media, we make use of macroscopic quantum electrodynamics^49,50^. Within this framework, instead of bare photons, one actually deals with elementary excitations, namely, EM field-matter coupled states modeled by a continuum of harmonic oscillators^51^. These modes are described by polaritonic operators, $\hat{{{{{{{{\bf{f}}}}}}}}}({{{{{{{\bf{r}}}}}}}},\, \, {\omega }_{f};t)$, which, in the Heisenberg picture, obey the equal-time commutation relations: $[\hat{{{{{{{{\bf{f}}}}}}}}}({{{{{{{\bf{r}}}}}}}},\, \, {\omega }_{f};t), \, \hat{{{{{{{{\bf{f}}}}}}}}}({{{{{{{{\bf{r}}}}}}}}}^{{\prime} },\, \, {\omega }_{f}^{{\prime} };t)]=[{\hat{{{{{{{{\bf{f}}}}}}}}}}^{{{{\dagger}}} }({{{{{{{\bf{r}}}}}}}},\, \, {\omega }_{f};t),\, {\hat{{{{{{{{\bf{f}}}}}}}}}}^{{{{\dagger}}} }({{{{{{{{\bf{r}}}}}}}}}^{{\prime} },\, \, {\omega }_{f}^{{\prime} };t)]=0$, and $[\hat{{{{{{{{\bf{f}}}}}}}}}({{{{{{{\bf{r}}}}}}}},\, \, {\omega }_{f};t),\, {\hat{{{{{{{{\bf{f}}}}}}}}}}^{{{{\dagger}}} }({{{{{{{{\bf{r}}}}}}}}}^{{\prime} },\, {\omega }_{f}^{{\prime} };t)]= \hat{{\mathbb{I}}}\delta [{{{{{{{\bf{r}}}}}}}}-{{{{{{{{\bf{r}}}}}}}}}^{{\prime} }]\delta [{\omega }_{f}-{\omega }_{f}^{{\prime} }]$, where $\hat{{\mathbb{I}}}$ is the identity operator.

To describe the dynamical behavior of the time-varying quantum system we assume a perturbative approach with a Hamiltonian given by $\hat{{{{{{{{\mathcal{H}}}}}}}}}={\hat{{{{{{{{\mathcal{H}}}}}}}}}}_{0}+{\hat{{{{{{{{\mathcal{H}}}}}}}}}}_{{{{{{{{\rm{T}}}}}}}}}$^43,49–51^, where ${\hat{{{{{{{{\mathcal{H}}}}}}}}}}_{0}$ represents the macroscopic body without time-modulation,

2 $${\hat{{{{{{{{\mathcal{H}}}}}}}}}}_{0}=\int\,{d}^{3}{{{{{{{\bf{r}}}}}}}}\int\nolimits_{0}^{+\infty }d{\omega }_{f}\hslash {\omega }_{f}{\hat{{{{{{{{\bf{f}}}}}}}}}}^{{{{\dagger}}} }({{{{{{{\bf{r}}}}}}}},\, \, {\omega }_{f};t)\cdot \hat{{{{{{{{\bf{f}}}}}}}}}({{{{{{{\bf{r}}}}}}}},\, \, {\omega }_{f};t),\,$$

while ${\hat{{{{{{{{\mathcal{H}}}}}}}}}}_{{{{{{{{\rm{T}}}}}}}}}$ accounts for the perturbation describing the changes induced by the time-modulation,

3 $${\hat{{{{{{{{\mathcal{H}}}}}}}}}}_{{{{{{{{\rm{T}}}}}}}}}=-\int\,{d}^{3}{{{{{{{\bf{r}}}}}}}}\hat{{{{{{{{{{{{\mathcal{P}}}}}}}}}}}}}({{{{{{{\bf{r}}}}}}}};t)\cdot \hat{{{{{{{{{{{{\mathcal{E}}}}}}}}}}}}}({{{{{{{\bf{r}}}}}}}};t).$$

Here, the polarization field operator, given by $\hat{{{{{{{{{{{{\mathcal{P}}}}}}}}}}}}}({{{{{{{\bf{r}}}}}}}};t)\equiv \int\nolimits_{0}^{t}d\tau \Delta \chi ({{{{{{{\bf{r}}}}}}}},\, \, t,\, \, \tau )\hat{{{{{{{{\bf{{{{{{{{\mathcal{E}}}}}}}}}}}}}}}}}({{{{{{{\bf{r}}}}}}}};\tau )$, is tied to the time-varying susceptibility of the medium Δ*χ*(**r**, *t*, *τ*)^43^, and the electric field operator $\hat{{{{{{{{\bf{{{{{{{{\mathcal{E}}}}}}}}}}}}}}}}}({{{{{{{\bf{r}}}}}}}};t)={\hat{{{{{{{{{{{{\mathcal{E}}}}}}}}}}}}}}^{(+)}({{{{{{{\bf{r}}}}}}}};t)+{\hat{{{{{{{{{{{{\mathcal{E}}}}}}}}}}}}}}^{(-)}({{{{{{{\bf{r}}}}}}}};t)$, whose positive-frequency component reads as,

4 $${\hat{{{{{{{{{{{{\mathcal{E}}}}}}}}}}}}}}^{(+)}({{{{{{{\bf{r}}}}}}}};t)=\int\nolimits_{0}^{+\infty }d{\omega }_{f}\int\,{d}^{3}{{{{{{{\boldsymbol{\rho }}}}}}}}\,{{{{{{{{\bf{G}}}}}}}}}_{{{{{{{{\rm{E}}}}}}}}}({{{{{{{\bf{r}}}}}}}},\, \, {{{{{{{\boldsymbol{\rho }}}}}}}},\, \, {\omega }_{f})\cdot \hat{{{{{{{{\bf{f}}}}}}}}}({{{{{{{\boldsymbol{\rho }}}}}}}},\, \, {\omega }_{f};t),\,$$

with ${{{{{{{{\bf{G}}}}}}}}}_{{{{{{{{\rm{E}}}}}}}}}({{{{{{{\bf{r}}}}}}}},\, \, {{{{{{{\boldsymbol{\rho }}}}}}}},\, \, {\omega }_{f})=i\sqrt{\hslash {\varepsilon }^{{\prime\prime} }({{{{{{{\boldsymbol{\rho }}}}}}}},\, {\omega }_{f})/\pi {\varepsilon }_{0}}{({\omega }_{f}/c)}^{2}{{{{{{{\bf{G}}}}}}}}({{{{{{{\bf{r}}}}}}}},\, {{{{{{{\boldsymbol{\rho }}}}}}}},\, {\omega }_{f})$ being the response function characterizing the background medium, **G**(**r**, ***ρ***, *ω*_*f*_) the dyadic Green’s function for the unmodulated system, and noticing that ${\hat{{{{{{{{{{{{\mathcal{E}}}}}}}}}}}}}}^{(-)}({{{{{{{\bf{r}}}}}}}};t)=[{\hat{{{{{{{{{{{{\mathcal{E}}}}}}}}}}}}}}^{(+)}({{{{{{{\bf{r}}}}}}}};t)]^{{{{\dagger}}} }$ [see Supplementary Information Section I]. Similar perturbation Hamiltonians are adopted for modeling other nonlinear quantum processes^52,53^. Moreover, it is implicitly assumed that the susceptibility function, Δ*χ*, is small enough so that it could be regarded as a perturbation to the background structure, and does not significantly affect to the quantization procedure^53^.

The emission spectrum, both in the far- and near-field regimes, is given by^54–58^

5 $${{{{{{{\mathcal{S}}}}}}}}({{{{{{{\bf{r}}}}}}}};\omega )={\langle {\hat{{{{{{{{\bf{E}}}}}}}}}}^{(+)}{({{{{{{{\bf{r}}}}}}}};\omega )}^{{{{\dagger}}} }\cdot {\hat{{{{{{{{\bf{E}}}}}}}}}}^{(+)}({{{{{{{\bf{r}}}}}}}};\omega )\rangle }_{{{{{{{{\rm{th}}}}}}}}},\,$$

where ${\hat{{{{{{{{\bf{E}}}}}}}}}}^{(+)}({{{{{{{\bf{r}}}}}}}};\omega )={{{{{{{{\mathcal{L}}}}}}}}}_{\omega }[{\hat{{{{{{{{{{{{\mathcal{E}}}}}}}}}}}}}}^{(+)}({{{{{{{\bf{r}}}}}}}},\, t)]$ is the Laplace’s transform of the electric field operator. By solving the Heisenberg equations of motion for the polaritonic operators, $i\hslash {\partial }_{t}\hat{{{{{{{{\mathcal{O}}}}}}}}}=[\hat{{{{{{{{\mathcal{O}}}}}}}}},\, \hat{{{{{{{{\mathcal{H}}}}}}}}}]$, performing an integral in the complex frequency plane, and rearranging the terms [see Supplementary Information Sections II and III], ${\hat{{{{{{{{\bf{E}}}}}}}}}}^{(+)}({{{{{{{\bf{r}}}}}}}};\omega )$ can be compactly written as follows:

6 $${\hat{{{{{{{{\bf{E}}}}}}}}}}^{(+)}({{{{{{{\bf{r}}}}}}}};\omega )=i\omega {\mu }_{0}\int\,{d}^{3}{{{{{{{\boldsymbol{\rho }}}}}}}}\,{{{{{{{\bf{G}}}}}}}}({{{{{{{\bf{r}}}}}}}},\, {{{{{{{\boldsymbol{\rho }}}}}}}},\, \omega )\cdot \hat{{{{{{{{\bf{j}}}}}}}}}({{{{{{{\boldsymbol{\rho }}}}}}}};\omega ),\,$$

with the current density operator,

7 $$\hat{{{{{{{{\bf{j}}}}}}}}}({{{{{{{\boldsymbol{\rho }}}}}}}};\omega )=2\omega \left[\sqrt{\pi \hslash {\varepsilon }_{0}{\varepsilon }^{{\prime\prime} }({{{{{{{\boldsymbol{\rho }}}}}}}},\, \, \omega )}\,{\hat{{{{{{{{\bf{f}}}}}}}}}}_{0}-i{{{{{{{{\mathcal{L}}}}}}}}}_{\omega }[\Delta \tilde{\chi }({{{{{{{\boldsymbol{\rho }}}}}}}},\, \, t)\hat{{{{{{{{\bf{{{{{{{{\mathcal{E}}}}}}}}}}}}}}}}}({{{{{{{\boldsymbol{\rho }}}}}}}};t)]\right],\,$$

where ${\hat{{{{{{{{\bf{f}}}}}}}}}}_{0}\equiv \hat{{{{{{{{\bf{f}}}}}}}}}({{{{{{{\boldsymbol{\rho }}}}}}}},\, \, \omega ;t=0)$. The first term in Eq. (7) corresponds to the currents associated with a system without time-modulation, while the second term represents the currents excited due to the time-modulation of the permittivity. In order to obtain Eq. (7), we have conducted a sharp but routine assumption, whereby the time-varying susceptibility exhibits a modulation which actually is local in time, i.e., $\Delta \chi ({{{{{{{\bf{r}}}}}}}},\, \, t,\, \, \tau )=\Delta \tilde{\chi }({{{{{{{\bf{r}}}}}}}},\, \, t)\delta [t-\tau ]$^3,5,43,59,60^. This simplifies the mathematical treatment, and allows us to use the aforementioned equal-time commutation relationships. Despite this particularization, it should be noted that the formalism is completely general, and a time-modulation with an arbitrary form would be feasible with the proper adoption of time-dependent commutation relations^49^.

Equations (5)–(7) provide a quantum framework for the computation of thermal emission spectra that is conceptually similar to the semiclassical treatment sketched above: fluctuating EM currents give rise to fluctuating thermal fields by means of the corresponding propagator (the dyadic Green’s function) through the entire medium. Notwithstanding, the use of a quantum formulation generalizes the semiclassical treatment, enabling the evaluation of thermal currents and fields in time-varying media, the calculation of purely quantum phenomena such as vacuum amplification effects, and, ultimately, a sound, self-consistent, and systematic formulation that directly arises from the assumptions on the Hamiltonian of the system, without the need of any semiclassical additions to the theory.

### Electromagnetic currents and correlations in time-varying macroscopic bodies

Next, we use this formalism to obtain a general form of the fluctuating currents excited in a macroscopic body whose permittivity is modulated in time, providing an extension to usual forms justified through the FDT. To this end, we first note that Eq. (7) is an implicit equation, where the current density operator is defined as a function of the electric field operator, which is itself generated by the current density operator. This fact makes a clear signature of the sharply intertwined dynamic of the system. At any rate, such an equation may be solved iteratively leading to a solution in the form of a series expansion of current density operators at different orders:

8 $$\hat{{{{{{{{\bf{j}}}}}}}}}({{{{{{{\boldsymbol{\rho }}}}}}}};\omega )=\mathop{\sum }\limits_{n=0}^{\infty }{\hat{{{{{{{{\bf{j}}}}}}}}}}_{n}({{{{{{{\boldsymbol{\rho }}}}}}}};\omega ).$$

Accordingly, the first three elements of the series can be explicitly written as [see Supplementary Information Section IV]:

9a $${\hat{{{{{{{{\bf{j}}}}}}}}}}_{0}=\omega \sqrt{4\pi \hslash {\varepsilon }_{0}{\varepsilon }^{{\prime\prime} }({{{{{{{\boldsymbol{\rho }}}}}}}},\, \omega )}\,\hat{{{{{{{{\bf{f}}}}}}}}}({{{{{{{\boldsymbol{\rho }}}}}}}},\, \omega ;t=0);$$

9b $$\begin{array}{l}{\hat{{{{{{{{\bf{j}}}}}}}}}}_{1}\propto \int\,{d}^{3}{{{{{{{{\boldsymbol{\rho }}}}}}}}}^{{\prime} }\int\,d{\omega }^{{\prime} }{\omega }^{{\prime} }\Delta \tilde{\chi }({{{{{{{\boldsymbol{\rho }}}}}}}},\, \omega -{\omega }^{{\prime} }){{{{{{{\bf{G}}}}}}}}({{{{{{{\boldsymbol{\rho }}}}}}}},\, {{{{{{{{\boldsymbol{\rho }}}}}}}}}^{{\prime} },\, {\omega }^{{\prime} }){\hat{{{{{{{{\bf{j}}}}}}}}}}_{0}({{{{{{{{\boldsymbol{\rho }}}}}}}}}^{{\prime} };{\omega }^{{\prime} })\\+\int\,{d}^{3}{{{{{{{{\boldsymbol{\rho }}}}}}}}}^{{\prime} }\int\,d{\omega }^{{\prime} }{\omega }^{{\prime} }\Delta \tilde{\chi }({{{{{{{\boldsymbol{\rho }}}}}}}},\, \omega -{\omega }^{{\prime} }){{{{{{{{\bf{G}}}}}}}}}^{*}({{{{{{{\boldsymbol{\rho }}}}}}}},\, {{{{{{{{\boldsymbol{\rho }}}}}}}}}^{{\prime} },\, {\omega }^{{\prime} }){\hat{{{{{{{{\bf{j}}}}}}}}}}_{0}^{{{{\dagger}}} }({{{{{{{{\boldsymbol{\rho }}}}}}}}}^{{\prime} };{\omega }^{{\prime} });\end{array}$$

9c $${\hat{{{{{{{{\bf{j}}}}}}}}}}_{2}\propto \int\,{d}^{3}{{{{{{{{\boldsymbol{\rho }}}}}}}}}^{{\prime} }\int\,d{\omega }^{{\prime} }{\omega }^{{\prime} }\Delta \tilde{\chi }({{{{{{{\boldsymbol{\rho }}}}}}}},\, \omega -{\omega }^{{\prime} }){{{{{{{\bf{G}}}}}}}}({{{{{{{\boldsymbol{\rho }}}}}}}},\, {{{{{{{{\boldsymbol{\rho }}}}}}}}}^{{\prime} },\, {\omega }^{{\prime} }){\hat{{{{{{{{\bf{j}}}}}}}}}}_{1}({{{{{{{{\boldsymbol{\rho }}}}}}}}}^{{\prime} };{\omega }^{{\prime} }).$$

These expressions for the current density operators, obtained within the first and second Born approximations, have a clear physical meaning: the current density operators of successive orders result from the fields generated by the preceding ones. Somehow, this can be understood as a sort of multiple-scattering process induced by the time-modulated perturbation of the medium properties, assuming that the triggering modulating field is that emerging from the fluctuating EM currents associated to the stationary (unperturbed) background medium. Specifically, a current density operator of order *n* at position ${{{{{{{{\boldsymbol{\rho }}}}}}}}}^{{\prime} }$ and frequency ${\omega }^{{\prime} }$, i.e., ${\hat{{{{{{{{\bf{j}}}}}}}}}}_{n}({{{{{{{{\boldsymbol{\rho }}}}}}}}}^{{\prime} };{\omega }^{{\prime} })$, generates a field at the position ***ρ*** via the propagation of the Green’s function ${{{{{{{\bf{G}}}}}}}}({{{{{{{\boldsymbol{\rho }}}}}}}},\, {{{{{{{{\boldsymbol{\rho }}}}}}}}}^{{\prime} },\, {\omega }^{{\prime} })$. Roughly speaking, at such a position, the action of the field over the time-varying susceptibility, $\Delta \tilde{\chi }({{{{{{{\boldsymbol{\rho }}}}}}}},\, \omega -{\omega }^{{\prime} })$, generates a higher-order current at frequency *ω*, i.e., ${\hat{{{{{{{{\bf{j}}}}}}}}}}_{n+1}({{{{{{{\boldsymbol{\rho }}}}}}}},\, \omega )$. As we will show, the interplay between sources of different order result in nontrivial correlations between fluctuating currents at different frequencies and points of space. Moreover, a crucial aspect of the current density operators is that they mix creation, ${\hat{{{{{{{{\bf{f}}}}}}}}}}^{{{{\dagger}}} }$, and annihilation, $\hat{{{{{{{{\bf{f}}}}}}}}}$, polaritonic operators, akin to Bogoliubov (or squeezing) transformations^61^. The difference between creation and annihilation operators, not present in semiclassical treatments, allows for a rigorous benchmark in order to properly include the quantum vacuum contribution^34^.

Subsequently, the correlation between the fluctuating EM currents at different frequencies and points of space can also be written in a series form:

10 $${\left\langle {\hat{{{{{{{{\bf{j}}}}}}}}}}^{{{{\dagger}}} }({{{{{{{\boldsymbol{\rho }}}}}}}};\omega )\cdot \hat{{{{{{{{\bf{j}}}}}}}}}({{{{{{{{\boldsymbol{\rho }}}}}}}}}^{{\prime} };{\omega }^{{\prime} })\right\rangle }_{{{{{{{{\rm{th}}}}}}}}}=\mathop{\sum}\limits_{l,\, m}{\left\langle {\hat{{{{{{{{\bf{j}}}}}}}}}}_{l}^{{{{\dagger}}} }({{{{{{{\boldsymbol{\rho }}}}}}}};\omega )\cdot {\hat{{{{{{{{\bf{j}}}}}}}}}}_{m}({{{{{{{{\boldsymbol{\rho }}}}}}}}}^{{\prime} };{\omega }^{{\prime} })\right\rangle }_{{{{{{{{\rm{th}}}}}}}}}.$$

With this in mind, the corresponding *n*th-order correlations can be obtained from a direct evaluation of ${\langle {\hat{{{{{{{{\bf{j}}}}}}}}}}_{l}^{{{{\dagger}}} }({{{{{{{\boldsymbol{\rho }}}}}}}};\omega )\cdot {\hat{{{{{{{{\bf{j}}}}}}}}}}_{m}({{{{{{{{\boldsymbol{\rho }}}}}}}}}^{{\prime} };{\omega }^{{\prime} })\rangle }_{{{{{{{{\rm{th}}}}}}}}}$, where *l* + *m* = *n*, and ${\langle \cdots \rangle }_{{{{{{{{\rm{th}}}}}}}}}\equiv {{{{{{{\rm{Tr}}}}}}}}[\cdots {\hat{\varrho }}_{{{{{{{{\rm{th}}}}}}}}}]$, with ${\hat{\varrho }}_{{{{{{{{\rm{th}}}}}}}}}$ being the thermal density operator that yields the thermal fields at a given temperature *T*^49,50,53^.

As expected, the zeroth-order correlation of the current density, ${\langle {\hat{{{{{{{{\bf{j}}}}}}}}}}_{0}^{{{{\dagger}}} }\cdot {\hat{{{{{{{{\bf{j}}}}}}}}}}_{0}\rangle }_{{{{{{{{\rm{th}}}}}}}}}$, coincides with the original version of the FDT given in Eq. (1). In other words, our quantum formalism correctly recovers the semiclassical case for a stationary (non-time-modulated) system. At the same time, it generalizes this result via the higher-order correlations. Indeed, proceeding iteratively, one can find closed form expressions for such higher-order contributions to the current density correlations [see Supplementary Information Section IV]. The corresponding results for the first and second-order contributions are presented in the Table 1.

**Table 1:** Fluctuation-dissipation theorem for time-varying systems: First and second-order current density correlations

$$\begin{array}{l}\hskip4pt{\langle {\hat{{{{{{{{\bf{j}}}}}}}}}}_{0}^{{{{\dagger}}} }({{{{{{{\boldsymbol{\rho }}}}}}}};\omega )\cdot {\hat{{{{{{{{\bf{j}}}}}}}}}}_{1}({{{{{{{{\boldsymbol{\rho }}}}}}}}}^{{\prime} };{\omega }^{{\prime} })\rangle }_{{{{{{{{\rm{th}}}}}}}}} \,=\, {\omega }^{{\prime} }\left[\frac{{\mu }_{0}}{\pi }\right]{\int}_{{{{{{{{\mathcal{V}}}}}}}}}{d}^{3}{{{{{{{{\boldsymbol{\rho }}}}}}}}}^{{\prime\prime} }\int\,d{\omega }^{{\prime\prime} }{\omega }^{{\prime\prime} }\Delta \tilde{\chi }({{{{{{{{\boldsymbol{\rho }}}}}}}}}^{{\prime} },{\omega }^{{\prime} }-{\omega }^{{\prime\prime} }){{{{{{{\bf{G}}}}}}}}({{{{{{{{\boldsymbol{\rho }}}}}}}}}^{{\prime} },{{{{{{{{\boldsymbol{\rho }}}}}}}}}^{{\prime\prime} },{\omega }^{{\prime\prime} }){\langle {\hat{{{{{{{{\bf{j}}}}}}}}}}_{0}^{{{{\dagger}}} }({{{{{{{\boldsymbol{\rho }}}}}}}};\omega )\cdot {\hat{{{{{{{{\bf{j}}}}}}}}}}_{0}({{{{{{{{\boldsymbol{\rho }}}}}}}}}^{{\prime\prime} };{\omega }^{{\prime\prime} })\rangle }_{{{{{{{{\rm{th}}}}}}}}};\\ {\langle {\hat{{{{{{{{\bf{j}}}}}}}}}}_{0}^{{{{\dagger}}} }({{{{{{{\boldsymbol{\rho }}}}}}}};\omega )\cdot {\hat{{{{{{{{\bf{j}}}}}}}}}}_{2}({{{{{{{{\boldsymbol{\rho }}}}}}}}}^{{\prime} };{\omega }^{{\prime} })\rangle }_{{{{{{{{\rm{th}}}}}}}}}=\, {\omega }^{{\prime} }\left[\frac{{\mu }_{0}}{\pi }\right]{\int}_{{{{{{{{\mathcal{V}}}}}}}}}{d}^{3}{{{{{{{{\boldsymbol{\rho }}}}}}}}}^{{\prime\prime} }\int\,d{\omega }^{{\prime\prime} }{\omega }^{{\prime\prime} }\Delta \tilde{\chi }({{{{{{{{\boldsymbol{\rho }}}}}}}}}^{{\prime} },{\omega }^{{\prime} }-{\omega }^{{\prime\prime} }){{{{{{{\bf{G}}}}}}}}({{{{{{{{\boldsymbol{\rho }}}}}}}}}^{{\prime} },{{{{{{{{\boldsymbol{\rho }}}}}}}}}^{{\prime\prime} },{\omega }^{{\prime\prime} }){\langle {\hat{{{{{{{{\bf{j}}}}}}}}}}_{0}^{{{{\dagger}}} }({{{{{{{\boldsymbol{\rho }}}}}}}};\omega )\cdot {\hat{{{{{{{{\bf{j}}}}}}}}}}_{1}({{{{{{{{\boldsymbol{\rho }}}}}}}}}^{{\prime\prime} };{\omega }^{{\prime\prime} })\rangle }_{{{{{{{{\rm{th}}}}}}}}}\\ \hskip5pt {\langle {\hat{{{{{{{{\bf{j}}}}}}}}}}_{1}^{{{{\dagger}}} }({{{{{{{\boldsymbol{\rho }}}}}}}};\omega )\cdot {\hat{{{{{{{{\bf{j}}}}}}}}}}_{1}({{{{{{{{\boldsymbol{\rho }}}}}}}}}^{{\prime} };{\omega }^{{\prime} })\rangle }_{{{{{{{{\rm{th}}}}}}}}} \,=\, \omega {\omega }^{{\prime} }{\left[\frac{{\mu }_{0}}{\pi }\right]}^{2}{\iint }_{{{{{{{{\mathcal{V}}}}}}}}}{d}^{3}\tilde{{{{{{{{\boldsymbol{\rho }}}}}}}}}{d}^{3}{\tilde{{{{{{{{\boldsymbol{\rho }}}}}}}}}}^{{\prime} }\iint d\tilde{\omega }d{\tilde{\omega }}^{{\prime} }\tilde{\omega }{\tilde{\omega }}^{{\prime} }\Delta {\tilde{\chi }}^{*}({{{{{{{\boldsymbol{\rho }}}}}}}},\omega -\tilde{\omega })\Delta \tilde{\chi }({{{{{{{{\boldsymbol{\rho }}}}}}}}}^{{\prime} },{\omega }^{{\prime} }-{\tilde{\omega }}^{{\prime} })\hfill\\ \left[{{{{{{{{\bf{G}}}}}}}}}^{*}({{{{{{{\boldsymbol{\rho }}}}}}}},\tilde{{{{{{{{\boldsymbol{\rho }}}}}}}}},\tilde{\omega }){{{{{{{\bf{G}}}}}}}}({{{{{{{{\boldsymbol{\rho }}}}}}}}}^{{\prime} },{\tilde{{{{{{{{\boldsymbol{\rho }}}}}}}}}}^{{\prime} },{\tilde{\omega }}^{{\prime} }){\langle {\hat{{{{{{{{\bf{j}}}}}}}}}}_{0}^{{{{\dagger}}} }(\tilde{{{{{{{{\boldsymbol{\rho }}}}}}}}};\tilde{\omega })\cdot {\hat{{{{{{{{\bf{j}}}}}}}}}}_{0}({\tilde{{{{{{{{\boldsymbol{\rho }}}}}}}}}}^{{\prime} };{\tilde{\omega }}^{{\prime} })\rangle }_{{{{{{{{\rm{th}}}}}}}}}+{{{{{{{\bf{G}}}}}}}}({{{{{{{\boldsymbol{\rho }}}}}}}},\tilde{{{{{{{{\boldsymbol{\rho }}}}}}}}},\tilde{\omega }){{{{{{{{\bf{G}}}}}}}}}^{*}({{{{{{{{\boldsymbol{\rho }}}}}}}}}^{{\prime} },{\tilde{{{{{{{{\boldsymbol{\rho }}}}}}}}}}^{{\prime} },{\tilde{\omega }}^{{\prime} }){\langle {\hat{{{{{{{{\bf{j}}}}}}}}}}_{0}(\tilde{{{{{{{{\boldsymbol{\rho }}}}}}}}};\tilde{\omega })\cdot {\hat{{{{{{{{\bf{j}}}}}}}}}}_{0}^{{{{\dagger}}} }({\tilde{{{{{{{{\boldsymbol{\rho }}}}}}}}}}^{{\prime} };{\tilde{\omega }}^{{\prime} })\rangle }_{{{{{{{{\rm{th}}}}}}}}}\right]\hskip-175pt\end{array}$$	

Comparing this result with the original form of the FDT for stationary systems, one may realize that, even truncating the expansion at the second order, time-varying media bring new physics to fluctuational electrodynamics. First feature concerns to the breakdown of locality, since higher-order fluctuating currents are correlated at different frequencies and position of space. For conventional (non-time-modulated) thermal emitters, the correlations are local both in position and frequency. This becomes evident at a glance from the involvement of the Dirac delta functions [see Eq. (1)], and is physically understood as a consequence of the random nature of the thermal fields. However, higher-order terms include integrals over frequencies and positions. In this manner, the time-modulation enables the possibility of correlating (or connecting) different frequencies appearing at different locations of the material system, thus underscoring its non-local character and, consequently, the potential to enhance the coherence of thermal fields. Importantly, inasmuch as the correlations rely on the Green’s function formalism, along with the truly dispersive character of the background material including the absorption losses, and hence satisfying the Kramers-Kronig relations, this non-local behavior is perfectly consistent with the causality^37^.

There is an additional feature that affects to the photon distribution. Indeed, in the ${\langle {\hat{{{{{{{{\bf{j}}}}}}}}}}_{1}^{{{{\dagger}}} }\cdot {\hat{{{{{{{{\bf{j}}}}}}}}}}_{1}\rangle }_{{{{{{{{\rm{th}}}}}}}}}$ term, it can be seen that the black-body spectrum, Θ(*ω*, *T*), appears inside a frequency integral. For conventional thermal emitters, the spectrum of thermal radiation is given by *I*_real_(*ω*, *T*) = *α*(*ω*)*I*_BB_(*ω*, *T*), where *α*(*ω*) = *ϵ*(*ω*) ≤ 1 is the spectral absorptivity (related to the emissivity by the Kirchhoff’s radiation law), and *I*_BB_ ∝ Θ(*ω*, *T*) refers to the black-body emission spectrum. Herein, Θ(*ω*, *T*) acts as a fixed frequency window that ultimately sets an upper limit for the radiative heat transfer. Thus, customary spatial-like nanophotonic engineering of far-field emission spectra have so far been limited to the control of the optical absorptivity (or the emissivity) of materials^62,63^, within the limits imposed by the black-body spectrum [see Fig. 1(b)]. By contrast, our analysis reveals that time-modulated thermal emitters can modify and even overcome the black-body’s photon distribution, thus making tunable the accessible window of frequencies [see Fig. 1(c)].

Another crucial aspect of the ${\langle {\hat{{{{{{{{\bf{j}}}}}}}}}}_{1}^{{{{\dagger}}} }\cdot {\hat{{{{{{{{\bf{j}}}}}}}}}}_{1}\rangle }_{{{{{{{{\rm{th}}}}}}}}}$ term, is that it also contains anti-normally ordered correlations ${\langle {\hat{{{{{{{{\bf{j}}}}}}}}}}_{0}\cdot {\hat{{{{{{{{\bf{j}}}}}}}}}}_{0}^{{{{\dagger}}} }\rangle }_{{{{{{{{\rm{th}}}}}}}}}$^64,65^, indicating so the eventual occurrence of dynamical vacuum amplifications effects^44^. In fact, this term is the dominant contribution in the zero-temperature limit (T → 0 K). In this sense, our quantum formalism goes beyond the semiclassical approaches, allowing for unifying quantum photon production (such as the dynamical Casimir effect^42,43^ or parametric amplification^44^) and thermal emission processes, thus paving the way to the study of vacuum amplification effects at finite temperature, which might be required for the analysis of realistic configurations. Then, besides thermal emission, our quantum formalism for fluctuating EM currents sets the basis for addressing quantum vacuum forces in time-varying media, such as Casimir forces^41^ and quantum friction^66–71^.

### Thermal emission spectra in time-modulated materials

#### A simple model: semi-infinite dielectric planar slab

**Fig. 3:**
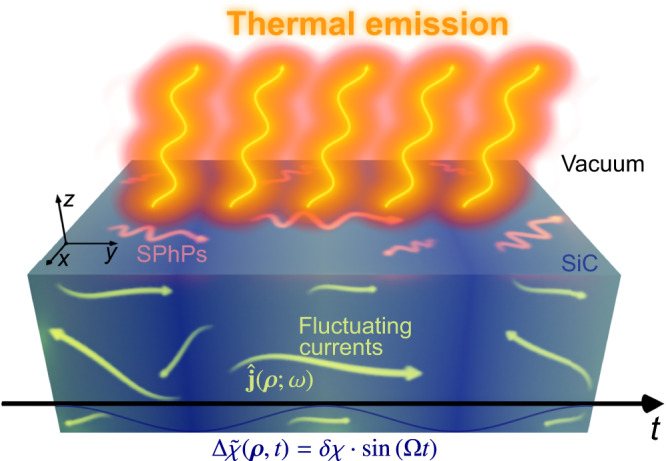
Thermal emission from a semi-infinite planar slab of SiC subjected to a harmonic time-modulation. Schematic depiction of a semi-infinite slab of SiC at temperature *T* with a random distribution of fluctuating currents moving inside. The horizontal axis represents the time, so that each section displays a different susceptibility.

Thus far we have sketched out the theoretical model of thermally fluctuating EM currents and their correlations in time-varying media. Next, we illustrate some of the main consequences of such a time-modulation in connection with the thermal emission spectra. To this end, we revisit a historical example that helped initiating the field of nanophotonic engineering of thermal emission^32–34^. It consists of a silicon carbide (SiC) substrate (*z* < 0), in contact with vacuum (*z* > 0) [see Fig. 3]. The frequency-dependent permittivity of SiC is described by a Drude-Lorentz model, so that $\varepsilon (\omega )={\varepsilon }_{\infty }({\omega }_{L}^{2}-{\omega }^{2}-i\gamma \omega )/({\omega }_{T}^{2}-{\omega }^{2}-i\gamma \omega )$, where *ε*_*∞*_ = 6.7, *ω*_*L*_ = 29.1 THz, *ω*_*T*_ = 23.8 THz, and *γ* = 0.14 THz^33^, stand, respectively, for the high-frequency-limit permittivity, the longitudinal and transverse optical phonon frequencies, and the damping factor (or characteristic collision frequency). Because of such a frequency dispersion, a SiC substrate supports nontrivial far and near-field thermal fluctuations, including the thermal excitation of surface phonon polaritons (SPhPs). Here, instead of introducing a grating to outcouple near-field thermal waves^29^, we consider a time-harmonic modulation of the susceptibility: $\Delta \tilde{\chi }({{{{{{{\boldsymbol{\rho }}}}}}}},\, t)={\varepsilon }_{0}\delta \chi \sin \Omega t$. As shown below, this time-modulation-based configuration shall aid the unveiling of unique wave phenomena.

#### Zeroth-order correlations: Ground contribution

Using the Green’s function formalism [see Methods and Supplementary Information Sections V and VI for further details], the zeroth-order contribution to the thermal emission spectrum can be obtained from the corresponding electric field correlation:

11 $${{{{{{{\boldsymbol{{{{{\mathcal{S}}}}}}}}}}}}}_{0}({{{{{{{\bf{r}}}}}}}};\omega ;T)=\frac{4\pi }{{\varepsilon }_{0}}\frac{{\omega }^{3}}{{c}^{4}}{\varepsilon }^{{\prime\prime} }(\omega )\hslash \omega \Theta (\omega,\, T){{{{{{{{\mathcal{G}}}}}}}}}_{0,\, 0}({{{{{{{\bf{r}}}}}}}},\, \omega ),\,$$

with

12 $${{{{{{{{\mathcal{G}}}}}}}}}_{0,\, 0}=\frac{{k}_{0}^{2}}{2\pi }\int\nolimits_{-\infty }^{0}d{\rho }_{z}\int\nolimits_{0}^{+\infty }d{\kappa }_{R}{\kappa }_{R}{\left|{\hat{{{{{{{{\bf{G}}}}}}}}}}_{1\leftarrow 2}({{{{{{{\bf{k}}}}}}}};\omega|z,\, {\rho }_{z})\right|}_{{{{{{{{\mathcal{F}}}}}}}}}^{2},\,$$

where the subscript ${{{{{{{\mathcal{F}}}}}}}}$ stands for the Frobenius norm. As anticipated, this zeroth-order term recovers the spectrum of a stationary (non-time-modulated) material^33,34^.

**Fig. 4:**
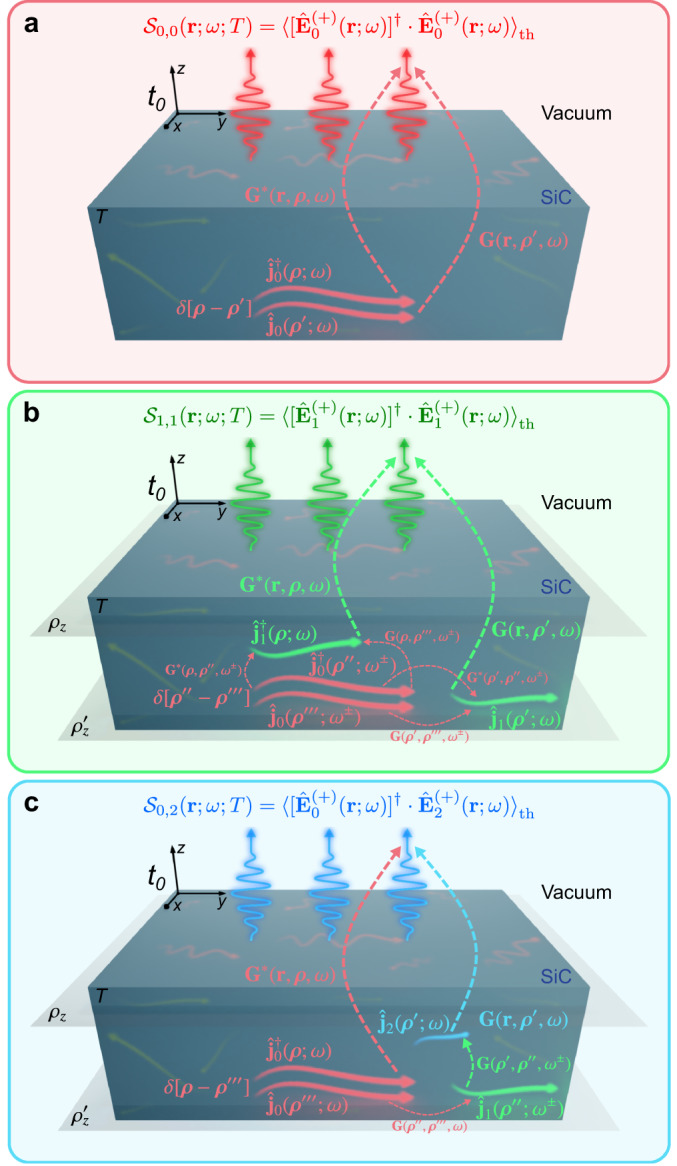
Physical picture of the field correlations. **a** Conceptual representation of the local processes contributing to the zeroth-order of correlations (${{{{{{{{\mathcal{S}}}}}}}}}_{0,0}$). **b** Depiction of the non-local processes giving rise to the ${{{{{{{{\mathcal{S}}}}}}}}}_{1,1}$ contribution to the second-order of correlations. **c** Contribution ${{{{{{{{\mathcal{S}}}}}}}}}_{0,2}$ to the second-order of correlations. The two planes at *ρ*_*z*_ and ${\rho }_{z}^{{\prime} }$ separate different regions to distinguish among the possible cases that may occur. Red, green, and blue colors represent zeroth, first, and second order features (fields, currents, or propagators), respectively.

Figure 4 (a) schematically depicts a physical picture of this zeroth-order correlation-emission process. Since zeroth-order fluctuating currents are uncorrelated in space and frequency (meaning that the correlations are local, accounted by $\delta [{{{{{{{\boldsymbol{\rho }}}}}}}}-{{{{{{{{\boldsymbol{\rho }}}}}}}}}^{{\prime} }]$ and $\delta [\omega -{\omega }^{{\prime} }]$), the spectrum of thermal radiation can be reconstructed by adding the individual contributions of the fluctuating currents ${\hat{{{{{{{{\bf{j}}}}}}}}}}_{0}({{{{{{{\boldsymbol{\rho }}}}}}}};\omega )$ at each point of space ***ρ***, for a fixed frequency *ω* [see Fig. 4(a)]. Such a procedure is characterized by means of the (norm of the) dyadic Green’s function, integrated all along the lower half-space *ρ*_*z*_ ≤ 0, as indicated in Eq. (12).

#### Second-order correlations: Time-modulated term

Taking into account that the first-order term does not effectively contribute to the thermal emission spectrum [see Supplementary Information Section V for details], the second-order correlation is the first contribution to the emission spectrum yielding an explicit dependence of the time-modulation:

13 $${{{{{{{\boldsymbol{{{{{\mathcal{S}}}}}}}}}}}}}_{2}({{{{{{{\bf{r}}}}}}}};\omega ;T)={{{{{{{\boldsymbol{{{{{\mathcal{S}}}}}}}}}}}}}_{1,\, 1}({{{{{{{\bf{r}}}}}}}};\omega ;T)+2{{{{{{{\rm{Re}}}}}}}}[{{{{{{{\boldsymbol{{{{{\mathcal{S}}}}}}}}}}}}}_{0,\, 2}({{{{{{{\bf{r}}}}}}}};\omega ;T)],\,$$

noticing that $${{{{{{{\boldsymbol{{{{{\mathcal{S}}}}}}}}}}}}}_{2,\, 0}({{{{{{{\bf{r}}}}}}}};\omega ;T)={[{{{{{{{\boldsymbol{{{{{\mathcal{S}}}}}}}}}}}}}_{0,\, 2}({{{{{{{\bf{r}}}}}}}};\omega ;T)]}^{*}$$.

The first term in Eq. (13) is associated with the ${\langle {\hat{{{{{{{{\bf{j}}}}}}}}}}_{1}^{{{{\dagger}}} }\cdot {\hat{{{{{{{{\bf{j}}}}}}}}}}_{1}\rangle }_{{{{{{{{\rm{th}}}}}}}}}$ correlation function, and is given by [see Supplementary Information Section VI]:

14 $${{{{{{{\boldsymbol{{{{{\mathcal{S}}}}}}}}}}}}}_{1,\, 1}({{{{{{{\bf{r}}}}}}}};\omega ;T)={{{{{{{\boldsymbol{{{{{\mathcal{S}}}}}}}}}}}}}_{1,\, 1}^{+}+{{{{{{{\boldsymbol{{{{{\mathcal{S}}}}}}}}}}}}}_{1,\, 1}^{-},\,$$

with

15 $${{{{{{{\boldsymbol{{{{{\mathcal{S}}}}}}}}}}}}}_{1,\, 1}^{\pm }=\frac{4\pi \delta {\chi }^{2}}{{\varepsilon }_{0}}\frac{{\omega }^{5}}{{c}^{8}}{\varepsilon }^{{\prime\prime} }({\omega }^{\pm })\hslash {\omega }^{\pm }\Theta ({\omega }^{\pm }\!,\, T){({\omega }^{\pm })}^{2}{{{{{{{{\mathcal{G}}}}}}}}}_{1,\, 1}^{(\pm )},\,$$

and

16 $${{{{{{{{\mathcal{G}}}}}}}}}_{1,\, 1}^{(\pm )}\equiv (1+\tilde{\Omega })\left[{\bar{{{{{{{{\mathcal{G}}}}}}}}}}_{1,\, 1}^{(\pm )}+{\tilde{{{{{{{{\mathcal{G}}}}}}}}}}_{1,\, 1}^{(\pm )}+{[\Theta ({\omega }^{\pm } \!,\, T)]}^{-1}{\tilde{{{{{{{{\mathcal{G}}}}}}}}}}_{1,\, 1}^{(\pm )}\right].$$

Here, we have defined the shifted frequency *ω*^±^ ≡ *ω* ± Ω, and ${\bar{{{{{{{{\mathcal{G}}}}}}}}}}_{1,\, 1}^{(\pm )}\equiv {\iiint }_{{{{{{{{\mathcal{V}}}}}}}}}{d}^{3}{{{{{{{\boldsymbol{\rho }}}}}}}}{d}^{3}{{{{{{{{\boldsymbol{\rho }}}}}}}}}^{{\prime} }{d}^{3}{{{{{{{{\boldsymbol{\rho }}}}}}}}}^{{\prime\prime} }{{{{{{{\rm{Tr}}}}}}}}[{{{{{{{{\bf{G}}}}}}}}}^{*}({{{{{{{\bf{r}}}}}}}},\, {{{{{{{\boldsymbol{\rho }}}}}}}},\, \omega ){{{{{{{{\bf{G}}}}}}}}}^{*}({{{{{{{\boldsymbol{\rho }}}}}}}},\, {{{{{{{{\boldsymbol{\rho }}}}}}}}}^{{\prime\prime} },\, {\omega }^{\pm }){{{{{{{\bf{G}}}}}}}}({{{{{{{\bf{r}}}}}}}},\, {{{{{{{{\boldsymbol{\rho }}}}}}}}}^{{\prime} },\, \omega ){{{{{{{\bf{G}}}}}}}}({{{{{{{{\boldsymbol{\rho }}}}}}}}}^{{\prime} },\, {{{{{{{{\boldsymbol{\rho }}}}}}}}}^{{\prime\prime} },\, {\omega }^{\pm })]$, ${\tilde{{{{{{{{\mathcal{G}}}}}}}}}}_{1,\, 1}^{(\pm )}\equiv {\iiint }_{{{{{{{{\mathcal{V}}}}}}}}}{d}^{3}{{{{{{{\boldsymbol{\rho }}}}}}}}{d}^{3}{{{{{{{{\boldsymbol{\rho }}}}}}}}}^{{\prime} }{d}^{3}{{{{{{{{\boldsymbol{\rho }}}}}}}}}^{{\prime\prime} }{{{{{{{\rm{Tr}}}}}}}}\ [\,{{{{{{{{\bf{G}}}}}}}}}^{*}({{{{{{{\bf{r}}}}}}}},\, {{{{{{{\boldsymbol{\rho }}}}}}}},\, \omega )\ {{{{{{{\bf{G}}}}}}}}({{{{{{{\boldsymbol{\rho }}}}}}}},\, {{{{{{{{\boldsymbol{\rho }}}}}}}}}^{{\prime\prime} },\, {\omega }^{\pm })\ {{{{{{{\bf{G}}}}}}}}({{{{{{{\bf{r}}}}}}}},\, {{{{{{{{\boldsymbol{\rho }}}}}}}}}^{{\prime} },\, \omega )\ {{{{{{{{\bf{G}}}}}}}}}^{*}({{{{{{{{\boldsymbol{\rho }}}}}}}}}^{{\prime} },\, {{{{{{{{\boldsymbol{\rho }}}}}}}}}^{{\prime\prime} },\, {\omega }^{\pm })]$, and $\tilde{\Omega }\equiv \Omega /\omega$.

Similarly, the second term in Eq. (13) is associated with the ${\langle {\hat{{{{{{{{\bf{j}}}}}}}}}}_{0}^{{{{\dagger}}} }\cdot {\hat{{{{{{{{\bf{j}}}}}}}}}}_{2}\rangle }_{{{{{{{{\rm{th}}}}}}}}}$ correlation function, and it is given by [see Supplementary Information Section VI]:

17 $${{{{{{{\boldsymbol{{{{{\mathcal{S}}}}}}}}}}}}}_{0,\, 2}({{{{{{{\bf{r}}}}}}}};\omega ;T)={{{{{{{\boldsymbol{{{{{\mathcal{S}}}}}}}}}}}}}_{0,\, 2}^{+}+{{{{{{{\boldsymbol{{{{{\mathcal{S}}}}}}}}}}}}}_{0,\, 2}^{-},\,$$

where

18 $${{{{{{{\boldsymbol{{{{{\mathcal{S}}}}}}}}}}}}}_{0,\, 2}^{\pm }=\frac{4\pi \delta {\chi }^{2}}{{\varepsilon }_{0}}\frac{{\omega }^{5}}{{c}^{8}}{\varepsilon }^{{\prime\prime} }(\omega )\hslash \omega \Theta (\omega,\, T){({\omega }^{\pm })}^{2}{{{{{{{{\mathcal{G}}}}}}}}}_{0,\, 2}^{(\pm )},\,$$

with $${{{{{{{{\mathcal{G}}}}}}}}}_{0,\, 2}^{(\pm )}\equiv {\iiint }_{{{{{{{{\mathcal{V}}}}}}}}}{d}^{3}{{{{{{{\boldsymbol{\rho }}}}}}}}{d}^{3}{{{{{{{{\boldsymbol{\rho }}}}}}}}}^{{\prime} }{d}^{3}{{{{{{{{\boldsymbol{\rho }}}}}}}}}^{{\prime\prime} }{{{{{{{\rm{Tr}}}}}}}}[{{{{{{{{\bf{G}}}}}}}}}^{*}({{{{{{{\bf{r}}}}}}}},\, {{{{{{{\boldsymbol{\rho }}}}}}}},\, \omega ){{{{{{{\bf{G}}}}}}}}({{{{{{{\bf{r}}}}}}}},\, {{{{{{{{\boldsymbol{\rho }}}}}}}}}^{{\prime} },\, \omega ){{{{{{{\bf{G}}}}}}}}({{{{{{{{\boldsymbol{\rho }}}}}}}}}^{{\prime} },\, {{{{{{{{\boldsymbol{\rho }}}}}}}}}^{{\prime\prime} },\, {\omega }^{\pm }){{{{{{{\bf{G}}}}}}}}({{{{{{{{\boldsymbol{\rho }}}}}}}}}^{{\prime\prime} },\, {{{{{{{\boldsymbol{\rho }}}}}}}},\, \omega )]$$.

While the expressions for the second-order correlations are mathematically involved, they have a clear physical meaning. Indeed, such a correlation-emission process is schematically illustrated in Fig. 4(b, c) for each of the two contributions given in Eq. (13).

Specifically, as can be seen in Fig. 4b, for the ${{{{{{{\boldsymbol{{{{{\mathcal{S}}}}}}}}}}}}}_{1,\, 1}({{{{{{{\bf{r}}}}}}}};\omega ;T)$ contribution, the zeroth-order current ${\hat{{{{{{{{\bf{j}}}}}}}}}}_{0}$ at one point of space ***ρ***^*″*^ generates first-order current densities ${\hat{{{{{{{{\bf{j}}}}}}}}}}_{1}$ at different positions ***ρ*** and ${{{{{{{{\boldsymbol{\rho }}}}}}}}}^{{\prime} }$. Despite emerging from fluctuating currents at different positions, the non-locality induced by the time modulation allows for nontrivial correlations and a nonzero contribution for the emission spectrum. Furthermore, it is important to realize about the aforementioned quantum vacuum contribution, which, as shown below, brings about the dominant contribution to the time-modulated terms.

On the other side, as shown in Fig. 4c, the situation is slightly different for the ${{{{{{{\boldsymbol{{{{{\mathcal{S}}}}}}}}}}}}}_{0,\, 2}({{{{{{{\bf{r}}}}}}}};\omega ;T)$ contribution. In this case, there are two hopping processes, so that an original current ${\hat{{{{{{{{\bf{j}}}}}}}}}}_{0}$, induces a first-order current ${\hat{{{{{{{{\bf{j}}}}}}}}}}_{1}$, which in turn induces a second-order current ${\hat{{{{{{{{\bf{j}}}}}}}}}}_{2}$. Then, by means of a nontrivial correlation, the second-order and the original zeroth-order EM currents mix and match to each other to generate the radiative fields.

As deliberately depicted in the representations, this second-order correlations are spatially non-local. This feature is a direct consequence of the temporal modulation of the susceptibility, and means that the correlations are associated to currents that may be spatially separated from each other.

## Discussion

### Thermal phenomena in time-modulated media

**Fig. 5:**
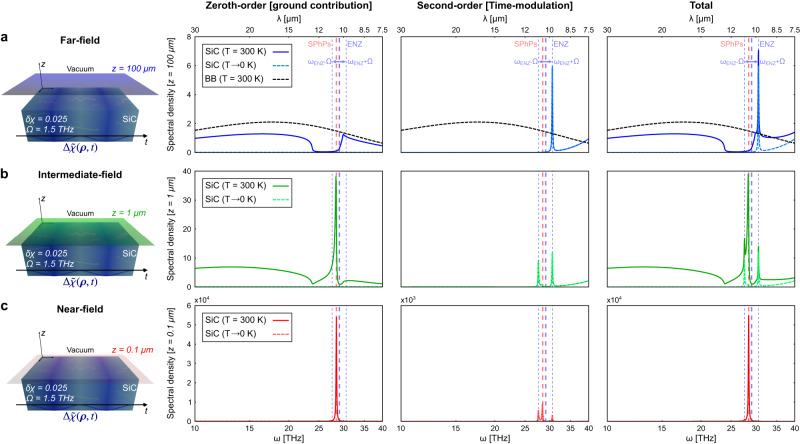
Emission spectra of a semi-infinite planar slab made of SiC with a time-varying susceptibility externally perturbed under a time-harmonic modulation (Ω = 1.5 THz and *δχ* = 0.025). At finite temperature (T = 300 K), thermal emission spectra display remarkable differences depending on the regime in which one perform the measurement: **a** far-field (100 *μ*m); **b** intermediate-field (1 *μ*m); **c** near-field (0.1 *μ*m). The physical features brought about by the time-modulation lead to an enhancement of the emission spectra in the ENZ frequency regime (noticing their shift due to the modulation), which results as a direct consequence of the non-local character of the current density correlations in time-varying media. This enables a mechanism for overcoming the black-body spectrum in the far-field (dashed black curves), as well as for connecting near-to-far field radiation effects. Likewise, the spectra at T → 0 K show the potential of time-modulation to produce dynamical vacuum amplification effects at the same time that reveal the quantum nature of the ENZ-induced strong releasing of radiation.

Upon this ground, we can now address the emission spectra of the time-modulated SiC as a function of the distance *z* above the interface. This is shown in Fig. 5, where we plot separately each of the contributions associated to the zeroth and the second order, as well as the total spectrum at *z* = 100 *μ*m (far-field), *z* = 1 *μ*m (intermediate-field), and *z* = 0.1 *μ*m (near-field), assuming the temperature of the body to be at T = 300 K and in the limit T → 0 K.

At finite temperature, the zeroth-order term is associated to the free-evolving and non-perturbed part of the system, and, as anticipated, reproduces previous semiclassical results^33,34^. In the far-zone, thermal emission is characterized by the black-body spectrum weighted over the emissivity of the substrate. Here, the main feature is a band of minimum emission associated with the Reststrahlen band of SiC^72^, where the permittivity is negative *ε* < 0. In the near-field regime, thermal radiation is dominated by the resonant excitation of SPhPs, with a very narrow band spectrum centered at the frequency for which *ε* ≃ − 1 (dashed red line).

On the other hand, the second-order spectra are tied to the perturbed harmonic time-modulation of the susceptibility, where it has been assumed that Ω = 1.5 THz and *δχ* = 0.025. From the thermal emission spectra shown in Fig. 5, we firstly highlight the appearance of a sharp peak in the far-field regime, centered at *ω*_ENZ_ + Ω. That is, it appears at the epsilon-near-zero (ENZ) frequency (*ω*_ENZ_ for which *ε* ≃ 0), shifted by the temporal modulation frequency Ω. Since this narrowband emission peak originates from a pure time-modulation of the substrate, it offers a greater flexibility and frequency agile capabilities than the nanofabrication engineering of thermal emitters. In addition, it constitutes a remarkable wave phenomenon with some distinctive properties.

#### ENZ-induced strong releasing of thermal fluctuations

First, we stress out that, according to our formalism, the appearance of the sharp emission peak is associated with strong fluctuations trapped within the material body, which are released via the temporal modulation. Indeed, inspecting the Green’s function one may realize that, within a same medium, ∣**G**(**r**, ***ρ***, *ω*)∣^2^ → ∣*ε*^*″*^(*ω*)∣^−2^ when *ε*(*ω*) → 0. Then, taking into account that the source contribution ${\langle {\hat{{{{{{{{\bf{j}}}}}}}}}}_{0}^{{{{\dagger}}} }\cdot {\hat{{{{{{{{\bf{j}}}}}}}}}}_{0}\rangle }_{{{{{{{{\rm{th}}}}}}}}}\to {\varepsilon }^{{\prime\prime} }(\omega )$, it follows that the field correlation in a given medium is proportional to ∣*ε*(*ω*)∣^−1^. This explains why thermal fields excited by fluctuating currents within an ENZ body are specially strong in the limit of *ε*(*ω*) → 0^73^. However, due to the extreme boundary conditions of ENZ media^74^, these fluctuations are trapped within the source’s hosting, and no particular resonance in the zeroth-order spectrum is found at the ENZ frequency, neither in the far nor in the near-field regimes. Again, the trapping effect of the boundary is mathematically reflected into the whole Green’s function (now including the Fresnel’s coefficients), which, in this case, i.e., for transitions between different media, adopts a form such that ∣**G**(**r**, ***ρ***, *ω*)∣^2^ → ∣*ε*^*″*^(*ω*)∣^0^ for *ε*(*ω*) → 0. Hence, when accounting also for the source’s contribution, it results that the field correlations vanishes, thereby inhibiting the releasing of thermal radiation^75^. By contrast, when the substrate is time-modulated, the strong ENZ field fluctuations generate secondary currents at *ω*_ENZ_ + Ω and *ω*_ENZ_ − Ω. For *ω*_ENZ_ + Ω, the interface with the substrate no longer traps propagating thermal fields, and they can be observed as far-field radiation.

#### Overcoming the black-body spectrum

As shown in Fig. 5, an important property of the ENZ-induced emission peak is that it allows the far-field emission spectrum of SiC at finite temperature (i.e., at T = 300 K) to exceed the black-body spectrum (dashed black curve). This feature, oftentimes referred to as “*super-Planckian*” thermal emission, constitutes a major breakthrough in the field of thermal emission engineering, since the black-body spectrum represents the upper limit for the far-field thermal emission. Various previous works, both theoretical^17,18^ and experimental^19^, have claimed to demonstrate this feature. However, even though the analysis is performed on the far-field emission regime, its occurrence is mistakenly justified by the use of subwavelength (or wavelength-scale) emitters, thus circumventing the applicability domain of both Planck’s and Kirchhoff’s radiation laws as far as the size of the emitters is concerned^76^. Indeed, due to the resonant response of subwavelength emitters, the absorption cross-section can exceed the geometrical cross-section, resulting in an enhancement of the radiative heat transfer that appears to be “super-Planckian”. In our case, though, the exceedance of the black-body spectrum is underpinned by the non-equilibrium condition, precisely driven by the time-modulation. Then, in contrast with thermal emitters based on spatial engineering whose far-field response is, at any event, confined within the black body, the temporal modulation, besides enabling a mechanism to dynamically control the spectral features, allows for emission overcoming the black-body spectrum.

#### Dynamical quantum vacuum amplification effects

Besides thermally fluctuating EM currents and fields, a salient quality of our quantum formalism relies on the possibility to rigorously deal with zero-point quantum vacuum fluctuations, and, accordingly, to predict the emergence of dynamical quantum vacuum amplification effects^41–44^. Indeed, modulating temporally the properties of the medium leads the higher-order EM fluctuating currents to produce both normally and anti-normally ordered correlations, thereby giving rise to squeezing transformation of the polaritonic operators, which ultimately enables the photon production from quantum vacuum states^61^. To clearly illustrate this effect, in Fig. 5 we also plot the emission spectra at T → 0 K. As expected, the only contribution to the fluctuating EM currents in the absolute zero of temperature is due to the quantum vacuum fluctuations, and then, just the second-order (time-modulated) term yields non-null contribution to the emission spectra. Noteworthily, the results show an almost exact overlapping between the spectra at T → 0 K with the time-modulated contribution of the thermal emission spectra at T = 300 K, meaning that, even at finite temperature, the dominant contribution to the time-modulation is almost exclusively associated to the quantum vacuum fluctuations, and so, to the occurrence of dynamical vacuum amplification effects. In accordance to the aforementioned narrowband ENZ-induced emission peak producing the “super-Planckian” emission released by the temporal modulation, this implies that its nature cannot really be considered to be thermal^76^, but it should actually be attributed to the existence of quantum vacuum effects. At the same time, looking into the emission spectra in the intermediate and the near-field regimes, this analysis reveals the dominant thermal character of the resonant SPhP-mediated thermal emission peak. Hence, apart from showing the potential of our formalism to tackle on dynamical vacuum amplification effects at a finite temperature, this analysis provides with valuable insights about the fundamental role of quantum vacuum fluctuations as well as the underlying quantum nature of the time-modulated radiative emission effects.

#### Far-to-near-field persistence of the ENZ-induced emission peaks released by the time-modulation

Somehow related to the above feature, we finally highlight the persistence of the ENZ-induced emission peaks at all the regimes. Indeed, from the expression of the Green’s function it can be seen that the EM fields within an ENZ body are enhanced for all *k*_*R*_ wavenumbers. Therefore, when these fields are released via time-modulation, the emission of radiation is continuously enhanced from near to far-field regimes. Consequently, the emission peak at *ω*_ENZ_ + Ω continuously exists in the near-field, intermediate-field, and far-field spectra. Likewise, an additional peak appears at *ω*_ENZ_ − Ω for the intermediate-field and near field-spectra, since evanescent fields are allowed at that frequency. This significant persistence of the ENZ-induced emission peaks released via time-modulation would allow for a resonant radiative energy transfer from near to far-field, suggesting an alternative ENZ-based thermal extraction scheme^30^.

Furthermore, it is also insightful to visualize this emission effect as the dual of a spatial grating^29^. For a spatial grating, a nanostructure fixes a given transversal wavenumber *k*_*R*_. Then, thermal emission is observed for a continuum of frequencies supporting this wavenumber, typically scanning the direction of emission. On the contrary, the time-modulated system fixes a frequency of emission, and then narrowband thermal radiation is observed in a continuum of wavenumbers, from near to far-fields. Thus, it represents a qualitatively different approach for engineering thermal emission.

### Practical feasibility, challenges, and applications

Our theoretical model offers a well-balanced approach that combines a relative mathematical simplicity with physical generality. In this sense, besides a fundamental understanding of thermal emission in time-modulated media, our formalism provides with a valuable overview to realistically address its practical implementation and observation in arbitrarily designed systems^77,78^. In doing this, a key attribute of our formalism lies on its fully analytical character, making it applicable to any configuration, including other background materials, geometries, or time-modulation profiles.

We have used a sinusoidal modulation profile, $\Delta \tilde{\chi }(t)={\varepsilon }_{0}\delta \chi \sin \Omega t$, which is a popular choice for photonic time crystals (PTCs)^79^, light amplification schemes^59^, space-time metamaterials^60^, optical isolators^80^, and the dynamical Casimir effect^43^, just to name a few. Such a model is basically characterized by the strength, *δχ*, and the frequency, Ω, of the modulation, which mark its practical feasibility. In our numerical examples, we have focused on specific values *δχ* = 0.025 and Ω = 1.5 THz, corresponding to Ω/*ω*_ENZ_ ≈ 0.05, strong enough to clearly illustrate the physics of the system. However, such values of *δχ* and Ω are not essential for the discussed results to happen [see Supplementary Information Figs. S1 and S2], they simply adjust and strengthen the proposed effects. Therefore, the design of an experimental setup might include trade-offs with the values of *δχ* and Ω. For example, since *δχ* and Ω basically control the strength of the effect, their values may also be relaxed by a higher detector sensitivity or temperature of operation.

As a material platform we have used SiC due to its historical and technological interest for thermal emission^29,32–34^. Recently, the time modulation of the dielectric permittivity of SiC has been experimentally demonstrated within the context of the parametric amplification of optical phonons^81^ and the active tuning of localized SPhPs resonances^82^. In particular, the experiment carried out in ref. ^82^ provides measurements of ultra-fast reflectivity changes from 5% to 25% for lifetimes ranging from 20 ps to 50 ps. While a lifetime of 20 ps corresponds to a frequency ratio of Ω/*ω*_ENZ_ ≈ 0.002 at the ENZ point of SiC, further advances in the time-modulation of SiC permittivity and the proper experimental design might make the predicted effects observable. For example, as reported in^82^, those lifetimes are inversely proportional to the pulse energy, allowing for modulation depth/frequency trade-offs.

Moreover, recent investigations towards the experimental demonstration of PTCs have focused on transparent conducting oxides (TCOs) operating at the ENZ regime, which are being consolidated as one of the most promising material platforms for time-varying photonics^83,84^. TCOs encompass doped semiconductors such as Indium Tin Oxide (ITO) or Aluminum Zinc Oxide (AZO), whose material parameters can be dynamically tuned both electrically and optically^85,86^. Focusing on all-optical schemes, the most common methods to perform time-modulation are based either on photocarrier injection/depletion or the exploitation of nonlinear optical effects^3,78,84^. Alongside this high-performance of TCOs in terms of the speed of modulation, a particularly strong nonlinear response is observed at the ENZ wavelength^87^. Recent experiments have shown sub-picosecond^88^, tenths of fs^89^, and even few-fs^90^ times. 100 fs and 10 fs periods at an ENZ wavelength of 1300 nm would correspond to frequency ratios of Ω/*ω*_ENZ_ ≈ 0.04 and Ω/*ω*_ENZ_ ≈ 0.4, respectively. Thus, these recent experiments suggest that the predicted effects could be observable with state-of-the-art TCOs nonlinearities.

While the development of incandescent temporal metamaterials is still challenging, time-modulation poses very promising prospects for applications and functionalities in thermal emission engineering. First, introducing the temporal degree freedom enables narrowband thermal emitters circumventing the need of complex nanofabrication processes, and thus providing higher flexibility and reconfigurability capabilities. Time-varying emission also directly aligns with dynamical thermal emitters, which are required for heat management and harvesting, radiative cooling, thermoregulation, camouflaging, and imaging. Moreover, the possibility of overcoming the black body spectrum and actively boosting thermal emission suggests applications as photonic heat engines. Furthermore, the range of effects covered by incandescent temporal metamaterials includes quantum vacuum amplifications effects at finite temperature, of fundamental relevance for testing quantum theories and developing non-classical light sources. Finally, it was found that time-modulation enables the release and observation of strong fluctuating thermal fields confined within an ENZ body. In doing so, time-modulation allows for the observation of material responses with otherwise no clear spectroscopic signature, thus posing very interesting perspectives for material science.

In summary, we have elaborated a quantum theoretical formalism to address thermal emission processes in time-modulated materials. Consequentially, we have also conducted the corresponding extension of the FDT. Upon this basis, we have demonstrated the emergence of physical properties associated to time-varying media, such as non-local correlations, and the stretching out of far-field thermal radiation beyond the black-body spectrum. Besides facilitating the control of thermal fluctuations, these properties increase the coherence of thermal fields, and, more importantly, give rise to unique wave phenomena. Specifically, we have highlighted the role of the ENZ media as a genuine platform to release internal field fluctuations trapped within the boundary of a material. Further, we have underscored the permanence of such an ENZ-induced emission peak also in the near-field regime, representing an innovative thermal emitter, dual to spatial gratings. While we have focused on pure time-varying media, the present approach could be extended to spatiotemporal metamaterials, likely uncovering additional thermal emission effects.

Finally, it should be noted that, although we have mainly looked into the application of the FDT on thermal emission processes, the scope of such a fundamental theorem, and hence its extension to time-varying media, is completely general, and it may concern to other non-classical phenomena, for example, non-contact friction forces, the dynamical Casimir effects, or other exciting mechanisms to amplify the quantum vacuum fluctuations. In this respect, we expect that our theoretical analysis may promote the further exploration of these features, unraveling other phenomena and unforeseen applications, as well as fostering the search of venues to carry out its experimental realization.

## Methods

### Evaluating the dyadic Green’s functions

One of the major technical difficulties in evaluating thermal emission from a time-modulated system lies in computing the product of multiple dyadic Green’s functions, which represent the interaction between the fluctuating EM currents and the radiated fields. Due to the translational symmetry of the proposed system [see Fig. 3], one can take advantage of an angular spectrum representation^35,56^, whereby the dyadic Green’s function is expressed as a superposition of plane waves: ${{{{{{{\bf{G}}}}}}}}({{{{{{{\bf{r}}}}}}}},\, {{{{{{{\boldsymbol{\rho }}}}}}}},\, \omega )=\frac{{k}_{0}^{2}}{4{\pi }^{2}}{\iint }_{{{{{{{{\mathcal{V}}}}}}}}}{d}^{2}{{{{{{{{\boldsymbol{\kappa }}}}}}}}}_{\parallel }\hat{{{{{{{{\bf{G}}}}}}}}}({\kappa }_{x},\, {\kappa }_{y};\omega|z,\, {\rho }_{z}){e}^{i{k}_{0}{{{{{{{{\boldsymbol{\kappa }}}}}}}}}_{\parallel }{{{{{{{{\boldsymbol{\rho }}}}}}}}}_{\parallel }}$, where *k*_0_ = *ω*/*c*, ***κ***_∥_ = (*κ*_*x*_, *κ*_*y*_, 0), and ***ρ***_∥_ = (*ρ*_*x*_, *ρ*_*y*_, 0). This formalism provides with valuable physical intuition by separating the modes into propagating and evanescent, from the character of their wavevector. Indeed, for each half-space, ${{{{{{{{\bf{k}}}}}}}}}_{i}={k}_{0}({\kappa }_{x},\, {\kappa }_{y},\, \sqrt{{\varepsilon }_{i}(\omega )}{\kappa }_{z,\, i})$, so ${k}_{z,\, i}={k}_{0}{\tilde{k}}_{z,\, i}={k}_{0}\sqrt{{\varepsilon }_{i}(\omega )}{\kappa }_{z,\, i}$, where ${\kappa }_{z,\, i}=\sqrt{1-{\kappa }_{R}^{2}/{\varepsilon }_{i}(\omega )}$, and ${\kappa }_{R}^{2}={\kappa }_{x}^{2}+{\kappa }_{y}^{2}$^34^. Thus, for lossless media (i.e., those where *ε*_*i*_(*ω*) is real), it is possible to set the usual correspondence of ${\kappa }_{R}^{2}\, \le \, {\varepsilon }_{i}$ and ${\kappa }_{R}^{2} \, > \, {\varepsilon }_{i}$, with propagating and evanescent modes.

Next, it should be noted that the currents and fields, linked by the Green’s functions, are in general at different locations, which may be placed either in different half-spaces, or both in the same medium (cf. refs. ^34,35,56,91^). In the former case, the specific form of the Green’s tensor relating currents in the lower half-space (made of a dispersive material, labeled as medium 2, with permittivity *ε*_2_ = *ε*(*ω*) and coordinates *ρ*_*z*_ ≤ 0) to fields in the upper half-space (being the vacuum, labeled as medium 1, with *ε*_1_ = 1 and coordinates *z* > 0), reads as

19 $${\hat{{{{{{{{\bf{G}}}}}}}}}}_{1\leftarrow 2}({{{{{{{\bf{k}}}}}}}};\omega|z,\, {\rho }_{z})=\frac{i}{2{k}_{z,\, 2}}\left[{t}_{1\leftarrow 2}^{({{{{{{{\rm{s}}}}}}}})}{\hat{{{{{{{{\bf{t}}}}}}}}}}_{1\leftarrow 2}^{({{{{{{{\rm{s}}}}}}}})}+{t}_{1\leftarrow 2}^{({{{{{{{\rm{p}}}}}}}})}{\hat{{{{{{{{\bf{t}}}}}}}}}}_{1\leftarrow 2}^{({{{{{{{\rm{p}}}}}}}})}\right]{\Gamma }_{1\leftarrow 2},\,$$

where ${\hat{{{{{{{{\bf{t}}}}}}}}}}_{1\leftarrow 2}^{({{{{{{{\rm{s}}}}}}}})}=\hat{{{{{{{{\bf{s}}}}}}}}}\otimes \hat{{{{{{{{\bf{s}}}}}}}}}$ and ${\hat{{{{{{{{\bf{t}}}}}}}}}}_{1\leftarrow 2}^{({{{{{{{\rm{p}}}}}}}})}={\hat{{{{{{{{\bf{p}}}}}}}}}}_{1}^{+}\otimes {\hat{{{{{{{{\bf{p}}}}}}}}}}_{2}^{+}$ stand for the dyadic product of the normalized polarization-vector basis, being $\hat{{{{{{{{\bf{s}}}}}}}}}\equiv (\sin {\kappa }_{\varphi },\, -\cos {\kappa }_{\varphi },\, 0)$ and ${\hat{{{{{{{{\bf{p}}}}}}}}}}_{i}^{\pm }\equiv (-{\kappa }_{z,\, i}\cos {\kappa }_{\varphi },\, -{\kappa }_{z,\, i}\sin {\kappa }_{\varphi },\, \pm {\kappa }_{R}/\sqrt{{\varepsilon }_{i}})$, ${t}_{1\leftarrow 2}^{({{{{{{{\rm{s}}}}}}}}/{{{{{{{\rm{p}}}}}}}})}$ are the corresponding Fresnel transmission coefficients associated to the *s* and *p* polarizations^35^, and ${\Gamma }_{1\leftarrow 2}={e}^{i({k}_{z,\, 1}z-{k}_{z,\, 2}{\rho }_{z})}$ is the field propagator for this particular case [see Supplementary Information Section VI]. On the other hand, in the case of two points lying in the lower medium 2, it follows that

20 $${\hat{{{{{{{{\bf{G}}}}}}}}}}_{2\leftarrow {2}^{{\prime} }}({{{{{{{\bf{k}}}}}}}};\omega|{\rho }_{z},\, {\rho }_{z}^{{\prime} })={\hat{{{{{{{{\bf{R}}}}}}}}}}_{2\leftarrow {2}^{{\prime} }}+{\hat{{{{{{{{\bf{T}}}}}}}}}}_{2\leftarrow {2}^{{\prime} }}+{\hat{{{{{{{{\bf{Z}}}}}}}}}}_{2\leftarrow {2}^{{\prime} }},\,$$

with,

21a $${\hat{{{{{{{{\bf{R}}}}}}}}}}_{2\leftarrow {2}^{{\prime} }}=\frac{i}{2{k}_{z,\, 2}}\left[{r}_{2\leftarrow 1}^{({{{{{{{\rm{s}}}}}}}})}{\hat{{{{{{{{\bf{r}}}}}}}}}}_{2\leftarrow {2}^{{\prime} }}^{({{{{{{{\rm{s}}}}}}}})}+{r}_{2\leftarrow 1}^{({{{{{{{\rm{p}}}}}}}})}{\hat{{{{{{{{\bf{r}}}}}}}}}}_{2\leftarrow {2}^{{\prime} }}^{({{{{{{{\rm{p}}}}}}}})}\right]{e}^{-i{k}_{z,\, 2}({\rho }_{z}+{\rho }_{z}^{{\prime} })},\,$$

21b $${\hat{{{{{{{{\bf{T}}}}}}}}}}_{2\leftarrow {2}^{{\prime} }}=\frac{i}{2{k}_{z,\, 2}}\left[{\hat{{{{{{{{\bf{t}}}}}}}}}}_{2\leftarrow {2}^{{\prime} }}^{({{{{{{{\rm{s}}}}}}}})}+{\hat{{{{{{{{\bf{t}}}}}}}}}}_{2\leftarrow {2}^{{\prime} }}^{({{{{{{{\rm{p}}}}}}}})}\right]{e}^{-i{k}_{z,\, 2}|{\rho }_{z}-{\rho }_{z}^{{\prime} }|},\,$$

21c $${\hat{{{{{{{{\bf{Z}}}}}}}}}}_{2\leftarrow {2}^{{\prime} }}=-\frac{1}{{k}_{0}^{2}{\varepsilon }_{2}}[\hat{{{{{{{{\bf{z}}}}}}}}}\otimes \hat{{{{{{{{\bf{z}}}}}}}}}]\delta [{\rho }_{z}-{\rho }_{z}^{{\prime} }],\,$$

where ${\hat{{{{{{{{\bf{r}}}}}}}}}}_{2\leftarrow {2}^{{\prime} }}^{({{{{{{{\rm{s}}}}}}}})}={\hat{{{{{{{{\bf{t}}}}}}}}}}_{2\leftarrow {2}^{{\prime} }}^{({{{{{{{\rm{s}}}}}}}})}=\hat{{{{{{{{\bf{s}}}}}}}}}\otimes \hat{{{{{{{{\bf{s}}}}}}}}}$, ${\hat{{{{{{{{\bf{r}}}}}}}}}}_{2\leftarrow {2}^{{\prime} }}^{({{{{{{{\rm{p}}}}}}}})}={\hat{{{{{{{{\bf{p}}}}}}}}}}_{2}^{-}\otimes {\hat{{{{{{{{\bf{p}}}}}}}}}}_{2}^{+}$, ${\hat{{{{{{{{\bf{t}}}}}}}}}}_{2\leftarrow {2}^{{\prime} }}^{({{{{{{{\rm{p}}}}}}}})}={\hat{{{{{{{{\bf{p}}}}}}}}}}_{2}^{\mp }\otimes {\hat{{{{{{{{\bf{p}}}}}}}}}}_{2}^{\pm }$, with the signs + and − being properly arranged according to the terms appearing in the absolute value of the exponential characterizing the field propagation [see Supplementary Information Section VI], and ${r}_{2\leftarrow 1}^{({{{{{{{\rm{s}}}}}}}}/{{{{{{{\rm{p}}}}}}}})}$ are the Fresnel reflection coefficients^35^. These terms, respectively characterizing the possible partial processes of reflection, transmission, or self-interaction^34,91^, allow to account for all the possible scenarios, and so, to properly determine the correlations between higher-order fluctuating EM currents [see Fig. 4].

These are all the ingredients needed to calculate the thermal emission spectra of a time-modulated semi-infinite dielectric planar slab. Notice that, despite the geometrical simplicity of the system, complexity arises as a consequence of the need to consider the interactions inside the medium, which shall lead to the occurrence of nontrivial correlations between fluctuating EM currents.

## Supplementary information

Supplementary Information

## Data Availability

Data used in this study are available from the corresponding author upon request.

## Code availability

Code used in this study are available from the corresponding author upon request.
