# Supplementary material for: Incandescent temporal metamaterials
Source: Nat Commun. 2023 Aug 1;14:4606. doi: 10.1038/s41467-023-40281-2 (PMC10394077; doi:10.1038/s41467-023-40281-2)
Supplement: Supplementary file 1 — Supplementary Information [file 41467_2023_40281_MOESM1_ESM.pdf]

Supplementary Information: Incandescent temporal metamaterials

J. Enrique Vázquez-Lozano* and Iñigo Liberal†
Department of Electrical, Electronic and Communications Engineering, Institute of Smart Cities (ISC), Universidad Pública de Navarra (UPNA), 31006 Pamplona, Spain

This *Supplementary Information* provides a step-by-step derivation of the fluctuation-dissipation relations for time-varying quantum systems and brings out its practical application by analyzing a particular case of thermal emission. Specifically, we first elaborate on the equations of motion in the *Heisenberg picture*, that naturally leads to the dynamic of the operators, and thus, to that of the fields, from which we derive a closed and analytical (though iterative) expression for the fluctuating electric fields and currents. Then, just by evaluating their corresponding correlations, we find a connection to their dissipation features, thus putting forward a new form of the *fluctuation-dissipation theorem for time-varying quantum systems*. Finally, we apply this formalism to the calculation of the thermal emission spectra, considering, as a particular case, a semi-infinite planar slab whose time-varying susceptibility is subjected to a harmonic modulation.

I. HAMILTONIAN OF A TIME-VARYING QUANTUM SYSTEM: A MACROSCOPIC QED APPROACH

We start by considering a time-varying quantum system whose dynamical behavior can be simply described by means of a perturbative Hamiltonian consisting in the sum of two contributions:

$$\hat{\mathcal{H}} = \hat{\mathcal{H}}_0 + \hat{\mathcal{H}}_{\mathrm{T}}, \tag{S.I.1}$$

where

$$\hat{\mathcal{H}}_0 \equiv \int d^3\mathbf{r} \int_0^{+\infty} d\omega_f \hbar\omega_f \hat{\mathbf{f}}^\dagger(\mathbf{r},\omega_f;t)\cdot\hat{\mathbf{f}}(\mathbf{r},\omega_f;t), \tag{S.I.1a}$$

$$\hat{\mathcal{H}}_{\mathrm{T}} \equiv -\int d^3\mathbf{r}\hat{\boldsymbol{\mathcal{P}}}(\mathbf{r},t)\cdot\hat{\boldsymbol{\mathcal{E}}}(\mathbf{r},t) = -\int d^3\mathbf{r}\left[\int_0^t d\tau\Delta\chi(\mathbf{r},t,\tau)\hat{\boldsymbol{\mathcal{E}}}(\mathbf{r},\tau)\right]\cdot\hat{\boldsymbol{\mathcal{E}}}(\mathbf{r},t), \tag{S.I.1b}$$

characterize, respectively, the photonic environment and the interaction term, this latter yielded by the polarization field induced by an external time-modulation [1, 2]. Notice that, for clarity, we are adopting a notation showing explicitly the time dependence of the (creation and annihilation) polaritonic operators ($\mathbf{f}^\dagger$ and $\mathbf{f}$) [3–6]. Furthermore, it is worth noticing the mathematical character of the electric and polarization vector fields, which, within the context of *macroscopic quantum electrodynamics* (MQED) [6] are not functions, but quantum operators (indicated with a hat), thus bearing well known properties such as their way to be applied over quantum states, or their, in general, non-commutative character. In this regard it should be noted that the electric field operator generally reads as:

$$\hat{\boldsymbol{\mathcal{E}}}(\mathbf{r};t) = \hat{\boldsymbol{\mathcal{E}}}^{(+)}(\mathbf{r};t) + \hat{\boldsymbol{\mathcal{E}}}^{(-)}(\mathbf{r};t), \tag{S.I.2}$$

with

$$\hat{\boldsymbol{\mathcal{E}}}^{(+)}(\mathbf{r};t) = \int_{-\infty}^{+\infty} d^3\boldsymbol{\rho}\int_0^{+\infty} d\omega_f \mathbf{G}_{\mathrm{E}}(\mathbf{r},\boldsymbol{\rho},\omega_f)\hat{\mathbf{f}}(\boldsymbol{\rho},\omega_f;t), \tag{S.I.2a}$$

$$\hat{\boldsymbol{\mathcal{E}}}^{(-)}(\mathbf{r};t) = \int_{-\infty}^{+\infty} d^3\boldsymbol{\rho}\int_0^{+\infty} d\omega_f \mathbf{G}^*_{\mathrm{E}}(\mathbf{r},\boldsymbol{\rho},\omega_f)\hat{\mathbf{f}}^\dagger(\boldsymbol{\rho},\omega_f;t), \tag{S.I.2b}$$

being the positive- and negative-frequency components, and

$$\mathbf{G}_{\mathrm{E}}(\mathbf{r},\boldsymbol{\rho},\omega_f) \equiv i\sqrt{\frac{\hbar}{\pi\varepsilon_0}}\left(\frac{\omega_f}{c}\right)^2\sqrt{\mathrm{Im}[\varepsilon(\boldsymbol{\rho},\omega_f)]}\mathbf{G}(\mathbf{r},\boldsymbol{\rho},\omega_f), \tag{S.I.3}$$

the response function, depending in turn on the *dyadic Green's function*, $\mathbf{G}(\mathbf{r},\boldsymbol{\rho},\omega_f)$, characterizing the medium. Therefore, the non-commutativity of the electric field operator is underpinned by the *equal-time commutation relations* of the polaritonic operators [1, 3, 6]:

$$\left[\hat{\mathbf{f}}(\mathbf{r},\omega_f;t),\hat{\mathbf{f}}(\mathbf{r}',\omega'_f;t)\right] = \left[\hat{\mathbf{f}}^\dagger(\mathbf{r},\omega_f;t),\hat{\mathbf{f}}^\dagger(\mathbf{r}',\omega'_f;t)\right] = 0, \tag{S.I.4a}$$

$$\left[\hat{\mathbf{f}}(\mathbf{r},\omega_f;t),\hat{\mathbf{f}}^\dagger(\mathbf{r}',\omega'_f;t)\right] = \mathbb{I}\delta[\mathbf{r}-\mathbf{r}']\delta\left[\omega_f-\omega'_f\right]. \tag{S.I.4b}$$

II. HEISENBERG EQUATIONS OF MOTION OF THE POLARITONIC OPERATORS

As it has already been anticipated (by emphasizing the time dependence into the operators), throughout this work the analysis shall be carried out within the *Heisenberg picture*. It should be noted that introducing the time dependence into the states (i.e., adopting the *Schrödinger picture*) is not an easy task at all, since it would require for additional knowledge (or sharp assumptions) as for the dynamical regime of the system. On the contrary, under the Heisenberg picture, the dynamical behavior of the (operators characterizing the) system directly stems from the *Heisenberg equation of motion*:

$$i\hbar\partial_t\hat{\mathcal{O}} \equiv \left[\hat{\mathcal{O}}, \hat{\mathcal{H}}\right]. \tag{S.II.1}$$

Considering separately the two contributions of the Hamiltonian given in Eq. (S.I.1), it follows that:

$$i\hbar\partial_t\hat{\mathbf{f}}(\mathbf{r},\omega_f;t) = \underbrace{i\hbar\partial_t\hat{\mathbf{f}}_0(\mathbf{r},\omega_f;t)}_{\text{free-evolving}} + \underbrace{i\hbar\partial_t\hat{\mathbf{f}}_\mathrm{T}(\mathbf{r},\omega_f;t)}_{\text{time-modulated}} = \left[\hat{\mathbf{f}}(\mathbf{r},\omega_f;t), \hat{\mathcal{H}}\right], \tag{S.II.2}$$

where

$$\begin{aligned}
&\left[\hat{\mathbf{f}}(\mathbf{r},\omega_f;t), \hat{\mathcal{H}}_0\right] = \hat{\mathbf{f}}(\mathbf{r},\omega_f;t)\hat{\mathcal{H}}_0 - \hat{\mathcal{H}}_0\hat{\mathbf{f}}(\mathbf{r},\omega_f;t)\\
&= \hat{\mathbf{f}}(\mathbf{r},\omega_f;t)\left\{\int d^3\mathbf{r}'\int_0^{+\infty} d\omega_f'\hbar\omega_f'\hat{\mathbf{f}}^\dagger(\mathbf{r}',\omega_f';t)\hat{\mathbf{f}}(\mathbf{r}',\omega_f';t)\right\} - \left\{\int d^3\mathbf{r}'\int_0^{+\infty} d\omega_f'\hbar\omega_f'\hat{\mathbf{f}}^\dagger(\mathbf{r}',\omega_f';t)\hat{\mathbf{f}}(\mathbf{r}',\omega_f';t)\right\}\hat{\mathbf{f}}(\mathbf{r},\omega_f;t)\\
&= \int d^3\mathbf{r}'\int_0^{+\infty} d\omega_f'\hbar\omega_f'\left[\hat{\mathbf{f}}(\mathbf{r},\omega_f;t)\hat{\mathbf{f}}^\dagger(\mathbf{r}',\omega_f';t)\right]\hat{\mathbf{f}}(\mathbf{r}',\omega_f';t) - \int d^3\mathbf{r}'\int_0^{+\infty} d\omega_f'\hbar\omega_f'\left[\hat{\mathbf{f}}^\dagger(\mathbf{r}',\omega_f';t)\hat{\mathbf{f}}(\mathbf{r}',\omega_f';t)\right]\hat{\mathbf{f}}(\mathbf{r},\omega_f;t)\\
&= \hbar\omega_f\hat{\mathbf{f}}(\mathbf{r},\omega_f;t) + \int d^3\mathbf{r}'\int_0^{+\infty} d\omega_f'\hbar\omega_f'\left[\hat{\mathbf{f}}^\dagger(\mathbf{r}',\omega_f';t)\hat{\mathbf{f}}(\mathbf{r}',\omega_f';t)\right]\hat{\mathbf{f}}(\mathbf{r},\omega_f;t) - \int d^3\mathbf{r}'\int_0^{+\infty} d\omega_f'\hbar\omega_f'\left[\hat{\mathbf{f}}^\dagger(\mathbf{r}',\omega_f';t)\hat{\mathbf{f}}(\mathbf{r}',\omega_f';t)\right]\hat{\mathbf{f}}(\mathbf{r},\omega_f;t),
\end{aligned}$$

thus leading to:

$$\left[\hat{\mathbf{f}}(\mathbf{r},\omega_f;t), \hat{\mathcal{H}}_0\right] = \hat{\mathbf{f}}(\mathbf{r},\omega_f;t)\hat{\mathcal{H}}_0 - \hat{\mathcal{H}}_0\hat{\mathbf{f}}(\mathbf{r},\omega_f;t) = \hbar\omega_f\hat{\mathbf{f}}(\mathbf{r},\omega_f;t). \tag{S.II.3}$$

Proceeding similarly with the creation operator it follows that:

$$\left[\hat{\mathbf{f}}^\dagger(\mathbf{r},\omega_f;t), \hat{\mathcal{H}}_0\right] = \hat{\mathbf{f}}^\dagger(\mathbf{r},\omega_f;t)\hat{\mathcal{H}}_0 - \hat{\mathcal{H}}_0\hat{\mathbf{f}}^\dagger(\mathbf{r},\omega_f;t) = -\hbar\omega_f\hat{\mathbf{f}}^\dagger(\mathbf{r},\omega_f;t). \tag{S.II.4}$$

So, in absence of time-modulation, the corresponding Heisenberg equations of motion for the polaritonic operators are:

$$\partial_t\hat{\mathbf{f}}_0(\mathbf{r},\omega_f;t) = -i\omega_f\hat{\mathbf{f}}_0(\mathbf{r},\omega_f;t), \tag{S.II.5a}$$
$$\partial_t\hat{\mathbf{f}}_0^\dagger(\mathbf{r},\omega_f;t) = i\omega_f\hat{\mathbf{f}}_0^\dagger(\mathbf{r},\omega_f;t). \tag{S.II.5b}$$

On the other side, the dynamic of the operators due to the time-modulation is given by the interaction Hamiltonian:

$$\begin{aligned}
&\left[\hat{\mathbf{f}}(\mathbf{r},\omega_f;t), \hat{\mathcal{H}}_\mathrm{T}\right] = \hat{\mathbf{f}}(\mathbf{r},\omega_f;t)\hat{\mathcal{H}}_\mathrm{T} - \hat{\mathcal{H}}_\mathrm{T}\hat{\mathbf{f}}(\mathbf{r},\omega_f;t)\\
&= \left\{\int d^3\mathbf{r}'\left[\int_0^t d\tau\Delta\chi(\mathbf{r}',t,\tau)\hat{\boldsymbol{\mathcal{E}}}(\mathbf{r}',\tau)\right]\cdot\hat{\boldsymbol{\mathcal{E}}}(\mathbf{r}',t)\right\}\hat{\mathbf{f}}(\mathbf{r},\omega_f;t) - \hat{\mathbf{f}}(\mathbf{r},\omega_f;t)\left\{\int d^3\mathbf{r}'\left[\int_0^t d\tau\Delta\chi(\mathbf{r}',t,\tau)\hat{\boldsymbol{\mathcal{E}}}(\mathbf{r}',\tau)\right]\cdot\hat{\boldsymbol{\mathcal{E}}}(\mathbf{r}',t)\right\}\\
&= \left\{\int d^3\mathbf{r}'\int_0^t d\tau\Delta\chi(\mathbf{r}',t,\tau)\hat{\boldsymbol{\mathcal{E}}}(\mathbf{r}',\tau)\cdot\hat{\boldsymbol{\mathcal{E}}}(\mathbf{r}',t)\right\}\hat{\mathbf{f}}(\mathbf{r},\omega_f;t) - \hat{\mathbf{f}}(\mathbf{r},\omega_f;t)\left\{\int d^3\mathbf{r}'\int_0^t d\tau\Delta\chi(\mathbf{r}',t,\tau)\hat{\boldsymbol{\mathcal{E}}}(\mathbf{r}',\tau)\cdot\hat{\boldsymbol{\mathcal{E}}}(\mathbf{r}',t)\right\}
\end{aligned}$$

$$= \int d^3\mathbf{r}' \int_0^t d\tau \Delta\chi(\mathbf{r}',t,\tau) \left[\int d^3\boldsymbol{\rho}' \int_0^{+\infty} d\omega_f' \left[\mathbf{G}_\mathrm{E}(\mathbf{r}',\boldsymbol{\rho}',\omega_f')\hat{\mathbf{f}}(\boldsymbol{\rho}',\omega_f';\tau) + \mathbf{G}^*_\mathrm{E}(\mathbf{r}',\boldsymbol{\rho}',\omega_f')\hat{\mathbf{f}}^\dagger(\boldsymbol{\rho}',\omega_f';\tau) \right] \right]$$
$$\times \left[\int d^3\boldsymbol{\rho}'' \int_0^{+\infty} d\omega_f'' \left[\mathbf{G}_\mathrm{E}(\mathbf{r}',\boldsymbol{\rho}'',\omega_f'')\hat{\mathbf{f}}(\boldsymbol{\rho}'',\omega_f'';t) + \mathbf{G}^*_\mathrm{E}(\mathbf{r}',\boldsymbol{\rho}'',\omega_f'')\hat{\mathbf{f}}^\dagger(\boldsymbol{\rho}'',\omega_f'';t) \right] \right] \hat{\mathbf{f}}(\mathbf{r},\omega_f;t)$$
$$- \hat{\mathbf{f}}(\mathbf{r},\omega_f;t) \int d^3\mathbf{r}' \int_0^t d\tau \Delta\chi(\mathbf{r}',t,\tau) \left[\int d^3\boldsymbol{\rho}' \int_0^{+\infty} d\omega_f' \left[\mathbf{G}_\mathrm{E}(\mathbf{r}',\boldsymbol{\rho}',\omega_f')\hat{\mathbf{f}}(\boldsymbol{\rho}',\omega_f';\tau) + \mathbf{G}^*_\mathrm{E}(\mathbf{r}',\boldsymbol{\rho}',\omega_f')\hat{\mathbf{f}}^\dagger(\boldsymbol{\rho}',\omega_f';\tau) \right] \right]$$
$$\times \left[\int d^3\boldsymbol{\rho}'' \int_0^{+\infty} d\omega_f'' \left[\mathbf{G}_\mathrm{E}(\mathbf{r}',\boldsymbol{\rho}'',\omega_f'')\hat{\mathbf{f}}(\boldsymbol{\rho}'',\omega_f'';t) + \mathbf{G}^*_\mathrm{E}(\mathbf{r}',\boldsymbol{\rho}'',\omega_f'')\hat{\mathbf{f}}^\dagger(\boldsymbol{\rho}'',\omega_f'';t) \right] \right]$$
$$= \iiint d^3\mathbf{r}' d^3\boldsymbol{\rho}' d^3\boldsymbol{\rho}'' \iint_0^{+\infty} d\omega_f' d\omega_f'' \int_0^t d\tau \Delta\chi(\mathbf{r}',t,\tau)$$
$$\Big\{ \Big[\mathbf{G}_\mathrm{E}(\mathbf{r}',\boldsymbol{\rho}',\omega_f')\mathbf{G}_\mathrm{E}(\mathbf{r}',\boldsymbol{\rho}'',\omega_f'')\hat{\mathbf{f}}(\boldsymbol{\rho}',\omega_f';\tau)\hat{\mathbf{f}}(\boldsymbol{\rho}'',\omega_f'';t)\hat{\mathbf{f}}(\mathbf{r},\omega_f;t) + \mathbf{G}^*_\mathrm{E}(\mathbf{r}',\boldsymbol{\rho}',\omega_f')\mathbf{G}^*_\mathrm{E}(\mathbf{r}',\boldsymbol{\rho}'',\omega_f'')\hat{\mathbf{f}}^\dagger(\boldsymbol{\rho}',\omega_f';\tau)\hat{\mathbf{f}}^\dagger(\boldsymbol{\rho}'',\omega_f'';t)\hat{\mathbf{f}}(\mathbf{r},\omega_f;t)$$
$$+ \mathbf{G}_\mathrm{E}(\mathbf{r}',\boldsymbol{\rho}',\omega_f')\mathbf{G}^*_\mathrm{E}(\mathbf{r}',\boldsymbol{\rho}'',\omega_f'')\hat{\mathbf{f}}(\boldsymbol{\rho}',\omega_f';\tau)\hat{\mathbf{f}}^\dagger(\boldsymbol{\rho}'',\omega_f'';t)\hat{\mathbf{f}}(\mathbf{r},\omega_f;t) + \mathbf{G}^*_\mathrm{E}(\mathbf{r}',\boldsymbol{\rho}',\omega_f')\mathbf{G}_\mathrm{E}(\mathbf{r}',\boldsymbol{\rho}'',\omega_f'')\hat{\mathbf{f}}^\dagger(\boldsymbol{\rho}',\omega_f';\tau)\hat{\mathbf{f}}(\boldsymbol{\rho}'',\omega_f'';t)\hat{\mathbf{f}}(\mathbf{r},\omega_f;t) \Big]$$
$$- \Big[\mathbf{G}_\mathrm{E}(\mathbf{r}',\boldsymbol{\rho}',\omega_f')\mathbf{G}_\mathrm{E}(\mathbf{r}',\boldsymbol{\rho}'',\omega_f'')\hat{\mathbf{f}}(\mathbf{r},\omega_f;t)\hat{\mathbf{f}}(\boldsymbol{\rho}',\omega_f';\tau)\hat{\mathbf{f}}(\boldsymbol{\rho}'',\omega_f'';t) + \mathbf{G}^*_\mathrm{E}(\mathbf{r}',\boldsymbol{\rho}',\omega_f')\mathbf{G}^*_\mathrm{E}(\mathbf{r}',\boldsymbol{\rho}'',\omega_f'')\hat{\mathbf{f}}(\mathbf{r},\omega_f;t)\hat{\mathbf{f}}^\dagger(\boldsymbol{\rho}',\omega_f';\tau)\hat{\mathbf{f}}^\dagger(\boldsymbol{\rho}'',\omega_f'';t)$$
$$+ \mathbf{G}_\mathrm{E}(\mathbf{r}',\boldsymbol{\rho}',\omega_f')\mathbf{G}^*_\mathrm{E}(\mathbf{r}',\boldsymbol{\rho}'',\omega_f'')\hat{\mathbf{f}}(\mathbf{r},\omega_f;t)\hat{\mathbf{f}}(\boldsymbol{\rho}',\omega_f';\tau)\hat{\mathbf{f}}^\dagger(\boldsymbol{\rho}'',\omega_f'';t) + \mathbf{G}^*_\mathrm{E}(\mathbf{r}',\boldsymbol{\rho}',\omega_f')\mathbf{G}_\mathrm{E}(\mathbf{r}',\boldsymbol{\rho}'',\omega_f'')\hat{\mathbf{f}}(\mathbf{r},\omega_f;t)\hat{\mathbf{f}}^\dagger(\boldsymbol{\rho}',\omega_f';\tau)\hat{\mathbf{f}}(\boldsymbol{\rho}'',\omega_f'';t) \Big] \Big\}.$$

At this point, to proceed further with the calculations avoiding the introduction of time-dependent commutation relations [3], we simplify the mathematical treatment by assuming a specific form for the time-varying susceptibility, in such a manner that it is considered as a kind of *local time-modulation*, namely, displaying the following form [7]:

$$\Delta\chi(\mathbf{r},t,\tau) \equiv \Delta\tilde{\chi}(\mathbf{r},t)\delta[t-\tau]. \tag{S.II.6}$$

Under this consideration, the above time integration is merely reduced to a simple substitution of $\tau \to t$, thus allowing the usage of the equal-time commutation relations given in Eqs. (S.I.4a) and (S.I.4b):

$$\left[\hat{\mathbf{f}}(\mathbf{r},\omega_f;t),\hat{\mathcal{H}}_\mathrm{T}\right] = \iiint d^3\mathbf{r}' d^3\boldsymbol{\rho}' d^3\boldsymbol{\rho}'' \iint_0^{+\infty} d\omega_f' d\omega_f'' \Delta\tilde{\chi}(\mathbf{r}',t)$$
$$\Big\{ \mathbf{G}_\mathrm{E}(\mathbf{r}',\boldsymbol{\rho}',\omega_f')\mathbf{G}_\mathrm{E}(\mathbf{r}',\boldsymbol{\rho}'',\omega_f'') \left[\hat{\mathbf{f}}(\boldsymbol{\rho}',\omega_f';t)\hat{\mathbf{f}}(\boldsymbol{\rho}'',\omega_f'';t)\hat{\mathbf{f}}(\mathbf{r},\omega_f;t) - \hat{\mathbf{f}}(\mathbf{r},\omega_f;t)\hat{\mathbf{f}}(\boldsymbol{\rho}',\omega_f';t)\hat{\mathbf{f}}(\boldsymbol{\rho}'',\omega_f'';t) \right]$$
$$+ \mathbf{G}^*_\mathrm{E}(\mathbf{r}',\boldsymbol{\rho}',\omega_f')\mathbf{G}^*_\mathrm{E}(\mathbf{r}',\boldsymbol{\rho}'',\omega_f'') \left[\hat{\mathbf{f}}^\dagger(\boldsymbol{\rho}',\omega_f';t)\hat{\mathbf{f}}^\dagger(\boldsymbol{\rho}'',\omega_f'';t)\hat{\mathbf{f}}(\mathbf{r},\omega_f;t) - \hat{\mathbf{f}}(\mathbf{r},\omega_f;t)\hat{\mathbf{f}}^\dagger(\boldsymbol{\rho}',\omega_f';t)\hat{\mathbf{f}}^\dagger(\boldsymbol{\rho}'',\omega_f'';t) \right]$$
$$+ \mathbf{G}_\mathrm{E}(\mathbf{r}',\boldsymbol{\rho}',\omega_f')\mathbf{G}^*_\mathrm{E}(\mathbf{r}',\boldsymbol{\rho}'',\omega_f'') \left[\hat{\mathbf{f}}(\boldsymbol{\rho}',\omega_f';t)\hat{\mathbf{f}}^\dagger(\boldsymbol{\rho}'',\omega_f'';t)\hat{\mathbf{f}}(\mathbf{r},\omega_f;t) - \hat{\mathbf{f}}(\mathbf{r},\omega_f;t)\hat{\mathbf{f}}(\boldsymbol{\rho}',\omega_f';t)\hat{\mathbf{f}}^\dagger(\boldsymbol{\rho}'',\omega_f'';t) \right]$$
$$+ \mathbf{G}^*_\mathrm{E}(\mathbf{r}',\boldsymbol{\rho}',\omega_f')\mathbf{G}_\mathrm{E}(\mathbf{r}',\boldsymbol{\rho}'',\omega_f'') \left[\hat{\mathbf{f}}^\dagger(\boldsymbol{\rho}',\omega_f';t)\hat{\mathbf{f}}(\boldsymbol{\rho}'',\omega_f'';t)\hat{\mathbf{f}}(\mathbf{r},\omega_f;t) - \hat{\mathbf{f}}(\mathbf{r},\omega_f;t)\hat{\mathbf{f}}^\dagger(\boldsymbol{\rho}',\omega_f';t)\hat{\mathbf{f}}(\boldsymbol{\rho}'',\omega_f'';t) \right] \Big\}$$
$$= - \iiint d^3\mathbf{r}' d^3\boldsymbol{\rho}' d^3\boldsymbol{\rho}'' \iint_0^{+\infty} d\omega_f' d\omega_f'' \Delta\tilde{\chi}(\mathbf{r}',t)\mathbb{I}\delta[\mathbf{r}-\boldsymbol{\rho}']\delta\left[\omega_f-\omega_f'\right] \left[\mathbf{G}^*_\mathrm{E}(\mathbf{r}',\boldsymbol{\rho}',\omega_f')\mathbf{G}^*_\mathrm{E}(\mathbf{r}',\boldsymbol{\rho}'',\omega_f'')\hat{\mathbf{f}}^\dagger(\boldsymbol{\rho}'',\omega_f'';t) \right]$$
$$- \iiint d^3\mathbf{r}' d^3\boldsymbol{\rho}' d^3\boldsymbol{\rho}'' \iint_0^{+\infty} d\omega_f' d\omega_f'' \Delta\tilde{\chi}(\mathbf{r}',t)\mathbb{I}\delta[\mathbf{r}-\boldsymbol{\rho}']\delta\left[\omega_f-\omega_f'\right] \left[\mathbf{G}^*_\mathrm{E}(\mathbf{r}',\boldsymbol{\rho}',\omega_f')\mathbf{G}_\mathrm{E}(\mathbf{r}',\boldsymbol{\rho}'',\omega_f'')\hat{\mathbf{f}}(\boldsymbol{\rho}'',\omega_f'';t) \right]$$
$$- \iiint d^3\mathbf{r}' d^3\boldsymbol{\rho}' d^3\boldsymbol{\rho}'' \iint_0^{+\infty} d\omega_f' d\omega_f'' \Delta\tilde{\chi}(\mathbf{r}',t)\mathbb{I}\delta[\mathbf{r}-\boldsymbol{\rho}'']\delta\left[\omega_f-\omega_f''\right] \left[\mathbf{G}^*_\mathrm{E}(\mathbf{r}',\boldsymbol{\rho}',\omega_f')\mathbf{G}^*_\mathrm{E}(\mathbf{r}',\boldsymbol{\rho}'',\omega_f'')\hat{\mathbf{f}}^\dagger(\boldsymbol{\rho}',\omega_f';t) \right]$$
$$- \iiint d^3\mathbf{r}' d^3\boldsymbol{\rho}' d^3\boldsymbol{\rho}'' \iint_0^{+\infty} d\omega_f' d\omega_f'' \Delta\tilde{\chi}(\mathbf{r}',t)\mathbb{I}\delta[\mathbf{r}-\boldsymbol{\rho}'']\delta\left[\omega_f-\omega_f''\right] \left[\mathbf{G}_\mathrm{E}(\mathbf{r}',\boldsymbol{\rho}',\omega_f')\mathbf{G}^*_\mathrm{E}(\mathbf{r}',\boldsymbol{\rho}'',\omega_f'')\hat{\mathbf{f}}(\boldsymbol{\rho}',\omega_f';t) \right].$$

Then, performing the integration over positions and frequencies and reordering the resulting expression leads to:

$$\left[\hat{\mathbf{f}}(\mathbf{r},\omega_f;t),\hat{\mathcal{H}}_{\mathrm{T}}\right] = -\iint d^3\mathbf{r}' d^3\boldsymbol{\rho}'' \int_0^{+\infty} d\omega_f'' \Delta\tilde{\chi}(\mathbf{r}',t)\left\{\mathbf{G}_{\mathrm{E}}^*(\mathbf{r}',\mathbf{r},\omega_f)\left[\mathbf{G}_{\mathrm{E}}(\mathbf{r}',\boldsymbol{\rho}'',\omega_f'')\hat{\mathbf{f}}(\boldsymbol{\rho}'',\omega_f'';t)+\mathbf{G}_{\mathrm{E}}^*(\mathbf{r}',\boldsymbol{\rho}'',\omega_f'')\hat{\mathbf{f}}^\dagger(\boldsymbol{\rho}'',\omega_f'';t)\right]\right\}$$

$$-\iint d^3\mathbf{r}' d^3\boldsymbol{\rho}' \int_0^{+\infty} d\omega_f' \Delta\tilde{\chi}(\mathbf{r}',t)\left\{\mathbf{G}_{\mathrm{E}}^*(\mathbf{r}',\mathbf{r},\omega_f)\left[\mathbf{G}_{\mathrm{E}}(\mathbf{r}',\boldsymbol{\rho}',\omega_f')\hat{\mathbf{f}}(\boldsymbol{\rho}',\omega_f';t)+\mathbf{G}_{\mathrm{E}}^*(\mathbf{r}',\boldsymbol{\rho}',\omega_f')\hat{\mathbf{f}}^\dagger(\boldsymbol{\rho}',\omega_f';t)\right]\right\}$$

$$= -2\iint d^3\mathbf{r}' d^3\boldsymbol{\rho}' \int_0^{+\infty} d\omega_f' \Delta\tilde{\chi}(\mathbf{r}',t)\left\{\mathbf{G}_{\mathrm{E}}^*(\mathbf{r}',\mathbf{r},\omega_f)\left[\mathbf{G}_{\mathrm{E}}(\mathbf{r}',\boldsymbol{\rho}',\omega_f')\hat{\mathbf{f}}(\boldsymbol{\rho}',\omega_f';t)+\mathbf{G}_{\mathrm{E}}^*(\mathbf{r}',\boldsymbol{\rho}',\omega_f')\hat{\mathbf{f}}^\dagger(\boldsymbol{\rho}',\omega_f';t)\right]\right\}.$$

Hence, from the expressions for the electric field operators [i.e., those given in Eqs. (S.I.2a) and (S.I.2b)], it follows that:

$$\left[\hat{\mathbf{f}}(\mathbf{r},\omega_f;t),\hat{\mathcal{H}}_{\mathrm{T}}\right] = -2\int d^3\boldsymbol{\rho}\Delta\tilde{\chi}(\boldsymbol{\rho},t)\mathbf{G}_{\mathrm{E}}^*(\boldsymbol{\rho},\mathbf{r},\omega_f)\hat{\boldsymbol{\mathcal{E}}}(\boldsymbol{\rho};t). \tag{S.II.7}$$

Proceeding similarly with the creation operator it can be shown that:

$$\left[\hat{\mathbf{f}}^\dagger(\mathbf{r},\omega_f;t),\hat{\mathcal{H}}_{\mathrm{T}}\right] = 2\int d^3\boldsymbol{\rho}\Delta\tilde{\chi}(\boldsymbol{\rho},t)\mathbf{G}_{\mathrm{E}}(\boldsymbol{\rho},\mathbf{r},\omega_f)\hat{\boldsymbol{\mathcal{E}}}(\boldsymbol{\rho};t). \tag{S.II.8}$$

Therefore, the corresponding *Heisenberg equations of motion for the time-modulated polaritonic operators* are:

$$\partial_t\hat{\mathbf{f}}_{\mathrm{T}}(\mathbf{r},\omega_f;t) = \frac{2i}{\hbar}\int d^3\boldsymbol{\rho}\Delta\tilde{\chi}(\boldsymbol{\rho},t)\mathbf{G}_{\mathrm{E}}^*(\boldsymbol{\rho},\mathbf{r},\omega_f)\hat{\boldsymbol{\mathcal{E}}}(\boldsymbol{\rho};t), \tag{S.II.9a}$$

$$\partial_t\hat{\mathbf{f}}_{\mathrm{T}}^\dagger(\mathbf{r},\omega_f;t) = \frac{-2i}{\hbar}\int d^3\boldsymbol{\rho}\Delta\tilde{\chi}(\boldsymbol{\rho},t)\mathbf{G}_{\mathrm{E}}(\boldsymbol{\rho},\mathbf{r},\omega_f)\hat{\boldsymbol{\mathcal{E}}}(\boldsymbol{\rho};t). \tag{S.II.9b}$$

From the results given in Eqs. (S.II.5a) and (S.II.5b), and Eqs. (S.II.9a) and (S.II.9b), along with the decomposition sketched out in Eq. (S.II.2), the dynamic of the operators brought about by both the free-evolving and the time-modulated contributions reads as,

$$\partial_t\hat{\mathbf{f}}(\mathbf{r},\omega_f;t) = \frac{1}{i\hbar}\left[\hat{\mathbf{f}}(\mathbf{r},\omega_f;t),\hat{\mathcal{H}}\right] = \partial_t\hat{\mathbf{f}}_0(\mathbf{r},\omega_f;t)+\partial_t\hat{\mathbf{f}}_{\mathrm{T}}(\mathbf{r},\omega_f;t)$$

$$= -i\omega_f\hat{\mathbf{f}}(\mathbf{r},\omega_f;t)+\frac{2i}{\hbar}\int d^3\boldsymbol{\rho}\Delta\tilde{\chi}(\boldsymbol{\rho},t)\mathbf{G}_{\mathrm{E}}^*(\boldsymbol{\rho},\mathbf{r},\omega_f)\hat{\boldsymbol{\mathcal{E}}}(\boldsymbol{\rho};t), \tag{S.II.10a}$$

$$\partial_t\hat{\mathbf{f}}^\dagger(\mathbf{r},\omega_f;t) = \frac{1}{i\hbar}\left[\hat{\mathbf{f}}^\dagger(\mathbf{r},\omega_f;t),\hat{\mathcal{H}}\right] = \partial_t\hat{\mathbf{f}}_0^\dagger(\mathbf{r},\omega_f;t)+\partial_t\hat{\mathbf{f}}_{\mathrm{T}}^\dagger(\mathbf{r},\omega_f;t)$$

$$= i\omega_f\hat{\mathbf{f}}^\dagger(\mathbf{r},\omega_f;t)-\frac{2i}{\hbar}\int d^3\boldsymbol{\rho}\Delta\tilde{\chi}(\boldsymbol{\rho},t)\mathbf{G}_{\mathrm{E}}(\boldsymbol{\rho},\mathbf{r},\omega_f)\hat{\boldsymbol{\mathcal{E}}}(\boldsymbol{\rho};t), \tag{S.II.10b}$$

thus yielding:

$$\hat{\mathbf{f}}(\mathbf{r},\omega_f;t) = \hat{\mathbf{f}}_0(\mathbf{r},\omega_f;t)+\hat{\mathbf{f}}_{\mathrm{T}}(\mathbf{r},\omega_f;t)$$

$$= \hat{\mathbf{f}}(\mathbf{r},\omega_f;t=0)e^{-i\omega_f t}+\frac{2i}{\hbar}e^{-i\omega_f t}\left[\int_0^t d\tau\int_{-\infty}^{+\infty} d^3\boldsymbol{\rho}e^{i\omega_f\tau}\Delta\tilde{\chi}(\boldsymbol{\rho},\tau)\mathbf{G}_{\mathrm{E}}^*(\boldsymbol{\rho},\mathbf{r},\omega_f)\hat{\boldsymbol{\mathcal{E}}}(\boldsymbol{\rho};\tau)\right], \tag{S.II.11a}$$

$$\hat{\mathbf{f}}^\dagger(\mathbf{r},\omega_f;t) = \hat{\mathbf{f}}_0^\dagger(\mathbf{r},\omega_f;t)+\hat{\mathbf{f}}_{\mathrm{T}}^\dagger(\mathbf{r},\omega_f;t)$$

$$= \hat{\mathbf{f}}^\dagger(\mathbf{r},\omega_f;t=0)e^{+i\omega_f t}-\frac{2i}{\hbar}e^{+i\omega_f t}\left[\int_0^t d\tau\int_{-\infty}^{+\infty} d^3\boldsymbol{\rho}e^{-i\omega_f\tau}\Delta\tilde{\chi}(\boldsymbol{\rho},\tau)\mathbf{G}_{\mathrm{E}}(\boldsymbol{\rho},\mathbf{r},\omega_f)\hat{\boldsymbol{\mathcal{E}}}(\boldsymbol{\rho};\tau)\right]. \tag{S.II.11b}$$

III. ELECTRIC FIELD OPERATOR: FREE-EVOLVING AND TIME-MODULATED CONTRIBUTIONS

Building on the above results, in this section we calculate the electric field operator making an explicit distinction between the free-evolving and the time-modulated terms. For the sake of completeness, as well as for convenience in the ensuing developments, we present both the real-valued fields in time domain, and the complex amplitudes in the frequency domain.

A. Free-evolving electric field operator

The free-evolving positive-frequency electric field operator in time domain can be straightforwardly obtained just by performing the corresponding substitution of the (creation/annihilation) polaritonic operators as given in Eq. (S.II.11a) (or Eq. (S.II.11b) for the negative-frequency part), into the electric field operator given in Eq. (S.I.2a) (or Eq. (S.I.2b) for the negative-frequency part):

$$\begin{aligned}\hat{\boldsymbol{\mathcal{E}}}_0^{(+)}(\mathbf{r};t) &= \int_{-\infty}^{+\infty} d^3\boldsymbol{\rho} \int_0^{+\infty} d\omega_f \mathbf{G}_{\mathrm{E}}(\mathbf{r},\boldsymbol{\rho},\omega_f)\hat{\mathbf{f}}_0(\boldsymbol{\rho},\omega_f;t) \\ &= \int_{-\infty}^{+\infty} d^3\boldsymbol{\rho} \int_0^{+\infty} d\omega_f \mathbf{G}_{\mathrm{E}}(\mathbf{r},\boldsymbol{\rho},\omega_f)\hat{\mathbf{f}}_0(\boldsymbol{\rho},\omega_f;t=0)e^{-i\omega_f t}. \end{aligned} \tag{S.III.1}$$

From the above expression, and noticing that we are only regarding the positive-frequency part of the field, the respective complex-like field operator in frequency domain can be directly obtained by means of the Laplace transform:

$$\begin{aligned}\hat{\mathbf{E}}_0^{(+)}(\mathbf{r};\omega) &= \int_0^{+\infty} dt \hat{\boldsymbol{\mathcal{E}}}_0^{(+)}(\mathbf{r};t)e^{i\omega t} \\ &= \int_0^{+\infty} dt \left[\int_{-\infty}^{+\infty} d^3\boldsymbol{\rho} \int_0^{+\infty} d\omega_f \mathbf{G}_{\mathrm{E}}(\mathbf{r},\boldsymbol{\rho},\omega_f)\hat{\mathbf{f}}(\boldsymbol{\rho},\omega_f;t=0)e^{-i\omega_f t}\right] e^{i\omega t} \\ &= \int_{-\infty}^{+\infty} d^3\boldsymbol{\rho} \int_0^{+\infty} d\omega_f \mathbf{G}_{\mathrm{E}}(\mathbf{r},\boldsymbol{\rho},\omega_f)\hat{\mathbf{f}}(\boldsymbol{\rho},\omega_f;t=0)\left[\frac{i}{\omega-\omega_f+i0^+}\right] \\ &= \int_{-\infty}^{+\infty} d^3\boldsymbol{\rho} \int_0^{+\infty} d\omega_f \mathbf{G}_{\mathrm{E}}(\mathbf{r},\boldsymbol{\rho},\omega_f)\hat{\mathbf{f}}(\boldsymbol{\rho},\omega_f;t=0)\left\{\pi\delta[\omega-\omega_f] + i\mathcal{P}\left[\frac{1}{\omega-\omega_f}\right]\right\} \\ &= \pi\int_{-\infty}^{+\infty} d^3\boldsymbol{\rho} \int_0^{+\infty} d\omega_f \delta[\omega-\omega_f]\mathbf{G}_{\mathrm{E}}(\mathbf{r},\boldsymbol{\rho},\omega_f)\hat{\mathbf{f}}(\boldsymbol{\rho},\omega_f;t=0) + i\mathcal{P}\left[\int_{-\infty}^{+\infty} d^3\boldsymbol{\rho} \int_0^{+\infty} d\omega_f \frac{\mathbf{G}_{\mathrm{E}}(\mathbf{r},\boldsymbol{\rho},\omega_f)\hat{\mathbf{f}}(\boldsymbol{\rho},\omega_f;t=0)}{\omega-\omega_f}\right] \\ &= \pi\int_{-\infty}^{+\infty} d^3\boldsymbol{\rho}\mathbf{G}_{\mathrm{E}}(\mathbf{r},\boldsymbol{\rho},\omega)\hat{\mathbf{f}}(\boldsymbol{\rho},\omega;t=0) + i\pi\frac{1}{\pi}\mathcal{P}\left[\int_{-\infty}^{+\infty} d^3\boldsymbol{\rho} \int_0^{+\infty} d\omega_f \frac{\mathbf{G}_{\mathrm{E}}(\mathbf{r},\boldsymbol{\rho},\omega_f)\hat{\mathbf{f}}(\boldsymbol{\rho},\omega_f;t=0)}{\omega-\omega_f}\right], \end{aligned} \tag{S.III.2}$$

where it has been used the identity,

$$\int_0^{+\infty} dt e^{i\Delta t} = \frac{i}{\Delta + i0^+}, \tag{S.III.3}$$

and the so-called *Sokhotski-Plemelj theorem*,

$$\lim_{\varepsilon\to 0} \frac{1}{\Delta \pm i\varepsilon^+} \to \mp i\pi\delta[\Delta] + \mathcal{P}\left[\frac{1}{\Delta}\right], \tag{S.III.4}$$

where $\mathcal{P}$ stands for the *Cauchy principal value*, thereby accounting for the poles of causal functions. Here it is worth stressing that in order to complete the Green's function, we would have to consider the extension of the lower limit of the frequency integral to $-\infty$. Such an approximation is commonly used (see, e.g., Refs. [1, 3]), and it is often claimed that leads to some missing terms resulting from the subsequent application of the *rotating wave approximation*. However, in this case it should be noted that no rotating wave approximation is employed at all, so that there are no

missing terms coming from such an assumption. Consequently, the second integral can be performed as follows:

$$\frac{1}{\pi}\mathcal{P}\left[\int_{-\infty}^{+\infty} d^3\boldsymbol{\rho}\int_0^{+\infty} d\omega_f \frac{\mathbf{G}_{\mathrm{E}}(\mathbf{r},\boldsymbol{\rho},\omega_f)\hat{\mathbf{f}}(\boldsymbol{\rho},\omega_f;t=0)}{\omega-\omega_f}\right] = \int_{-\infty}^{+\infty} d^3\boldsymbol{\rho}\frac{1}{\pi}\mathcal{P}\left[\int_0^{+\infty} d\omega_f \frac{\mathcal{G}(\mathbf{r},\boldsymbol{\rho},\omega_f)}{\omega-\omega_f}\right]$$
$$= \int_{-\infty}^{+\infty} d^3\boldsymbol{\rho}\,[\mathrm{Im}[\mathcal{G}(\mathbf{r},\boldsymbol{\rho},\omega)] - i\mathrm{Re}[\mathcal{G}(\mathbf{r},\boldsymbol{\rho},\omega)]], \tag{S.III.5}$$

where the computation of the integral has been done on account of the *Kramers-Kronig relations*,

$$\mathrm{Re}[\mathcal{G}(\mathbf{r},\boldsymbol{\rho},\omega)] = \frac{1}{\pi}\mathcal{P}\left[\int_{-\infty}^{+\infty} d\omega' \frac{\mathrm{Im}[\mathcal{G}(\mathbf{r},\boldsymbol{\rho},\omega)]}{\omega'-\omega}\right], \tag{S.III.6a}$$

$$\mathrm{Im}[\mathcal{G}(\mathbf{r},\boldsymbol{\rho},\omega)] = \frac{1}{\pi}\mathcal{P}\left[\int_{-\infty}^{+\infty} d\omega' \frac{\mathrm{Re}[\mathcal{G}(\mathbf{r},\boldsymbol{\rho},\omega)]}{\omega-\omega'}\right], \tag{S.III.6b}$$

over the function $\mathcal{G}(\mathbf{r},\boldsymbol{\rho},\omega_f) = \mathbf{G}_{\mathrm{E}}(\mathbf{r},\boldsymbol{\rho},\omega_f)\hat{\mathbf{f}}(\boldsymbol{\rho},\omega_f;t=0)$. Therefore, putting it all together, the positive-frequency part of the free-evolving electric field operator in frequency domain reads as:

$$\hat{\mathbf{E}}_0^{(+)}(\mathbf{r};\omega) = 2\pi\int_{-\infty}^{+\infty} d^3\boldsymbol{\rho}\mathbf{G}_{\mathrm{E}}(\mathbf{r},\boldsymbol{\rho},\omega)\hat{\mathbf{f}}(\boldsymbol{\rho},\omega;t=0). \tag{S.III.7}$$

B. Time-modulated electric field operator

Similarly, the time-modulated electric field operator in time domain can be simply obtained by inserting the corresponding polaritonic operator into the electric field operator. Once again, focusing only into the positive-frequency part it follows that,

$$\hat{\boldsymbol{\mathcal{E}}}_{\mathrm{T}}^{(+)}(\mathbf{r};t) = \int_{-\infty}^{+\infty} d^3\boldsymbol{\rho}\int_0^{+\infty} d\omega_f \mathbf{G}_{\mathrm{E}}(\mathbf{r},\boldsymbol{\rho},\omega_f)\hat{\mathbf{f}}_{\mathrm{T}}(\boldsymbol{\rho},\omega_f;t)$$
$$= \frac{2i}{\hbar}\int_{-\infty}^{+\infty} d^3\boldsymbol{\rho}\int_0^{+\infty} d\omega_f \mathbf{G}_{\mathrm{E}}(\mathbf{r},\boldsymbol{\rho},\omega_f)\left[e^{-i\omega_f t}\int_0^t d\tau'\int_{-\infty}^{+\infty} d^3\boldsymbol{\rho}' e^{i\omega_f\tau'}\Delta\tilde{\chi}(\boldsymbol{\rho}',\tau')\mathbf{G}_{\mathrm{E}}^*(\boldsymbol{\rho}',\boldsymbol{\rho},\omega_f)\hat{\boldsymbol{\mathcal{E}}}(\boldsymbol{\rho}';\tau')\right]$$
$$= \int_{-\infty}^{+\infty} d^3\boldsymbol{\rho}'\int_0^t d\tau'\left\{\frac{2i}{\pi\varepsilon_0 c^2}\int_0^{+\infty} d\omega_f\omega_f^2 e^{-i\omega_f(t-\tau')}\mathrm{Im}[\mathbf{G}(\mathbf{r},\boldsymbol{\rho}',\omega_f)]\right\}\Delta\tilde{\chi}(\boldsymbol{\rho}',\tau')\hat{\boldsymbol{\mathcal{E}}}(\boldsymbol{\rho}';\tau'), \tag{S.III.8}$$

where it has been used the *completeness relation of the dyadic Green's function* [3, 6]:

$$\int_{-\infty}^{+\infty} d^3\boldsymbol{\rho}\frac{\omega^2}{c^2}\mathrm{Im}[\varepsilon(\boldsymbol{\rho},\omega)]\mathbf{G}(\mathbf{r},\boldsymbol{\rho},\omega)\mathbf{G}^*(\mathbf{r}',\boldsymbol{\rho},\omega) = \mathrm{Im}[\mathbf{G}(\mathbf{r},\mathbf{r}',\omega)], \tag{S.III.9}$$

which can be derived from the *Schwarz reflection principle*, $\mathbf{G}^*(\mathbf{r},\mathbf{r}',\omega) = \mathbf{G}(\mathbf{r},\mathbf{r}',-\omega^*)$, the *Lorentz reciprocity*, $\mathbf{G}^{\mathrm{T}}(\mathbf{r},\mathbf{r}',\omega) = \mathbf{G}(\mathbf{r}',\mathbf{r},\omega)$, requiring the condition that $\mathbf{G}(\mathbf{r},\mathbf{r}',\omega) \to 0$ at $\mathbf{r}\to\infty$ (namely, ensuring that there is no net energy transport, i.e., the time-averaged Poynting vector is to be zero for any point in space), and making use of the own definition of $\mathbf{G}(\mathbf{r},\mathbf{r}',\omega)$:

$$\nabla\times\nabla\times\mathbf{G}(\mathbf{r},\mathbf{r}',\omega) - k^2\mathbf{G}(\mathbf{r},\mathbf{r}',\omega) = \mathbb{I}\delta[\mathbf{r}-\mathbf{r}']. \tag{S.III.10}$$

In this way, the real-valued time-modulated field operator can be written as the spatial integral of a *memory kernel*, acting on the polarization field operator:

$$\hat{\boldsymbol{\mathcal{E}}}_{\mathrm{T}}^{(+)}(\mathbf{r};t) = \int_{-\infty}^{+\infty} d^3\boldsymbol{\rho}\left\{\int_0^t d\tau\boldsymbol{\mathcal{K}}_{\mathrm{E}}^{(+)}(\mathbf{r},\boldsymbol{\rho},t-\tau)\hat{\boldsymbol{\mathcal{P}}}(\boldsymbol{\rho};\tau)\right\}, \tag{S.III.11}$$

with $\hat{\boldsymbol{\mathcal{P}}}(\boldsymbol{\rho};\tau)\equiv\Delta\tilde{\chi}(\boldsymbol{\rho},\tau)\hat{\boldsymbol{\mathcal{E}}}(\boldsymbol{\rho};\tau)$, and

$$\boldsymbol{\mathcal{K}}_{\mathrm{E}}^{(+)}(\mathbf{r},\boldsymbol{\rho},t-\tau) = \frac{2i}{\pi\varepsilon_0 c^2}\int_0^{+\infty} d\omega_f\omega_f^2\mathrm{Im}[\mathbf{G}(\mathbf{r},\boldsymbol{\rho},\omega_f)]e^{-i\omega_f(t-\tau)}. \tag{S.III.12}$$

Just like the free-evolving contribution, the complex-like electric field operator in frequency domain can be calculated from the above expressions [Eqs. (S.III.11) and (S.III.12)] taking the Laplace transform:

$$\begin{aligned}
\hat{\mathbf{E}}_{\mathrm{T}}^{(+)}(\mathbf{r};\omega) &= \int_0^{+\infty} dt \hat{\boldsymbol{\mathcal{E}}}_{\mathrm{T}}^{(+)}(\mathbf{r};t)e^{i\omega t} \\
&= \int_0^{+\infty} dt \left[\int_{-\infty}^{+\infty} d^3\boldsymbol{\rho} \left\{\int_0^t d\tau \boldsymbol{\mathcal{K}}_{\mathrm{E}}^{(+)}(\mathbf{r},\boldsymbol{\rho},t-\tau)\hat{\boldsymbol{\mathcal{P}}}(\boldsymbol{\rho};\tau)\right\}\right] e^{i\omega t} \\
&= \int_{-\infty}^{+\infty} d^3\boldsymbol{\rho} \left\{\int_0^{+\infty} dt \left[\int_0^t d\tau \boldsymbol{\mathcal{K}}_{\mathrm{E}}^{(+)}(\mathbf{r},\boldsymbol{\rho},t-\tau)\Delta\tilde{\chi}(\boldsymbol{\rho},\tau)\hat{\boldsymbol{\mathcal{E}}}(\boldsymbol{\rho};\tau)\right] e^{i\omega(t-\tau)}e^{i\omega\tau}\right\} \\
&= \int_{-\infty}^{+\infty} d^3\boldsymbol{\rho} \left\{\int_0^{+\infty} d\tau \left[\int_\tau^{+\infty} dt \boldsymbol{\mathcal{K}}_{\mathrm{E}}^{(+)}(\mathbf{r},\boldsymbol{\rho},t-\tau)\Delta\tilde{\chi}(\boldsymbol{\rho},\tau)\hat{\boldsymbol{\mathcal{E}}}(\boldsymbol{\rho};\tau)\right] e^{i\omega(t-\tau)}e^{i\omega\tau}\right\} \\
&= \int_{-\infty}^{+\infty} d^3\boldsymbol{\rho} \left\{\left[\int_0^{+\infty} d\tilde{t} \boldsymbol{\mathcal{K}}_{\mathrm{E}}^{(+)}(\mathbf{r},\boldsymbol{\rho},\tilde{t})e^{i\omega\tilde{t}}\right] \cdot \left[\int_0^{+\infty} d\tau \Delta\tilde{\chi}(\boldsymbol{\rho},\tau)\hat{\boldsymbol{\mathcal{E}}}(\boldsymbol{\rho};\tau)e^{i\omega\tau}\right]\right\}, \qquad \text{(S.III.13)}
\end{aligned}$$

where it has been made the change of variable $\tilde{t} = t - \tau$. Thus, the positive-frequency part of the time-modulated electric field operator in frequency domain reads as:

$$\hat{\mathbf{E}}_{\mathrm{T}}^{(+)}(\mathbf{r};\omega) = \int_{-\infty}^{+\infty} d^3\boldsymbol{\rho} \mathbf{K}_{\mathrm{E}}^{(+)}(\mathbf{r},\boldsymbol{\rho},\omega)\mathcal{L}_\omega[\Delta\tilde{\chi}(\boldsymbol{\rho},\tau)\hat{\boldsymbol{\mathcal{E}}}(\boldsymbol{\rho};\tau)], \qquad \text{(S.III.14)}$$

where

$$\mathcal{L}_\omega[\Delta\tilde{\chi}(\boldsymbol{\rho},\tau)\hat{\boldsymbol{\mathcal{E}}}(\boldsymbol{\rho};\tau)] \equiv \int_0^{+\infty} d\tau \Delta\tilde{\chi}(\boldsymbol{\rho},\tau)\hat{\boldsymbol{\mathcal{E}}}(\boldsymbol{\rho};\tau)e^{i\omega\tau}, \qquad \text{(S.III.15)}$$

is the Laplace transform of the polarization field operator $\hat{\boldsymbol{\mathcal{P}}}(\boldsymbol{\rho};\tau) \equiv \Delta\tilde{\chi}(\boldsymbol{\rho},\tau)\hat{\boldsymbol{\mathcal{E}}}(\boldsymbol{\rho};\tau)$, and

$$\mathbf{K}_{\mathrm{E}}^{(+)}(\mathbf{r},\boldsymbol{\rho},\omega) = \frac{2\omega^2}{\varepsilon_0 c^2}\mathbf{G}(\mathbf{r},\boldsymbol{\rho},\omega). \qquad \text{(S.III.16)}$$

C. Total electric field operator

The Eq. (S.III.11), and consequently Eq. (S.III.14), are self-contained expressions in which the time-modulated positive-frequency electric field operator, $\hat{\boldsymbol{\mathcal{E}}}_{\mathrm{T}}^{(+)}$, is directly related to the total field, $\hat{\boldsymbol{\mathcal{E}}} = \hat{\boldsymbol{\mathcal{E}}}_0^{(+)} + \hat{\boldsymbol{\mathcal{E}}}_0^{(-)} + \hat{\boldsymbol{\mathcal{E}}}_{\mathrm{T}}^{(+)} + \hat{\boldsymbol{\mathcal{E}}}_{\mathrm{T}}^{(-)}$. Namely, the annihilation polaritonic operator (associated to the positive-frequency component of the electric field) shall lead to a combination of the creation and the annihilation operators, being modified (actually shifted) by the time-modulation. Furthermore, in contrast to the case of a time-invariant system (e.g., that considering a quantum single emitter), there is a spatial integral that makes the kernel and the field operator tied to each other, i.e., they appear to be tightly coupled. In turn, as a consequence of the time-modulation of the system, the susceptibility function and the field operator are also coupled through the Laplace transform. At any rate, and in view of the above results, it could be inferred that, in general, for time-varying systems is impossible to express the frequency-dependent source-like (time-modulated) electric field as an algebraic product of a kernel function times a polarization field (as it actually occurs in the stationary case). This is essentially due to the coupling that exists between the medium and the external field in this kind of time-varying systems, that translates into the fact that the Laplace transform cannot be expressed in a factorized manner. Far from being a hurdle to overcome, this is indeed a distinctive feature of time-varying systems, increasing both the richness and the variety of the achievable physical effects; in particular those involving convoluted correlations that may arise from the own time-modulation of the medium. Therefore, Eq. (S.III.14), along with Eqs. (S.III.15) and (S.III.16), is arguably the most compact form in which one may express the relationship between the time-modulated electric field operator and the total field in the frequency domain. So, in order to give a more explicit result, we shall particularize the time-dependent susceptibility $\Delta\tilde{\chi}$ to a special case, so as to be able to get an explicit expression for its associated Laplace transform.

Be that as it may, the time-modulated electric field operator, $\hat{\boldsymbol{\mathcal{E}}}_{\mathrm{T}}$, appears both in the right- and left-hand sides of the latter expression. So, at least as a first approach, it could be useful to think on a iterative procedure in which one solves order-by-order the full expression of the time-modulated electric field operator. In this way, one could then obtain, recursively, the total electric field operator in the following way:

$$\begin{aligned}\hat{\boldsymbol{\mathcal{E}}}^{(+)}(\mathbf{r};t) &= \hat{\boldsymbol{\mathcal{E}}}_0^{(+)}(\mathbf{r};t) + \hat{\boldsymbol{\mathcal{E}}}_{\mathrm{T}}^{(+)}(\mathbf{r};t)\\
&= \hat{\boldsymbol{\mathcal{E}}}_0^{(+)}(\mathbf{r};t) + \int_{-\infty}^{+\infty} d^3\boldsymbol{\rho}' \left\{ \int_0^t d\tau' \boldsymbol{\mathcal{K}}_{\mathrm{E}}^{(+)}(\mathbf{r},\boldsymbol{\rho}',t-\tau')\Delta\tilde{\chi}(\boldsymbol{\rho}',\tau')\hat{\boldsymbol{\mathcal{E}}}(\boldsymbol{\rho}';\tau') \right\}\\
&= \hat{\boldsymbol{\mathcal{E}}}_0^{(+)}(\mathbf{r};t) + \int_{-\infty}^{+\infty} d^3\boldsymbol{\rho}' \left\{ \int_0^t d\tau' \boldsymbol{\mathcal{K}}_{\mathrm{E}}^{(+)}(\mathbf{r},\boldsymbol{\rho}',t-\tau')\Delta\tilde{\chi}(\boldsymbol{\rho}',\tau')\hat{\boldsymbol{\mathcal{E}}}_0(\boldsymbol{\rho}';\tau') \right\}\\
&\quad + \int_{-\infty}^{+\infty} d^3\boldsymbol{\rho}' \left\{ \int_0^t d\tau' \boldsymbol{\mathcal{K}}_{\mathrm{E}}^{(+)}(\mathbf{r},\boldsymbol{\rho}',t-\tau')\Delta\tilde{\chi}(\boldsymbol{\rho}',\tau') \left[\int_{-\infty}^{+\infty} d^3\boldsymbol{\rho}'' \left\{ \int_0^{\tau'} d\tau'' \boldsymbol{\mathcal{K}}_{\mathrm{E}}(\boldsymbol{\rho}',\boldsymbol{\rho}'',\tau'-\tau'')\Delta\tilde{\chi}(\boldsymbol{\rho}'';\tau'')\hat{\boldsymbol{\mathcal{E}}}(\boldsymbol{\rho}'';\tau'') \right\} \right] \right\}\\
&= \hat{\boldsymbol{\mathcal{E}}}_0^{(+)}(\mathbf{r};t) + \int_{-\infty}^{+\infty} d^3\boldsymbol{\rho}' \left\{ \int_0^t d\tau' \boldsymbol{\mathcal{K}}_{\mathrm{E}}^{(+)}(\mathbf{r},\boldsymbol{\rho}',t-\tau')\Delta\tilde{\chi}(\boldsymbol{\rho}',\tau')\hat{\boldsymbol{\mathcal{E}}}_0(\boldsymbol{\rho}';\tau') \right\}\\
&\quad + \int_{-\infty}^{+\infty} d^3\boldsymbol{\rho}' \left\{ \int_0^t d\tau' \boldsymbol{\mathcal{K}}_{\mathrm{E}}^{(+)}(\mathbf{r},\boldsymbol{\rho}',t-\tau')\Delta\tilde{\chi}(\boldsymbol{\rho}',\tau') \left[\int_{-\infty}^{+\infty} d^3\boldsymbol{\rho}'' \left\{ \int_0^{\tau'} d\tau'' \boldsymbol{\mathcal{K}}_{\mathrm{E}}(\boldsymbol{\rho}',\boldsymbol{\rho}'',\tau'-\tau'')\Delta\tilde{\chi}(\boldsymbol{\rho}'';\tau'')\hat{\boldsymbol{\mathcal{E}}}_0(\boldsymbol{\rho}'';\tau'') \right\} \right] \right\}\\
&\quad + \int_{-\infty}^{+\infty} d^3\boldsymbol{\rho}' \left\{ \int_0^t d\tau' \boldsymbol{\mathcal{K}}_{\mathrm{E}}^{(+)}(\mathbf{r},\boldsymbol{\rho}',t-\tau')\Delta\tilde{\chi}(\boldsymbol{\rho}',\tau') \left[\int_{-\infty}^{+\infty} d^3\boldsymbol{\rho}'' \left\{ \int_0^{\tau'} d\tau'' \boldsymbol{\mathcal{K}}_{\mathrm{E}}(\boldsymbol{\rho}',\boldsymbol{\rho}'',\tau'-\tau'')\Delta\tilde{\chi}(\boldsymbol{\rho}'';\tau'')\hat{\boldsymbol{\mathcal{E}}}_{\mathrm{T}}(\boldsymbol{\rho}'';\tau'') \right\} \right] \right\},\end{aligned}$$

with $\hat{\boldsymbol{\mathcal{E}}}_{0/\mathrm{T}} = \hat{\boldsymbol{\mathcal{E}}}_{0/\mathrm{T}}^{(+)} + \hat{\boldsymbol{\mathcal{E}}}_{0/\mathrm{T}}^{(-)}$ and $\boldsymbol{\mathcal{K}}_{\mathrm{E}} = \boldsymbol{\mathcal{K}}_{\mathrm{E}}^{(+)} + \boldsymbol{\mathcal{K}}_{\mathrm{E}}^{(-)}$. According to the above result, it is clear that we can express the electric field operator as a sort of series expansion of successive higher-order terms:

$$\hat{\boldsymbol{\mathcal{E}}}^{(+)}(\mathbf{r};t) = \sum_{i=0}^{\infty} \hat{\boldsymbol{\mathcal{E}}}_i^{(+)}(\mathbf{r};t), \tag{S.III.17}$$

where:

$$\hat{\boldsymbol{\mathcal{E}}}_0^{(+)}(\mathbf{r};t) = \hat{\boldsymbol{\mathcal{E}}}_0^{(+)}(\mathbf{r};t), \tag{S.III.17a}$$

$$\hat{\boldsymbol{\mathcal{E}}}_1^{(+)}(\mathbf{r};t) = \int_{-\infty}^{+\infty} d^3\boldsymbol{\rho}' \int_0^t d\tau' \boldsymbol{\mathcal{K}}_{\mathrm{E}}^{(+)}(\mathbf{r},\boldsymbol{\rho}',t-\tau')\Delta\tilde{\chi}(\boldsymbol{\rho}',\tau')\hat{\boldsymbol{\mathcal{E}}}_0(\boldsymbol{\rho}';\tau'), \tag{S.III.17b}$$

$$\hat{\boldsymbol{\mathcal{E}}}_2^{(+)}(\mathbf{r};t) = \int_{-\infty}^{+\infty} d^3\boldsymbol{\rho}' \int_{-\infty}^{+\infty} d^3\boldsymbol{\rho}'' \int_0^t d\tau' \int_0^{\tau'} d\tau'' \boldsymbol{\mathcal{K}}_{\mathrm{E}}^{(+)}(\mathbf{r},\boldsymbol{\rho}',t-\tau')\Delta\tilde{\chi}(\boldsymbol{\rho}',\tau')\boldsymbol{\mathcal{K}}_{\mathrm{E}}(\boldsymbol{\rho}',\boldsymbol{\rho}'',\tau'-\tau'')\Delta\tilde{\chi}(\boldsymbol{\rho}'',\tau'')\hat{\boldsymbol{\mathcal{E}}}_0(\boldsymbol{\rho}'';\tau''), \tag{S.III.17c}$$

$$\vdots$$

$$\hat{\boldsymbol{\mathcal{E}}}_N^{(+)}(\mathbf{r};t) = \int_{-\infty}^{+\infty} d^3\boldsymbol{\rho}' \int_{-\infty}^{+\infty} d^3\boldsymbol{\rho}'' \ldots \int_{-\infty}^{+\infty} d^3\boldsymbol{\rho}^N \int_0^t d\tau' \int_0^{\tau'} d\tau'' \ldots \int_0^{\tau^{N-1}} d\tau^N \boldsymbol{\mathcal{K}}_{\mathrm{E}}^{(+)}(\mathbf{r},\boldsymbol{\rho}',t-\tau')\tilde{\boldsymbol{\Xi}}^N(\boldsymbol{\rho}',\boldsymbol{\rho}'',\ldots,\boldsymbol{\rho}^N,\tau',\tau'',\ldots,\tau^N), \tag{S.III.17d}$$

with

$$\begin{aligned}\tilde{\boldsymbol{\Xi}}^N(\boldsymbol{\rho}',\boldsymbol{\rho}'',\ldots,\boldsymbol{\rho}^N,\tau',\tau'',\ldots,\tau^N) \equiv{}& \Delta\tilde{\chi}(\boldsymbol{\rho}',\tau')\boldsymbol{\mathcal{K}}_{\mathrm{E}}(\boldsymbol{\rho}',\boldsymbol{\rho}'',\tau'-\tau'')\Delta\tilde{\chi}(\boldsymbol{\rho}'',\tau'')\boldsymbol{\mathcal{K}}_{\mathrm{E}}(\boldsymbol{\rho}'',\boldsymbol{\rho}''',\tau''-\tau''')\ldots\\
&\ldots\Delta\tilde{\chi}(\boldsymbol{\rho}^{N-1},\tau^{N-1})\boldsymbol{\mathcal{K}}_{\mathrm{E}}(\boldsymbol{\rho}^{N-1},\boldsymbol{\rho}^N,\tau^{N-1}-\tau^N)\Delta\tilde{\chi}(\boldsymbol{\rho}^N,\tau^N)\hat{\boldsymbol{\mathcal{E}}}_0(\boldsymbol{\rho}^N;\tau^N).\end{aligned} \tag{S.III.18}$$

As indicated previously, to calculate the total contribution of the complex-like electric field operator in the frequency domain, we take the Laplace transform over each of the terms given above, so that:

$$\hat{\mathbf{E}}_0^{(+)}(\mathbf{r};\omega) = 2\pi \int_{-\infty}^{+\infty} d^3\boldsymbol{\rho}\mathbf{G}_{\mathrm{E}}(\mathbf{r},\boldsymbol{\rho},\omega)\hat{\mathbf{f}}(\boldsymbol{\rho},\omega;t=0), \tag{S.III.19a}$$

$$\hat{\mathbf{E}}_1^{(+)}(\mathbf{r};\omega) = \int_{-\infty}^{+\infty} d^3\boldsymbol{\rho}'\mathbf{K}_{\mathrm{E}}^{(+)}(\mathbf{r},\boldsymbol{\rho}',\omega)\mathcal{L}_\omega[\Delta\tilde{\chi}(\boldsymbol{\rho}',\tau')\hat{\boldsymbol{\mathcal{E}}}_0(\boldsymbol{\rho}';\tau')], \tag{S.III.19b}$$

$$\hat{\mathbf{E}}_2^{(+)}(\mathbf{r};\omega) = \iint_{-\infty}^{+\infty} d^3\boldsymbol{\rho}'d^3\boldsymbol{\rho}''\mathbf{K}_{\mathrm{E}}^{(+)}(\mathbf{r},\boldsymbol{\rho}',\omega)\left\{\int_0^{+\infty} d\tau'' \int_0^{+\infty} d\tilde{\tau}'\Delta\tilde{\chi}(\boldsymbol{\rho}',\tilde{\tau}'+\tau'')\boldsymbol{\mathcal{K}}_{\mathrm{E}}(\boldsymbol{\rho}',\boldsymbol{\rho}'',\tilde{\tau}')\Delta\tilde{\chi}(\boldsymbol{\rho}'',\tau'')\hat{\boldsymbol{\mathcal{E}}}_0(\boldsymbol{\rho}'';\tau'')e^{i\omega\tilde{\tau}'}e^{i\omega\tau''}\right\}, \tag{S.III.19c}$$

where $\tilde{t} = t-\tau'$ and $\tilde{\tau}' = \tau'-\tau''$. From this latter expression it can be observed that the second-order term, $\hat{\mathbf{E}}_2^{(+)}(\mathbf{r};\omega)$, gives rise to some mixing (or coupling) terms involving ancillary times appearing in the time-varying susceptibility function, $\Delta\tilde{\chi}(\boldsymbol{\rho}',\tilde{\tau}'+\tau'')$. This in turn would lead to a intertwining of Laplace transforms, hindering so the simplification of the expression to get a sort of nested (or concatenated) product of frequency-dependent functions. Still, one can take into consideration the following property of Laplace transforms relating products to convolutions:

$$\mathcal{L}_\omega[f(t)g(t)] \equiv \int_0^{+\infty} dtf(t)g(t)e^{i\omega t} = \frac{1}{2\pi}\lim_{\xi\to\infty}\int_{-\xi+i\zeta}^{+\xi+i\zeta} d\omega' F(\omega')G(\omega-\omega') = \frac{1}{2\pi}\int_{-\infty+i\zeta}^{+\infty+i\zeta} d\omega' F(\omega')G(\omega-\omega'), \tag{S.III.20}$$

where it has been used the following definition for the Laplace transform: $\mathcal{L}_\omega[f(t)] \equiv F(\omega) \equiv \int_0^{+\infty} dtf(t)e^{i\omega t}$. It is important to note that the convergence of $F(\omega')$ should be ensured along the horizontal line $\mathrm{Im}[\omega'] = \zeta$, that could be set to $\zeta \to 0^+$. By proceeding in this way the computation merely reduces to only one integral (eventually requiring the application of *Cauchy's residue theorem*), simplifying so considerably the mathematics. Then, the above results can be recast as:

$$\hat{\mathbf{E}}_0^{(+)}(\mathbf{r};\omega) = 2\pi \int_{-\infty}^{+\infty} d^3\boldsymbol{\rho}\mathbf{G}_{\mathrm{E}}(\mathbf{r},\boldsymbol{\rho},\omega)\hat{\mathbf{f}}(\boldsymbol{\rho},\omega;t=0), \tag{S.III.21a}$$

$$\hat{\mathbf{E}}_1^{(+)}(\mathbf{r};\omega) = \int_{-\infty}^{+\infty} d^3\boldsymbol{\rho}'\mathbf{K}_{\mathrm{E}}^{(+)}(\mathbf{r},\boldsymbol{\rho}',\omega)\frac{1}{2\pi}\int_{-\infty+i0^+}^{+\infty+i0^+} d\omega'\Delta\tilde{\chi}(\boldsymbol{\rho}',\omega-\omega')\hat{\mathbf{E}}_0(\boldsymbol{\rho}';\omega'), \tag{S.III.21b}$$

$$\hat{\mathbf{E}}_2^{(+)}(\mathbf{r};\omega) = \iint_{-\infty}^{+\infty} d^3\boldsymbol{\rho}'d^3\boldsymbol{\rho}''\mathbf{K}_{\mathrm{E}}^{(+)}(\mathbf{r},\boldsymbol{\rho}',\omega)\left[\frac{1}{2\pi}\right]^2\iint_{-\infty+i0^+}^{+\infty+i0^+} d\omega'd\omega''\Delta\tilde{\chi}(\boldsymbol{\rho}',\omega-\omega')\mathbf{K}_{\mathrm{E}}(\boldsymbol{\rho}',\boldsymbol{\rho}'',\omega')\Delta\tilde{\chi}(\boldsymbol{\rho}'',\omega'-\omega'')\hat{\mathbf{E}}_0(\boldsymbol{\rho}'';\omega''), \tag{S.III.21c}$$

$$\vdots$$

$$\hat{\mathbf{E}}_N^{(+)}(\mathbf{r};\omega) = \int_{-\infty}^{+\infty} d^3\boldsymbol{\rho}'\int_{-\infty}^{+\infty} d^3\boldsymbol{\rho}''\ldots\int_{-\infty}^{+\infty} d^3\boldsymbol{\rho}^N\mathbf{K}_{\mathrm{E}}^{(+)}(\mathbf{r},\boldsymbol{\rho}',\omega)\int_{-\infty+i0^+}^{+\infty+i0^+} d\omega'\int_{-\infty+i0^+}^{+\infty+i0^+} d\omega''\ldots\int_{-\infty+i0^+}^{+\infty+i0^+} d\omega^N\boldsymbol{\Xi}^N(\boldsymbol{\rho}',\boldsymbol{\rho}'',\ldots,\boldsymbol{\rho}^N,\omega',\omega'',\ldots,\omega^N), \tag{S.III.21d}$$

with

$$\boldsymbol{\Xi}^N(\boldsymbol{\rho}',\boldsymbol{\rho}'',\ldots,\boldsymbol{\rho}^N,\omega',\omega'',\ldots,\omega^N) = \left[\frac{1}{2\pi}\right]^N \Delta\tilde{\chi}(\boldsymbol{\rho}',\omega-\omega')\mathbf{K}_{\mathrm{E}}(\boldsymbol{\rho}',\boldsymbol{\rho}'',\omega')\Delta\tilde{\chi}(\boldsymbol{\rho}'',\omega'-\omega'')\mathbf{K}_{\mathrm{E}}(\boldsymbol{\rho}'',\boldsymbol{\rho}''',\omega'')\ldots$$
$$\ldots\ \Delta\tilde{\chi}(\boldsymbol{\rho}^{N-1},\omega^{N-2}-\omega^{N-1})\mathbf{K}_{\mathrm{E}}(\boldsymbol{\rho}^{N-1},\boldsymbol{\rho}^N,\omega^{N-1})\Delta\tilde{\chi}(\boldsymbol{\rho}^N,\omega^{N-1}-\omega^N)\hat{\mathbf{E}}(\boldsymbol{\rho}^N;\omega^N). \tag{S.III.22}$$

IV. CURRENT DENSITY OPERATORS: DEFINITIONS AND CORRELATIONS

In this section we calculate the current density operator associated to the fields. Such a calculation can be made just by a simple comparison of the previous expressions with the following definition for the electric field operator:

$$\hat{\mathbf{E}}(\mathbf{r};\omega) = i\omega\mu_0 \left[\int_{-\infty}^{+\infty} d^3\boldsymbol{\rho}\mathbf{G}(\mathbf{r},\boldsymbol{\rho},\omega)\cdot\hat{\mathbf{j}}(\boldsymbol{\rho};\omega) + \int_{-\infty}^{+\infty} d^3\boldsymbol{\rho}\mathbf{G}^*(\mathbf{r},\boldsymbol{\rho},\omega)\cdot\hat{\mathbf{j}}^\dagger(\boldsymbol{\rho};\omega)\right] = \hat{\mathbf{E}}^{(+)}(\mathbf{r};\omega) + \hat{\mathbf{E}}^{(-)}(\mathbf{r};\omega). \quad \text{(S.IV.1)}$$

Then, since

$$\hat{\mathbf{E}}_0^{(+)}(\mathbf{r};\omega) = 2\pi \int_{-\infty}^{+\infty} d^3\boldsymbol{\rho}\mathbf{G}_{\mathrm{E}}(\mathbf{r},\boldsymbol{\rho},\omega)\hat{\mathbf{f}}(\boldsymbol{\rho},\omega;t=0) = 2\pi i\sqrt{\frac{\hbar}{\pi\varepsilon_0}}\left(\frac{\omega}{c}\right)^2 \int_{-\infty}^{+\infty} d^3\boldsymbol{\rho}\sqrt{\mathrm{Im}[\varepsilon(\boldsymbol{\rho},\omega)]}\mathbf{G}(\mathbf{r},\boldsymbol{\rho},\omega)\hat{\mathbf{f}}(\boldsymbol{\rho},\omega;t=0),$$

it follows that:

$$\hat{\mathbf{j}}_0(\boldsymbol{\rho};\omega) = \omega\sqrt{4\pi\varepsilon_0\hbar}\sqrt{\mathrm{Im}[\varepsilon(\boldsymbol{\rho},\omega)]}\hat{\mathbf{f}}(\boldsymbol{\rho},\omega;t=0). \quad \text{(S.IV.2)}$$

Similarly, for the first-order electric field,

$$\hat{\mathbf{E}}_1^{(+)}(\mathbf{r};\omega) = \frac{2\omega^2}{\varepsilon_0 c^2}\int_{-\infty}^{+\infty} d^3\boldsymbol{\rho}'\mathbf{G}(\mathbf{r},\boldsymbol{\rho}',\omega)\frac{1}{2\pi}\int_{-\infty+i0^+}^{+\infty+i0^+} d\omega'\Delta\tilde{\chi}(\boldsymbol{\rho}',\omega-\omega')\hat{\mathbf{E}}_0(\boldsymbol{\rho}';\omega'),$$

the associated current density operator reads as:

$$\hat{\mathbf{j}}_1(\boldsymbol{\rho};\omega) = \frac{\omega\mu_0}{\pi}\int_{-\infty}^{+\infty} d^3\boldsymbol{\rho}' \int_{-\infty+i0^+}^{+\infty+i0^+} d\omega'\omega'\Delta\tilde{\chi}(\boldsymbol{\rho},\omega-\omega')\left[\mathbf{G}(\boldsymbol{\rho},\boldsymbol{\rho}',\omega')\hat{\mathbf{j}}_0(\boldsymbol{\rho}';\omega') + \mathbf{G}^*(\boldsymbol{\rho},\boldsymbol{\rho}',\omega')\hat{\mathbf{j}}_0^\dagger(\boldsymbol{\rho}';\omega')\right]. \quad \text{(S.IV.3)}$$

Likewise, for the second-order field,

$$\hat{\mathbf{E}}_2^{(+)}(\mathbf{r};\omega) = \frac{2\omega^2}{\varepsilon_0 c^2}\iint_{-\infty}^{+\infty} d^3\boldsymbol{\rho}'d^3\boldsymbol{\rho}''\mathbf{G}(\mathbf{r},\boldsymbol{\rho}',\omega)\left[\frac{1}{2\pi}\right]^2\iint_{-\infty+i0^+}^{+\infty+i0^+} d\omega'd\omega''\Delta\tilde{\chi}(\boldsymbol{\rho}',\omega-\omega')\mathbf{K}_{\mathrm{E}}(\boldsymbol{\rho}',\boldsymbol{\rho}'',\omega')\Delta\tilde{\chi}(\boldsymbol{\rho}'',\omega'-\omega'')\hat{\mathbf{E}}_0(\boldsymbol{\rho}'';\omega''),$$

and the corresponding current density operator is:

$$\hat{\mathbf{j}}_2(\boldsymbol{\rho};\omega) = \frac{\omega\mu_0}{\pi}\int_{-\infty}^{+\infty} d^3\boldsymbol{\rho}' \int_{-\infty+i0^+}^{+\infty+i0^+} d\omega'\omega'\Delta\tilde{\chi}(\boldsymbol{\rho},\omega-\omega')\mathbf{G}(\boldsymbol{\rho},\boldsymbol{\rho}',\omega')\hat{\mathbf{j}}_1(\boldsymbol{\rho}';\omega'). \quad \text{(S.IV.4)}$$

These expressions allow us to perform the calculation of the corresponding nth-order correlations. To do that, we evaluate $\langle\hat{\mathbf{j}}_l^\dagger(\boldsymbol{\rho};\omega)\cdot\hat{\mathbf{j}}_m(\boldsymbol{\rho}';\omega')\rangle_{\mathrm{th}}$, where $l+m=n$, and the subscript th stands for thermal average, namely, $\langle\ldots\rangle_{\mathrm{th}} \equiv \mathrm{Tr}[\ldots\hat{\varrho}_{\mathrm{th}}]$, with $\hat{\varrho}_{\mathrm{th}}$ being the *canonical density operator* of electromagnetic fields in thermal equilibrium at temperature T (i.e., a *thermal field*). Then, the **zeroth-order correlation** of the current density reads as:

$$\begin{aligned}\langle\hat{\mathbf{j}}_0^\dagger(\boldsymbol{\rho};\omega)\cdot\hat{\mathbf{j}}_0(\boldsymbol{\rho}';\omega')\rangle_{\mathrm{th}} &= 4\pi\varepsilon_0\hbar\omega\omega'\sqrt{\mathrm{Im}[\varepsilon(\boldsymbol{\rho},\omega)]}\sqrt{\mathrm{Im}[\varepsilon(\boldsymbol{\rho}',\omega')]}\,\langle\hat{\mathbf{f}}^\dagger(\boldsymbol{\rho},\omega)\hat{\mathbf{f}}(\boldsymbol{\rho}',\omega')\rangle_{\mathrm{th}}\\ &= \omega'\mathcal{S}(\boldsymbol{\rho};\omega;T)\delta[\boldsymbol{\rho}-\boldsymbol{\rho}']\delta[\omega-\omega'],\end{aligned} \quad \text{(S.IV.5)}$$

where

$$\mathcal{S}(\boldsymbol{\rho};\omega;T) \equiv 4\pi\varepsilon_0\mathrm{Im}[\varepsilon(\boldsymbol{\rho},\omega)]\hbar\omega\Theta(\omega,T), \quad \text{(S.IV.6)}$$

and $\Theta(\omega,T) = [e^{\hbar\omega/(k_B T)}-1]^{-1}$ stands for the average number of thermal photons, with $\hbar$ and k_B being, respectively, the reduced Planck's and the Boltzmann constants.

Equation (S.IV.5) is a very well-known result that provides the correlation of fluctuating currents in absence of any kind of time-modulation. Next, and from this result, one can straightforwardly calculate the **first-order correlation**:

$$\begin{aligned}
\langle \hat{\mathbf{j}}_0^\dagger(\boldsymbol{\rho};\omega)\cdot\hat{\mathbf{j}}_1(\boldsymbol{\rho}';\omega')\rangle_{\rm th} &= \frac{\omega'\mu_0}{\pi}\int_{-\infty}^{+\infty} d^3\boldsymbol{\rho}''\left[\int_{-\infty+i0^+}^{+\infty+i0^+} d\omega''\omega''\Delta\tilde{\chi}(\boldsymbol{\rho}',\omega'-\omega'')\mathbf{G}(\boldsymbol{\rho}',\boldsymbol{\rho}'',\omega'')\,\langle \hat{\mathbf{j}}_0^\dagger(\boldsymbol{\rho};\omega)\cdot\hat{\mathbf{j}}_0(\boldsymbol{\rho}'';\omega'')\rangle_{\rm th}\right]\\
&= \omega' 4\pi\varepsilon_0 {\rm Im}[\varepsilon(\boldsymbol{\rho},\omega)]\hbar\omega\Theta(\omega,T)\left[\frac{\mu_0}{\pi}\int_{-\infty+i0^+}^{+\infty+i0^+} d\omega''(\omega'')^2\Delta\tilde{\chi}(\boldsymbol{\rho}',\omega'-\omega'')\mathbf{G}(\boldsymbol{\rho}',\boldsymbol{\rho},\omega'')\delta[\omega-\omega'']\right]\\
&= \omega'\mathcal{S}(\boldsymbol{\rho};\omega;T)\left[\frac{\mu_0\omega^2}{\pi}\Delta\tilde{\chi}(\boldsymbol{\rho}',\omega'-\omega)\mathbf{G}(\boldsymbol{\rho}',\boldsymbol{\rho},\omega)\right],\\
\langle \hat{\mathbf{j}}_1^\dagger(\boldsymbol{\rho};\omega)\cdot\hat{\mathbf{j}}_0(\boldsymbol{\rho}';\omega')\rangle_{\rm th} &= \frac{\omega\mu_0}{\pi}\int_{-\infty}^{+\infty} d^3\boldsymbol{\rho}''\left[\int_{-\infty+i0^+}^{+\infty+i0^+} d\omega''\omega''\Delta\tilde{\chi}^*(\boldsymbol{\rho},\omega-\omega'')\mathbf{G}^*(\boldsymbol{\rho},\boldsymbol{\rho}'',\omega'')\,\langle \hat{\mathbf{j}}_0^\dagger(\boldsymbol{\rho}'';\omega'')\cdot\hat{\mathbf{j}}_0(\boldsymbol{\rho}';\omega')\rangle_{\rm th}\right]\\
&= \omega 4\pi\varepsilon_0 {\rm Im}[\varepsilon(\boldsymbol{\rho}',\omega')]\hbar\omega'\Theta(\omega',T)\left[\frac{\mu_0}{\pi}\int_{-\infty+i0^+}^{+\infty+i0^+} d\omega''(\omega'')^2\Delta\tilde{\chi}^*(\boldsymbol{\rho},\omega-\omega'')\mathbf{G}^*(\boldsymbol{\rho},\boldsymbol{\rho}',\omega'')\delta[\omega''-\omega']\right]\\
&= \omega\mathcal{S}(\boldsymbol{\rho}';\omega';T)\left[\frac{\mu_0\omega'^2}{\pi}\Delta\tilde{\chi}^*(\boldsymbol{\rho},\omega-\omega')\mathbf{G}^*(\boldsymbol{\rho},\boldsymbol{\rho}',\omega')\right],
\end{aligned}$$

so that:

$$\begin{aligned}
\langle \hat{\mathbf{j}}_0^\dagger(\boldsymbol{\rho};\omega)\hat{\mathbf{j}}_1(\boldsymbol{\rho}';\omega') + \hat{\mathbf{j}}_1^\dagger(\boldsymbol{\rho};\omega)\hat{\mathbf{j}}_0(\boldsymbol{\rho}';\omega')\rangle_{\rm th} &= \frac{\omega\omega'\mu_0}{\pi}\left[\omega\mathcal{S}(\boldsymbol{\rho};\omega;T)\Delta\tilde{\chi}(\boldsymbol{\rho}',\omega'-\omega)\mathbf{G}(\boldsymbol{\rho}',\boldsymbol{\rho},\omega)\right]\\
&+ \frac{\omega\omega'\mu_0}{\pi}\left[\omega'\mathcal{S}(\boldsymbol{\rho}',\omega';T)\Delta\tilde{\chi}^*(\boldsymbol{\rho},\omega-\omega')\mathbf{G}^*(\boldsymbol{\rho},\boldsymbol{\rho}',\omega')\right].
\end{aligned} \quad \text{(S.IV.7)}$$

Similarly, from the above result one could calculate the **second-order correlation**:

$$\begin{aligned}
\langle \hat{\mathbf{j}}_0^\dagger(\boldsymbol{\rho};\omega)\cdot\hat{\mathbf{j}}_2(\boldsymbol{\rho}';\omega')\rangle_{\rm th} &= \frac{\omega'\mu_0}{\pi}\int_{-\infty}^{+\infty} d^3\boldsymbol{\rho}''\left[\int_{-\infty+i0^+}^{+\infty+i0^+} d\omega''\omega''\Delta\tilde{\chi}(\boldsymbol{\rho}',\omega'-\omega'')\mathbf{G}(\boldsymbol{\rho}',\boldsymbol{\rho}'',\omega'')\,\langle \hat{\mathbf{j}}_0^\dagger(\boldsymbol{\rho};\omega)\cdot\hat{\mathbf{j}}_1(\boldsymbol{\rho}'';\omega'')\rangle_{\rm th}\right]\\
&= \omega'\mathcal{S}(\boldsymbol{\rho};\omega;T)\left[\frac{\mu_0\omega}{\pi}\right]^2\int_{-\infty}^{+\infty} d^3\boldsymbol{\rho}''\left[\int_{-\infty+i0^+}^{+\infty+i0^+} d\omega''(\omega'')^2\Delta\tilde{\chi}(\boldsymbol{\rho}',\omega'-\omega'')\mathbf{G}(\boldsymbol{\rho}',\boldsymbol{\rho}'',\omega'')\Delta\tilde{\chi}(\boldsymbol{\rho}'',\omega''-\omega)\mathbf{G}(\boldsymbol{\rho}'',\boldsymbol{\rho},\omega)\right],\\
\langle \hat{\mathbf{j}}_2^\dagger(\boldsymbol{\rho};\omega)\cdot\hat{\mathbf{j}}_0(\boldsymbol{\rho}';\omega')\rangle_{\rm th} &= \frac{\omega\mu_0}{\pi}\int_{-\infty}^{+\infty} d^3\boldsymbol{\rho}''\left[\int_{-\infty+i0^+}^{+\infty+i0^+} d\omega''\omega''\Delta\tilde{\chi}^*(\boldsymbol{\rho},\omega-\omega'')\mathbf{G}^*(\boldsymbol{\rho},\boldsymbol{\rho}'',\omega'')\,\langle \hat{\mathbf{j}}_1^\dagger(\boldsymbol{\rho}'';\omega'')\cdot\hat{\mathbf{j}}_0(\boldsymbol{\rho}';\omega')\rangle_{\rm th}\right]\\
&= \omega\mathcal{S}(\boldsymbol{\rho}';\omega';T)\left[\frac{\mu_0\omega'}{\pi}\right]^2\int_{-\infty}^{+\infty} d^3\boldsymbol{\rho}''\left[\int_{-\infty+i0^+}^{+\infty+i0^+} d\omega''(\omega'')^2\Delta\tilde{\chi}^*(\boldsymbol{\rho},\omega-\omega'')\mathbf{G}^*(\boldsymbol{\rho},\boldsymbol{\rho}'',\omega'')\Delta\tilde{\chi}^*(\boldsymbol{\rho}'',\omega''-\omega')\mathbf{G}^*(\boldsymbol{\rho}'',\boldsymbol{\rho}',\omega')\right],
\end{aligned}$$

$$\begin{aligned}
\langle \hat{\mathbf{j}}_1^\dagger(\boldsymbol{\rho};\omega)\cdot\hat{\mathbf{j}}_1(\boldsymbol{\rho}';\omega')\rangle_{\rm th} &= \omega\omega'\left[\frac{\mu_0}{\pi}\right]^2\iint_{-\infty}^{+\infty} d^3\tilde{\boldsymbol{\rho}}d^3\tilde{\boldsymbol{\rho}}'\iint_{-\infty+i0^+}^{+\infty+i0^+} d\tilde{\omega}d\tilde{\omega}'\tilde{\omega}\tilde{\omega}'\Delta\tilde{\chi}^*(\boldsymbol{\rho},\omega-\tilde{\omega})\Delta\tilde{\chi}(\boldsymbol{\rho}',\omega'-\tilde{\omega}')\\
&\left[\mathbf{G}^*(\boldsymbol{\rho},\tilde{\boldsymbol{\rho}},\tilde{\omega})\mathbf{G}(\boldsymbol{\rho}',\tilde{\boldsymbol{\rho}}',\tilde{\omega}')\,\langle \hat{\mathbf{j}}_0^\dagger(\tilde{\boldsymbol{\rho}};\tilde{\omega})\cdot\hat{\mathbf{j}}_0(\tilde{\boldsymbol{\rho}}';\tilde{\omega}')\rangle_{\rm th} + \mathbf{G}(\boldsymbol{\rho},\tilde{\boldsymbol{\rho}},\tilde{\omega})\mathbf{G}^*(\boldsymbol{\rho}',\tilde{\boldsymbol{\rho}}',\tilde{\omega}')\,\langle \hat{\mathbf{j}}_0(\tilde{\boldsymbol{\rho}};\tilde{\omega})\cdot\hat{\mathbf{j}}_0^\dagger(\tilde{\boldsymbol{\rho}}';\tilde{\omega}')\rangle_{\rm th}\right]\\
&= 4\hbar\omega\omega'\left[\frac{\mu_0}{\pi}\right]\int_{-\infty+i0^+}^{+\infty+i0^+} d\tilde{\omega}(\tilde{\omega})^2\Delta\tilde{\chi}^*(\boldsymbol{\rho},\omega-\tilde{\omega})\Delta\tilde{\chi}(\boldsymbol{\rho}',\omega'-\tilde{\omega})\left\{{\rm Im}[\mathbf{G}(\boldsymbol{\rho},\boldsymbol{\rho}',\tilde{\omega})]\left[1+\Theta(\tilde{\omega},T)\right]+\Theta(\tilde{\omega},T){\rm Im}[\mathbf{G}(\boldsymbol{\rho}',\boldsymbol{\rho},\tilde{\omega})]\right\}.
\end{aligned}$$

V. ELECTRIC-FIELD CORRELATIONS

In this section, we shall proceed with the calculation of the electric-field correlations of successive orders:

- **Zeroth-order E-field correlations:**

$$\mathcal{S}_{0,0}(\mathbf{r};\omega;T) = \langle(\hat{\mathbf{E}}_0^{(+)}(\mathbf{r};\omega))^\dagger \cdot \hat{\mathbf{E}}_0^{(+)}(\mathbf{r};\omega)\rangle_{\text{th}} = \omega^2\mu_0^2 \iint_{-\infty}^{+\infty} d^3\boldsymbol{\rho} d^3\boldsymbol{\rho}' \mathbf{G}^*(\mathbf{r},\boldsymbol{\rho},\omega)\mathbf{G}(\mathbf{r},\boldsymbol{\rho}',\omega)\, \langle\hat{\mathbf{j}}_0^\dagger(\boldsymbol{\rho};\omega)\cdot\hat{\mathbf{j}}_0(\boldsymbol{\rho}';\omega)\rangle_{\text{th}}$$

$$= 4\pi\omega\mu_0\hbar\omega\Theta(\omega,T)\int_{-\infty}^{+\infty} d^3\boldsymbol{\rho}\frac{\omega^2}{c^2}\text{Im}[\varepsilon(\boldsymbol{\rho},\omega)]\mathbf{G}^*(\mathbf{r},\boldsymbol{\rho},\omega)\mathbf{G}(\mathbf{r},\boldsymbol{\rho},\omega),$$

$$\mathcal{S}_0(\mathbf{r};\omega;T) = \mathcal{S}_{0,0}(\mathbf{r};\omega;T) = 4\pi\omega\mu_0\hbar\omega\Theta(\omega,T)\text{Im}[\mathbf{G}(\mathbf{r},\mathbf{r},\omega)]. \tag{S.V.1}$$

- **First-order E-field correlations:**

$$\mathcal{S}_{0,1}(\mathbf{r};\omega;T) = \langle(\hat{\mathbf{E}}_0^{(+)}(\mathbf{r};\omega))^\dagger \cdot \hat{\mathbf{E}}_1^{(+)}(\mathbf{r};\omega)\rangle_{\text{th}} = \omega^2\mu_0^2 \iint_{-\infty}^{+\infty} d^3\boldsymbol{\rho} d^3\boldsymbol{\rho}' \mathbf{G}^*(\mathbf{r},\boldsymbol{\rho},\omega)\mathbf{G}(\mathbf{r},\boldsymbol{\rho}',\omega)\, \langle\hat{\mathbf{j}}_0^\dagger(\boldsymbol{\rho};\omega)\cdot\hat{\mathbf{j}}_1(\boldsymbol{\rho}';\omega)\rangle_{\text{th}}$$

$$= 4\pi\omega\mu_0\hbar\omega\Theta(\omega,T)\omega^2\frac{\mu_0}{\pi}\int_{-\infty}^{+\infty} d^3\boldsymbol{\rho}\text{Im}[\mathbf{G}(\boldsymbol{\rho},\mathbf{r},\omega)]\mathbf{G}(\mathbf{r},\boldsymbol{\rho},\omega)\Delta\tilde{\chi}(\boldsymbol{\rho},0),$$

$$\mathcal{S}_1(\mathbf{r};\omega;T) = 2\text{Re}[\mathcal{S}_{0,1}(\mathbf{r};\omega;T)] = 8\omega^3\mu_0^2\hbar\omega\Theta(\omega,T)\int_{-\infty}^{+\infty} d^3\boldsymbol{\rho}\text{Im}[\mathbf{G}(\mathbf{r},\boldsymbol{\rho},\omega)]\text{Re}[\mathbf{G}(\mathbf{r},\boldsymbol{\rho},\omega)\Delta\tilde{\chi}(\boldsymbol{\rho},0)], \tag{S.V.2}$$

where it should be noted that $\mathcal{S}_{1,0}(\mathbf{r};\omega;T) = [\mathcal{S}_{0,1}(\mathbf{r};\omega;T)]^\dagger$. Given that the susceptibility function contains the whole information about the time-modulation, its evaluation at a constant frequency, in this case at $\omega = 0$, gets rid of its whole dependence on the frequency and consequently, of the time-modulation. Therefore, and as a general corollary, the first-order term of the EM field correlations can be generally interpreted as an offset to the zeroth-order contribution, and, eventually, it may be totally neglected.

- **Second-order E-field correlations:**

$$\mathcal{S}_{0,2}(\mathbf{r};\omega;T) = \langle(\hat{\mathbf{E}}_0^{(+)}(\mathbf{r};\omega))^\dagger \cdot \hat{\mathbf{E}}_2^{(+)}(\mathbf{r};\omega)\rangle_{\text{th}} = \omega^2\mu_0^2 \iint_{-\infty}^{+\infty} d^3\boldsymbol{\rho} d^3\boldsymbol{\rho}' \mathbf{G}^*(\mathbf{r},\boldsymbol{\rho},\omega)\mathbf{G}(\mathbf{r},\boldsymbol{\rho}',\omega)\, \langle\hat{\mathbf{j}}_0^\dagger(\boldsymbol{\rho};\omega)\cdot\hat{\mathbf{j}}_2(\boldsymbol{\rho}';\omega)\rangle_{\text{th}}$$

$$= 4\pi\omega\mu_0\hbar\omega\Theta(\omega,T)\frac{\omega^2\mu_0^2}{\pi^2}\iint_{-\infty}^{+\infty} d^3\boldsymbol{\rho} d^3\boldsymbol{\rho}'\mathbf{G}(\mathbf{r},\boldsymbol{\rho},\omega)\text{Im}[\mathbf{G}(\boldsymbol{\rho}',\mathbf{r},\omega)]\int_{-\infty+i0^+}^{+\infty+i0^+} d\tilde{\omega}(\tilde{\omega})^2\Delta\tilde{\chi}(\boldsymbol{\rho},\omega-\tilde{\omega})\Delta\tilde{\chi}(\boldsymbol{\rho}',\tilde{\omega}-\omega)\mathbf{G}(\boldsymbol{\rho},\boldsymbol{\rho}',\tilde{\omega}),$$

$$\mathcal{S}_{1,1}(\mathbf{r};\omega;T) = \langle(\hat{\mathbf{E}}_1^{(+)}(\mathbf{r};\omega))^\dagger \cdot \hat{\mathbf{E}}_1^{(+)}(\mathbf{r};\omega)\rangle_{\text{th}} = \omega^2\mu_0^2 \iint_{-\infty}^{+\infty} d^3\boldsymbol{\rho} d^3\boldsymbol{\rho}' \mathbf{G}^*(\mathbf{r},\boldsymbol{\rho},\omega)\mathbf{G}(\mathbf{r},\boldsymbol{\rho}',\omega)\, \langle\hat{\mathbf{j}}_1^\dagger(\boldsymbol{\rho};\omega)\cdot\hat{\mathbf{j}}_1(\boldsymbol{\rho}';\omega)\rangle_{\text{th}}$$

$$= 4\hbar\omega^4\frac{\mu_0^3}{\pi}\iint_{-\infty}^{+\infty} d^3\boldsymbol{\rho} d^3\boldsymbol{\rho}'\mathbf{G}^*(\mathbf{r},\boldsymbol{\rho},\omega)\mathbf{G}(\mathbf{r},\boldsymbol{\rho}',\omega)\int_{-\infty+i0^+}^{+\infty+i0^+} d\tilde{\omega}\,[1+\Theta(\tilde{\omega},T)]\,\tilde{\omega}^2\Delta\tilde{\chi}^*(\boldsymbol{\rho},\omega-\tilde{\omega})\Delta\tilde{\chi}(\boldsymbol{\rho}',\omega-\tilde{\omega})\text{Im}[\mathbf{G}(\boldsymbol{\rho},\boldsymbol{\rho}',\tilde{\omega})]$$

$$+ 4\hbar\omega^4\frac{\mu_0^3}{\pi}\iint_{-\infty}^{+\infty} d^3\boldsymbol{\rho} d^3\boldsymbol{\rho}'\mathbf{G}^*(\mathbf{r},\boldsymbol{\rho},\omega)\mathbf{G}(\mathbf{r},\boldsymbol{\rho}',\omega)\int_{-\infty+i0^+}^{+\infty+i0^+} d\tilde{\omega}\Theta(\tilde{\omega},T)\tilde{\omega}^2\Delta\tilde{\chi}^*(\boldsymbol{\rho},\omega-\tilde{\omega})\Delta\tilde{\chi}(\boldsymbol{\rho}',\omega-\tilde{\omega})\text{Im}[\mathbf{G}(\boldsymbol{\rho}',\boldsymbol{\rho},\tilde{\omega})].$$

VI. THERMAL EMISSION OF A TIME-MODULATED SEMI-INFINITE HOMOGENEOUS MEDIUM

Angular spectrum representation of the dyadic Green's function

In order to demonstrate the usefulness of the above theoretical formulation on the fluctuation-dissipation theorem for time-varying quantum systems, here below we make use of it to tackle on a practical case in which we analyze the phenomenon of thermal emission. Specifically, we consider a flat interface separating a semi-infinite homogeneous and nonmagnetic medium ($z \leq 0$), held in local thermodynamic equilibrium at a uniform temperature T, from a vacuum ($z > 0$). For such a system, and in general, for any translationally symmetric structure exhibiting a planar geometry (namely, slabs, interfaces, or layered media, including thin-films), one can take advantage of a powerful mathematical formalism commonly referred to as the *angular spectrum representation*. Such an approach allows one to express the dyadic Green's function of a system as a superposition of elementary plane waves [8, 9]:

$$\mathbf{G}(\mathbf{r},\boldsymbol{\rho},\omega) = \frac{k_0^2}{4\pi^2} \iint_{-\infty}^{+\infty} d^2\boldsymbol{\kappa}_{\parallel} \hat{\mathbf{G}}(\kappa_x,\kappa_y;\omega|z,\rho_z) e^{ik_0[\kappa_x(x-\rho_x)+\kappa_y(y-\rho_y)]}. \tag{S.VI.1}$$

Then, assuming a homogeneous, isotropic and linear* medium, it follows that [10]:

$$\mathbf{k}_i = k_0(\kappa_x,\kappa_y,\tilde{k}_{z,i}) = k_0(\kappa_x,\kappa_y,\sqrt{\varepsilon_i(\omega)}\kappa_{z,i}) \;\Rightarrow\; \mathbf{k}_i^2 = k_0^2\left[\kappa_x^2+\kappa_y^2+\tilde{k}_{z,i}^2\right] = k_0^2\left[\kappa_x^2+\kappa_y^2+\varepsilon_i(\omega)\kappa_{z,i}^2\right] = \varepsilon_i(\omega)k_0^2,$$

$$\kappa_R^2+\tilde{k}_{z,i}^2 = \kappa_R^2+\varepsilon_i(\omega)\kappa_{z,i}^2 = \varepsilon_i(\omega) \quad\Longrightarrow\quad \kappa_{z,i} = \sqrt{1-\frac{\kappa_R^2}{\varepsilon_i(\omega)}}, \tag{S.VI.2}$$

with $\tilde{k}_{z,i} = k_{z,i}/k_0 = \sqrt{\varepsilon_i(\omega)}\kappa_{z,i}$, and $\kappa_R^2 \equiv \kappa_x^2+\kappa_y^2$.

Notice that, solely in the case of lossless media (i.e., those in which $\varepsilon_i(\omega)$ is real), it is possible to establish the following usual correspondence:

$$\kappa_{z,i} = \begin{cases} \sqrt{1-\kappa_R^2/\varepsilon_i(\omega)}, & \text{if } \kappa_R^2 \leq \varepsilon_i(\omega) \quad\Longrightarrow\quad \text{propagating modes (far-field)}, \\ i\sqrt{\kappa_R^2/\varepsilon_i(\omega)-1}, & \text{if } \kappa_R^2 > \varepsilon_i(\omega) \quad\Longrightarrow\quad \text{evanescent modes (near-field)}, \end{cases} \tag{S.VI.3}$$

which for the vacuum leads to the most standard form:

$$\kappa_{z,vac} = \begin{cases} \sqrt{1-\kappa_R^2}, & \text{if } \kappa_R^2 \leq 1 \quad\Longrightarrow\quad \text{propagating modes (far-field)}, \\ i\sqrt{\kappa_R^2-1}, & \text{if } \kappa_R^2 > 1 \quad\Longrightarrow\quad \text{evanescent modes (near-field)}. \end{cases} \tag{S.VI.4}$$

Regarding this general framework, henceforth we shall only deal with two different media:

- **medium 1**, that will be the vacuum, so that $\varepsilon_1(\omega) \equiv 1$, and thus having a longitudinal wavevector such that $k_{z,1} = k_0\tilde{k}_{z,1} = k_0\kappa_{z,1}$, with $\kappa_{z,1} = \sqrt{1-\kappa_R^2}$, which will be denoted by means of (x,y,z)-coordinates;
- **medium 2**, that will be a dispersive material, so that $\varepsilon_2(\omega) \equiv \varepsilon(\omega)$, which, in general will be a complex-like function, and thus having a longitudinal wavevector $k_{z,2} = k_0\tilde{k}_{z,2} = k_0\sqrt{\varepsilon(\omega)}\kappa_{z,2}$, with $\kappa_{z,2} = \sqrt{1-\kappa_R^2/\varepsilon(\omega)}$ (that, due to the complex-like character of $\varepsilon(\omega)$, it will not directly fulfill the above correspondence with propagating and evanescent modes). In this case, we shall use (ρ_x,ρ_y,ρ_z)-coordinates to denote this medium†.

---

* Notice that, even though strictly speaking the time-modulation would break down the linear behavior of the medium, according to the Hamiltonian given in Eq. (S.I.1), we are actually assuming a linear response to relate the polarization to the external electric field.

† It should be noted that, when analyzing the contributions resulting from the time-modulation we will need to deal with some auxiliary coordinates of the same medium associated to different currents; they will be denoted with the same coordinates by adding some tildes.

Likewise, and on this basis, it is worth setting down a general formulation to deal with the dyadic notation of the Green's tensors (cf. Refs. [8–11] for further details on it). Specifically, considering a plane interface system as that mentioned above, the Green's tensor relating currents in the lower half-space (medium j; with coordinates $\rho_z \leq 0$) to fields in the upper half-space (medium i; with coordinates $z > 0$), can be generally expressed as:

$$\hat{\mathbf{G}}^{(\updownarrow/\updownarrow)}_{i\leftarrow j}(\kappa_R, \kappa_\varphi; \omega|z, \rho_z) = \frac{i}{2}\frac{1}{k_{z,j}}\left[t^{(\mathrm{s})}_{i\leftarrow j}\hat{\mathbf{g}}^{(\mathrm{s})}_{i\leftarrow j} + t^{(\mathrm{p})}_{i\leftarrow j}\hat{\mathbf{g}}^{(\mathrm{p})(\updownarrow/\updownarrow)}_{i\leftarrow j}\right]\Gamma_{i\leftarrow j}, \tag{S.VI.5}$$

where

$$\hat{\mathbf{g}}^{(\mathrm{s})}_{i\leftarrow j} \equiv \hat{\mathbf{s}}\otimes\hat{\mathbf{s}} = \begin{bmatrix} \sin^2\kappa_\varphi & -\cos\kappa_\varphi\sin\kappa_\varphi & 0 \\ -\cos\kappa_\varphi\sin\kappa_\varphi & \cos^2\kappa_\varphi & 0 \\ 0 & 0 & 0 \end{bmatrix}, \tag{S.VI.6a}$$

$$\hat{\mathbf{g}}^{(\mathrm{p})(\updownarrow/\updownarrow)}_{i\leftarrow j} \equiv \hat{\mathbf{p}}^{\updownarrow}_i\otimes\hat{\mathbf{p}}^{\updownarrow}_j = \begin{bmatrix} \kappa_{z,i}\kappa_{z,j}\cos^2\kappa_\varphi & \kappa_{z,i}\kappa_{z,j}\cos\kappa_\varphi\sin\kappa_\varphi & \frac{\kappa_{z,i}\kappa_R\cos\kappa_\varphi}{(\mp)\sqrt{\varepsilon_j(\omega)}} \\ \kappa_{z,i}\kappa_{z,j}\cos\kappa_\varphi\sin\kappa_\varphi & \kappa_{z,i}\kappa_{z,j}\sin^2\kappa_\varphi & \frac{\kappa_{z,i}\kappa_R\sin\kappa_\varphi}{(\mp)\sqrt{\varepsilon_j(\omega)}} \\ \frac{\kappa_{z,j}\kappa_R\cos\kappa_\varphi}{(\mp)\sqrt{\varepsilon_i(\omega)}} & \frac{\kappa_{z,j}\kappa_R\sin\kappa_\varphi}{(\mp)\sqrt{\varepsilon_i(\omega)}} & \frac{\kappa_R^2}{(\pm)\sqrt{\varepsilon_i(\omega)}(\pm)\sqrt{\varepsilon_j(\omega)}} \end{bmatrix}, \tag{S.VI.6b}$$

result from the dyadic product of the corresponding *polarization-vector basis*,

$$\hat{\mathbf{s}} \equiv \left(\sin\kappa_\varphi, -\cos\kappa_\varphi, 0\right), \tag{S.VI.7a}$$

$$\hat{\mathbf{p}}^{\uparrow/\downarrow}_i \equiv \left(-\kappa_{z,i}\cos\kappa_\varphi, -\kappa_{z,i}\sin\kappa_\varphi, \pm\kappa_R/\sqrt{\varepsilon_i(\omega)}\right), \tag{S.VI.7b}$$

with the signs + and − being associated to modes propagating upward (↑) and downward (↓). Furthermore,

$$t^{(\mathrm{s})}_{i\leftarrow j} = \frac{2\sqrt{\varepsilon_j(\omega)}\kappa_{z,j}}{\sqrt{\varepsilon_i(\omega)}\kappa_{z,i} + \sqrt{\varepsilon_j(\omega)}\kappa_{z,j}}, \tag{S.VI.8a}$$

$$t^{(\mathrm{p})}_{i\leftarrow j} = \frac{2\sqrt{\varepsilon_i(\omega)}\varepsilon_j(\omega)\kappa_{z,j}}{\varepsilon_j(\omega)\sqrt{\varepsilon_i(\omega)}\kappa_{z,i} + \varepsilon_i(\omega)\sqrt{\varepsilon_j(\omega)}\kappa_{z,j}}, \tag{S.VI.8b}$$

stand for the *Fresnel transmission coefficients*, and

$$\Gamma_{i\leftarrow j} = e^{ik_0\left[\sqrt{\varepsilon_i(\omega)}\kappa_{z,i}z - \sqrt{\varepsilon_j(\omega)}\kappa_{z,j}\rho_z\right]}, \tag{S.VI.9}$$

is the *field propagator*, where it has been implicitly assumed that the medium j is underneath the medium i.

On the other hand, in the case of two points lying in the same medium, e.g., in the lower medium j, the above Green's tensor has to be recast as:

$$\begin{aligned}\hat{\mathbf{G}}^{(\updownarrow/\updownarrow)}_{i\leftarrow j}(\kappa_R, \kappa_\varphi; \omega|\rho_z, \rho'_z) = {}& \frac{i}{2}\frac{1}{k_{z,j}}\left\{\left[r^{(\mathrm{s})}_{j\leftarrow i}\hat{\mathbf{g}}^{(\mathrm{s})}_{j\leftarrow j'} + r^{(\mathrm{p})}_{j\leftarrow i}\hat{\mathbf{g}}^{(\mathrm{p})(\downarrow/\uparrow)}_{j\leftarrow j'}\right]e^{-ik_0\sqrt{\varepsilon_j(\omega)}\kappa_{z,j}(\rho_z+\rho'_z)}\right\} \\ &+ \frac{i}{2}\frac{1}{k_{z,j}}\left\{\left[\hat{\mathbf{g}}^{(\mathrm{s})}_{j\leftarrow j'} + \hat{\mathbf{g}}^{(\mathrm{p})(\updownarrow/\updownarrow)}_{j\leftarrow j'}\right]e^{ik_0\sqrt{\varepsilon_j(\omega)}\kappa_{z,j}|\rho_z-\rho'_z|}\right\} - \frac{\delta[\rho_z-\rho'_z]}{k_0^2\varepsilon_j}[\hat{\mathbf{z}}\otimes\hat{\mathbf{z}}],\end{aligned} \tag{S.VI.10}$$

where

$$r^{(\mathrm{s})}_{j\leftarrow i} = \frac{\sqrt{\varepsilon_j(\omega)}\kappa_{z,j} - \sqrt{\varepsilon_i(\omega)}\kappa_{z,i}}{\sqrt{\varepsilon_i(\omega)}\kappa_{z,i} + \sqrt{\varepsilon_j(\omega)}\kappa_{z,j}}, \tag{S.VI.11a}$$

$$r^{(\mathrm{p})}_{j\leftarrow i} = \frac{\varepsilon_i(\omega)\sqrt{\varepsilon_j(\omega)}\kappa_{z,j} - \varepsilon_j(\omega)\sqrt{\varepsilon_i(\omega)}\kappa_{z,i}}{\varepsilon_j(\omega)\sqrt{\varepsilon_i(\omega)}\kappa_{z,i} + \varepsilon_i(\omega)\sqrt{\varepsilon_j(\omega)}\kappa_{z,j}}, \tag{S.VI.11b}$$

are the *Fresnel reflection coefficients*.

Finally, regarding this dyadic notation it is also worth highlighting the following relations:

$$\hat{\mathbf{g}}^{(\mathrm{s})}_{i\leftarrow j}\cdot\hat{\mathbf{g}}^{(\mathrm{s})}_{m\leftarrow n}=[\hat{\mathbf{s}}\otimes\hat{\mathbf{s}}]\cdot[\hat{\mathbf{s}}\otimes\hat{\mathbf{s}}]=\hat{\mathbf{s}}\,[\hat{\mathbf{s}}\cdot\hat{\mathbf{s}}]\,\hat{\mathbf{s}}=\hat{\mathbf{s}}\otimes\hat{\mathbf{s}}=\hat{\mathbf{g}}^{(\mathrm{s})}_{i\leftarrow n}=\hat{\mathbf{g}}^{(\mathrm{s})}, \tag{S.VI.12a}$$

$$\hat{\mathbf{g}}^{(\mathrm{s})}_{i\leftarrow j}\cdot\hat{\mathbf{g}}^{(\mathrm{p})(\updownarrow/\updownarrow)}_{m\leftarrow n}=[\hat{\mathbf{s}}\otimes\hat{\mathbf{s}}]\cdot\left[\hat{\mathbf{p}}^{\updownarrow}_{m}\otimes\hat{\mathbf{p}}^{\updownarrow}_{n}\right]=\hat{\mathbf{s}}\left[\hat{\mathbf{s}}\cdot\hat{\mathbf{p}}^{\updownarrow}_{m}\right]\hat{\mathbf{p}}^{\updownarrow}_{n}=\mathbf{0}, \tag{S.VI.12b}$$

$$\hat{\mathbf{g}}^{(\mathrm{p})(\updownarrow/\updownarrow)}_{i\leftarrow j}\cdot\hat{\mathbf{g}}^{(\mathrm{s})}_{m\leftarrow n}=\left[\hat{\mathbf{p}}^{\updownarrow}_{i}\otimes\hat{\mathbf{p}}^{\updownarrow}_{j}\right]\cdot[\hat{\mathbf{s}}\otimes\hat{\mathbf{s}}]=\hat{\mathbf{p}}^{\updownarrow}_{i}\left[\hat{\mathbf{p}}^{\updownarrow}_{j}\cdot\hat{\mathbf{s}}\right]\hat{\mathbf{s}}=\mathbf{0}, \tag{S.VI.12c}$$

$$\hat{\mathbf{g}}^{(\mathrm{p})(\updownarrow/\updownarrow)}_{i\leftarrow j}\cdot\hat{\mathbf{g}}^{(\mathrm{p})(\updownarrow/\updownarrow)}_{m\leftarrow n}=\left[\hat{\mathbf{p}}^{\updownarrow}_{i}\otimes\hat{\mathbf{p}}^{\updownarrow}_{j}\right]\cdot\left[\hat{\mathbf{p}}^{\updownarrow}_{m}\otimes\hat{\mathbf{p}}^{\updownarrow}_{n}\right]=\hat{\mathbf{p}}^{\updownarrow}_{i}\left[\hat{\mathbf{p}}^{\updownarrow}_{j}\cdot\hat{\mathbf{p}}^{\updownarrow}_{m}\right]\hat{\mathbf{p}}^{\updownarrow}_{n}=\left[\hat{\mathbf{p}}^{\updownarrow}_{j}\cdot\hat{\mathbf{p}}^{\updownarrow}_{m}\right]\hat{\mathbf{g}}^{(\mathrm{p})(\updownarrow/\updownarrow)}_{i\leftarrow n}, \tag{S.VI.12d}$$

with

$$\left[\hat{\mathbf{p}}^{\updownarrow}_{j}\cdot\hat{\mathbf{p}}^{\updownarrow}_{m}\right]=\kappa_{z,j}\kappa_{z,m}+\frac{\kappa_R^2}{(\pm)\sqrt{\varepsilon_j(\omega)}(\pm)\sqrt{\varepsilon_m(\omega)}}. \tag{S.VI.13}$$

Zeroth-order of thermal emission

Taking into account the previous section, the zeroth-order electric field correlation can be recast as follows:

$$\mathcal{S}_{0,0}(\mathbf{r};\omega;T)=\langle[\hat{\mathbf{E}}^{(+)}_0(\mathbf{r};\omega)]^\dagger\cdot\hat{\mathbf{E}}^{(+)}_0(\mathbf{r};\omega)\rangle_{\mathrm{th}}=\omega^2\mu_0^2\iint_{\mathcal{V}}d^3\boldsymbol{\rho}d^3\boldsymbol{\rho}'\mathrm{Tr}[\mathbf{G}^*(\mathbf{r},\boldsymbol{\rho},\omega)\mathbf{G}(\mathbf{r},\boldsymbol{\rho}',\omega)]\,\langle\hat{\mathbf{j}}^\dagger_0(\boldsymbol{\rho};\omega)\cdot\hat{\mathbf{j}}_0(\boldsymbol{\rho}';\omega)\rangle_{\mathrm{th}}$$
$$=\frac{4\pi}{\varepsilon_0}\frac{\omega^3}{c^4}\mathrm{Im}[\varepsilon(\omega)]\hbar\omega\Theta(\omega,T)\left[\int_{\mathcal{V}}d^3\boldsymbol{\rho}\mathrm{Tr}[\mathbf{G}^*(\mathbf{r},\boldsymbol{\rho},\omega)\mathbf{G}(\mathbf{r},\boldsymbol{\rho},\omega)]\right].$$

Then:

$$\mathcal{S}_0(\mathbf{r};\omega;T)=\mathcal{S}_{0,0}(\mathbf{r};\omega;T)=\frac{4\pi}{\varepsilon_0}\frac{\omega^3}{c^4}\mathrm{Im}[\varepsilon(\omega)]\hbar\omega\Theta(\omega,T)\mathcal{G}_{0,0}(\mathbf{r},\omega), \tag{S.VI.14}$$

where

$$\mathcal{G}_{0,0}(\mathbf{r},\omega)=\int_{\mathcal{V}}d^3\boldsymbol{\rho}\mathrm{Tr}[\mathbf{G}^*(\mathbf{r},\boldsymbol{\rho},\omega)\mathbf{G}(\mathbf{r},\boldsymbol{\rho},\omega)], \tag{S.VI.15}$$

with $\mathcal{V}$ being the volume of the material system (i.e., medium 2) occupying the half-space $z\leq 0$.

Calculation of $\mathcal{G}_{0,0}$:

We now proceed with the explicit calculation of $\mathcal{G}_{0,0}$. To do that, we start by substituting Eq. (S.VI.1) into (S.VI.15):

$$\mathcal{G}_{0,0}(\mathbf{r},\omega)=\int_{\mathcal{V}}d^3\boldsymbol{\rho}\mathrm{Tr}[\mathbf{G}^*(\mathbf{r},\boldsymbol{\rho},\omega)\mathbf{G}(\mathbf{r},\boldsymbol{\rho},\omega)]$$
$$=\iiint_{\mathcal{V}}d\rho_xd\rho_yd\rho_z\left(\frac{k_0^2}{4\pi^2}\right)^2\iiiint_{-\infty}^{+\infty}d^2\boldsymbol{\kappa}_\parallel d^2\boldsymbol{\kappa}'_\parallel\mathrm{Tr}[\hat{\mathbf{G}}^*(\kappa_x,\kappa_y;\omega|z,\rho_z)\hat{\mathbf{G}}(\kappa'_x,\kappa'_y;\omega|z,\rho_z)]e^{ik_0\left[(\kappa'_x-\kappa_x)(x-\rho_x)+(\kappa'_y-\kappa_y)(y-\rho_y)\right]}$$
$$=\frac{k_0^2}{4\pi^2}\int_{-\infty}^{0}d\rho_z\iint_{-\infty}^{+\infty}d^2\boldsymbol{\kappa}_\parallel\mathrm{Tr}[\hat{\mathbf{G}}^*(\kappa_x,\kappa_y;\omega|z,\rho_z)\hat{\mathbf{G}}(\kappa_x,\kappa_y;\omega|z,\rho_z)]$$
$$=\frac{k_0^2}{4\pi^2}\int_{-\infty}^{0}d\rho_z\iint_{-\infty}^{+\infty}d\kappa_xd\kappa_y\left\|\hat{\mathbf{G}}(\kappa_x,\kappa_y;\omega|z,\rho_z)\right\|^2_{\mathcal{F}},$$

where the subscript $\mathcal{F}$ stands for the so-called *Frobenius norm*, defined as:

$$||\hat{\mathbf{A}}||_{\mathcal{F}} = \sqrt{\sum_{i,j} |a_{ij}|^2} = \sqrt{\mathrm{Tr}[\hat{\mathbf{A}}^\dagger \cdot \hat{\mathbf{A}}]}. \tag{S.VI.16}$$

Thus:

$$\mathcal{G}_{0,0}(\mathbf{r},\omega) = \frac{k_0^2}{2\pi} \int_{-\infty}^{0} d\rho_z \int_{0}^{+\infty} d\kappa_R \kappa_R \left\| \hat{\mathbf{G}}^{(\uparrow/\uparrow)}_{z\leftarrow\rho_z}(\kappa_R,\kappa_\varphi;\omega|z,\rho_z) \right\|^2_{\mathcal{F}}, \tag{S.VI.17}$$

where, in accordance with the aforementioned notation, we have set the correspondence $\hat{\mathbf{G}}(\kappa_x,\kappa_y;\omega|z,\rho_z) \to 2\pi\hat{\mathbf{G}}^{(\uparrow/\uparrow)}_{z\leftarrow\rho_z}(\kappa_R,\kappa_\varphi;\omega|z,\rho_z)$, just by making a change of variables from Cartesian to cylindrical coordinates, thus leading to the factor 2π which results from the integration over the angles κ_φ.[‡] We have also included the superscript "$(\uparrow/\uparrow)$" as well as the subscript "$z \leftarrow \rho_z$" to indicate the polarization as well as the coordinates associated to the involved media. In this way, the explicit expression of the dyadic Green's function in the angular spectrum representation for this case is given by (cf. Refs. [12, 13]):

$$\hat{\mathbf{G}}^{(\uparrow/\uparrow)}_{z\leftarrow\rho_z}(\kappa_R,\kappa_\varphi;\omega|z,\rho_z) = \frac{i}{2}\frac{1}{k_{z,2}}\left\{t^{(\mathrm{s})}_{z\leftarrow\rho_z}(\kappa_R,\kappa_\varphi;\omega)[\hat{\mathbf{s}}\otimes\hat{\mathbf{s}}] + t^{(\mathrm{p})}_{z\leftarrow\rho_z}(\kappa_R,\kappa_\varphi;\omega)\left[\hat{\mathbf{p}}^{\uparrow}_z\otimes\hat{\mathbf{p}}^{\uparrow}_{\rho_z}\right]\right\} e^{ik_0\left[\tilde{k}_{z,1}z - \tilde{k}_{z,2}\rho_z\right]}, \tag{S.VI.18}$$

with $k_{z,2} = k_0\tilde{k}_{z,2} = k_0\sqrt{\varepsilon(\omega)}\kappa_{z,2}$, $\hat{\mathbf{s}}$ and $\hat{\mathbf{p}}^{\uparrow}_i$ (with $i = \{z(=1), \rho_z(=2)\}$) being the corresponding polarization-vector basis [see. Eqs. (S.VI.7a) and (S.VI.7b)]:

$$\hat{\mathbf{s}} \equiv \left(\sin\kappa_\varphi, -\cos\kappa_\varphi, 0\right), \tag{S.VI.19a}$$

$$\hat{\mathbf{p}}^{\uparrow}_i \equiv \left(-\kappa_{z,i}\cos\kappa_\varphi, -\kappa_{z,i}\sin\kappa_\varphi, \kappa_R/\sqrt{\varepsilon_i(\omega)}\right), \tag{S.VI.19b}$$

and, $t^{(\mathrm{s})}_{z\leftarrow\rho_z}(\kappa_R,\kappa_\varphi;\omega)$ and $t^{(\mathrm{p})}_{z\leftarrow\rho_z}(\kappa_R,\kappa_\varphi;\omega)$, the *Fresnel transmission coefficients*:

$$t^{(\mathrm{s})}_{z\leftarrow\rho_z}(\kappa_R,\kappa_\varphi;\omega) \equiv \frac{2\varepsilon(\omega)\kappa_{z,2}}{\sqrt{\varepsilon(\omega)}\kappa_{z,1} + \varepsilon(\omega)\kappa_{z,2}}, \tag{S.VI.20a}$$

$$t^{(\mathrm{p})}_{z\leftarrow\rho_z}(\kappa_R,\kappa_\varphi;\omega) \equiv \frac{2\varepsilon(\omega)\kappa_{z,2}}{\varepsilon(\omega)\kappa_{z,1} + \sqrt{\varepsilon(\omega)}\kappa_{z,2}}. \tag{S.VI.20b}$$

Then, inserting Eqs. (S.VI.19a)–(S.VI.20b) into (S.VI.18), the dyadic Green's function reads as:

$$\hat{\mathbf{G}}^{(\uparrow/\uparrow)}_{z\leftarrow\rho_z}(\kappa_R,\kappa_\varphi;\omega|z,\rho_z) = \frac{i}{2}\frac{1}{k_0\tilde{k}_{z,2}}\left[t^{(\mathrm{s})}_{z\leftarrow\rho_z}\hat{\mathbf{g}}^{(\mathrm{s})}_{z\leftarrow\rho_z} + t^{(\mathrm{p})}_{z\leftarrow\rho_z}\hat{\mathbf{g}}^{(\mathrm{p})(\uparrow/\uparrow)}_{z\leftarrow\rho_z}\right]\Gamma_{z\leftarrow\rho_z}, \tag{S.VI.21}$$

with

$$\hat{\mathbf{g}}^{(\mathrm{s})}_{z\leftarrow\rho_z} \equiv \hat{\mathbf{s}}\otimes\hat{\mathbf{s}} = \begin{bmatrix} \sin^2\kappa_\varphi & -\cos\kappa_\varphi\sin\kappa_\varphi & 0 \\ -\cos\kappa_\varphi\sin\kappa_\varphi & \cos^2\kappa_\varphi & 0 \\ 0 & 0 & 0 \end{bmatrix}, \tag{S.VI.22a}$$

$$\hat{\mathbf{g}}^{(\mathrm{p})(\uparrow/\uparrow)}_{z\leftarrow\rho_z} \equiv \hat{\mathbf{p}}^{\uparrow}_z\otimes\hat{\mathbf{p}}^{\uparrow}_{\rho_z} = \begin{bmatrix} \kappa_{z,1}\kappa_{z,2}\cos^2\kappa_\varphi & \kappa_{z,1}\kappa_{z,2}\cos\kappa_\varphi\sin\kappa_\varphi & -\frac{\kappa_{z,1}\kappa_R\cos\kappa_\varphi}{\sqrt{\varepsilon(\omega)}} \\ \kappa_{z,1}\kappa_{z,2}\cos\kappa_\varphi\sin\kappa_\varphi & \kappa_{z,1}\kappa_{z,2}\sin^2\kappa_\varphi & -\frac{\kappa_{z,1}\kappa_R\sin\kappa_\varphi}{\sqrt{\varepsilon(\omega)}} \\ -\kappa_{z,2}\kappa_R\cos\kappa_\varphi & -\kappa_{z,2}\kappa_R\sin\kappa_\varphi & \frac{\kappa_R^2}{\sqrt{\varepsilon(\omega)}} \end{bmatrix}, \tag{S.VI.22b}$$

$$\Gamma_{z\leftarrow\rho_z} = e^{ik_0\left[\tilde{k}_{z,1}z - \tilde{k}_{z,2}\rho_z\right]}. \tag{S.VI.22c}$$

[‡] The change to cylindrical coordinates in the wavevector is so that $\kappa_x = \kappa_R\cos\kappa_\varphi$, $\kappa_y = \kappa_R\sin\kappa_\varphi$, and $\kappa_z = \kappa_z$, with $\kappa_x^2 + \kappa_y^2 = \kappa_R^2$.

Thus, putting it all together it follows that:

$$\left\|\hat{\mathbf{G}}^{(\uparrow/\uparrow)}_{z\leftarrow\rho_z}(\kappa_R,\kappa_\varphi;\omega|z,\rho_z)\right\|^2_{\mathcal{F}} = \frac{1}{4}\frac{1}{k_0^2|\tilde{k}_{z,2}|^2}\left[\left|t^{(\mathrm{s})}_{z\leftarrow\rho_z}\right|^2\left\|\hat{\mathbf{g}}^{(\mathrm{s})}_{z\leftarrow\rho_z}\right\|^2_{\mathcal{F}} + \left|t^{(\mathrm{p})}_{z\leftarrow\rho_z}\right|^2\left\|\hat{\mathbf{g}}^{(\mathrm{p})(\uparrow/\uparrow)}_{z\leftarrow\rho_z}\right\|^2_{\mathcal{F}}\right]|\Gamma_{z\leftarrow\rho_z}|^2, \quad \text{(S.VI.23)}$$

with

$$\left\|\hat{\mathbf{g}}^{(\mathrm{s})}_{z\leftarrow\rho_z}\right\|^2_{\mathcal{F}} = (\hat{\mathbf{s}}\cdot\hat{\mathbf{s}}^*)(\hat{\mathbf{s}}\cdot\hat{\mathbf{s}}^*) = 1,$$

$$\left\|\hat{\mathbf{g}}^{(\mathrm{p})(\uparrow/\uparrow)}_{z\leftarrow\rho_z}\right\|^2_{\mathcal{F}} = \left(\hat{\mathbf{p}}^{\uparrow}_z\cdot[\hat{\mathbf{p}}^{\uparrow}_z]^*\right)\left(\hat{\mathbf{p}}^{\uparrow}_{\rho_z}\cdot[\hat{\mathbf{p}}^{\uparrow}_{\rho_z}]^*\right) = \frac{1}{|\varepsilon(\omega)|}\left\{|\tilde{k}_{z,1}|^2|\tilde{k}_{z,2}|^2 + \kappa_R^2\left[|\tilde{k}_{z,2}|^2 + |\tilde{k}_{z,1}|^2 + \kappa_R^2\right]\right\},$$

$$|\Gamma_{z\leftarrow\rho_z}|^2 = \Gamma^*_{z\leftarrow\rho_z}\Gamma_{z\leftarrow\rho_z} = e^{-ik_0\left[\tilde{k}^*_{z,1}z-\tilde{k}^*_{z,2}\rho_z\right]}e^{ik_0\left[\tilde{k}_{z,1}z-\tilde{k}_{z,2}\rho_z\right]} = e^{-2k_0\mathrm{Im}[\tilde{k}_{z,1}]z}e^{2k_0\mathrm{Im}[\tilde{k}_{z,2}]\rho_z},$$

so that:

$$\mathcal{G}_{0,0}(\mathbf{r},\omega) = \frac{k_0^2}{2\pi}\int_{-\infty}^{0}d\rho_z\int_{0}^{+\infty}d\kappa_R\kappa_R\left\{\frac{1}{4}\frac{1}{k_0^2|\tilde{k}_{z,2}|^2}\left[\left|t^{(\mathrm{s})}_{z\leftarrow\rho_z}\right|^2\left\|\hat{\mathbf{g}}^{(\mathrm{s})}_{z\leftarrow\rho_z}\right\|^2_{\mathcal{F}} + \left|t^{(\mathrm{p})}_{z\leftarrow\rho_z}\right|^2\left\|\hat{\mathbf{g}}^{(\mathrm{p})(\uparrow/\uparrow)}_{z\leftarrow\rho_z}\right\|^2_{\mathcal{F}}\right]|\Gamma_{z\leftarrow\rho_z}|^2\right\}$$

$$= \frac{1}{16\pi k_0}\int_{0}^{+\infty}d\kappa_R\frac{\kappa_R}{|\tilde{k}_{z,2}|^2}\left[\left|t^{(\mathrm{s})}_{z\leftarrow\rho_z}\right|^2\left\|\hat{\mathbf{g}}^{(\mathrm{s})}_{z\leftarrow\rho_z}\right\|^2_{\mathcal{F}} + \left|t^{(\mathrm{p})}_{z\leftarrow\rho_z}\right|^2\left\|\hat{\mathbf{g}}^{(\mathrm{p})(\uparrow/\uparrow)}_{z\leftarrow\rho_z}\right\|^2_{\mathcal{F}}\right]\frac{e^{-2k_0\mathrm{Im}[\tilde{k}_{z,1}]z}}{\mathrm{Im}[\tilde{k}_{z,2}]}. \quad \text{(S.VI.24)}$$

Finally:

$$\mathcal{S}_0(\mathbf{r};\omega;T) = \frac{1}{4\varepsilon_0}\frac{\omega^2}{c^3}\mathrm{Im}[\varepsilon(\omega)]\hbar\omega\Theta(\omega,T)\left[\int_0^{+\infty}d\kappa_R\frac{\kappa_R}{|\tilde{k}_{z,2}|^2}\left[\left\|\hat{\mathcal{G}}^{(\mathrm{s})}_{0,0}\right\|^2_{\mathcal{F}} + \left\|\hat{\mathcal{G}}^{(\mathrm{p})}_{0,0}\right\|^2_{\mathcal{F}}\right]\frac{e^{-2k_0\mathrm{Im}[\tilde{k}_{z,1}]z}}{\mathrm{Im}[\tilde{k}_{z,2}]}\right], \quad \text{(S.VI.25)}$$

with

$$\left\|\hat{\mathcal{G}}^{(\mathrm{s})}_{0,0}\right\|^2_{\mathcal{F}} \equiv \left|t^{(\mathrm{s})}_{z\leftarrow\rho_z}\right|^2\left\|\hat{\mathbf{g}}^{(\mathrm{s})}_{z\leftarrow\rho_z}\right\|^2_{\mathcal{F}} = \left|t^{(\mathrm{s})}\right|^2,$$

$$\left\|\hat{\mathcal{G}}^{(\mathrm{p})}_{0,0}\right\|^2_{\mathcal{F}} \equiv \left|t^{(\mathrm{p})}_{z\leftarrow\rho_z}\right|^2\left\|\hat{\mathbf{g}}^{(\mathrm{p})(\uparrow/\uparrow)}_{z\leftarrow\rho_z}\right\|^2_{\mathcal{F}} = \left|t^{(\mathrm{p})}\right|^2\frac{1}{|\varepsilon(\omega)|}\left\{|\tilde{k}_{z,1}|^2|\tilde{k}_{z,2}|^2 + \kappa_R^2\left[|\tilde{k}_{z,2}|^2 + |\tilde{k}_{z,1}|^2 + \kappa_R^2\right]\right\}.$$

Second-order of thermal emission

As shown above, the zeroth-order correlation, $\mathcal{S}_0(\mathbf{r};\omega;T)$, leads to the ground spectra in which the time-modulation does not enter into play. In this sense, the first-order correlation $\mathcal{S}_1(\mathbf{r};\omega;T)$ should provide for the first insights about the role of time-modulation into the system, e.g., showing up some non-local features described by means of the spatial integral. Notwithstanding, it can be observed that, at such an order, the time-modulation, characterized by means of the Laplace transform of the time-dependent susceptibility, does not account for effective frequency correlations. Essentially, this is because, in the calculation of thermal emission spectra, one is actually interested in correlations evaluated at a given frequency. For the first-order, only frequencies that are separated by the material's modulating frequency can correlate, so that, according to the result shown in Eq. (S.V.2), currents at the same frequency would not contribute effectively to the first-order spectrum. Hence, the first term in the development yielding a non-null contribution due to the time-modulation would be the second one. As pointed out previously, this term can be expressed as a sum of three contributions: $\mathcal{S}_2(\mathbf{r};\omega;T) = \mathcal{S}_{2,0}(\mathbf{r};\omega;T) + \mathcal{S}_{0,2}(\mathbf{r};\omega;T) + \mathcal{S}_{1,1}(\mathbf{r};\omega;T)$, each of them displaying different behaviors depending on the specific currents involved. In this regard, correlations $\mathcal{S}_{2,0}(\mathbf{r};\omega;T) + \mathcal{S}_{0,2}(\mathbf{r};\omega;T)$ will connect different frequencies appearing at different locations of the system, thus introducing a kind of non-local behavior that preserves the distribution (and so, the average energy) of the oscillators. On the other hand, the term $\mathcal{S}_{1,1}(\mathbf{r};\omega;T)$ owns the additional feature of affecting to the photon distribution, producing a frequency-shift related to the frequency of modulation. In the following, we shall explicitly look into each of these contributions for the particular case pointed out above.

Looking at the results given in the previous section, the second-order correlations of the electric-field are given by:

$$\begin{aligned}\mathcal{S}_{0,2}(\mathbf{r};\omega;T) &= \langle[\hat{\mathbf{E}}_0^{(+)}(\mathbf{r};\omega)]^\dagger\cdot\hat{\mathbf{E}}_2^{(+)}(\mathbf{r};\omega)\rangle_{\text{th}} \\ &= \omega^2\mu_0^2\iint\limits_{\mathcal{V}} d^3\boldsymbol{\rho}d^3\boldsymbol{\rho}'\text{Tr}[\mathbf{G}^*(\mathbf{r},\boldsymbol{\rho},\omega)\mathbf{G}(\mathbf{r},\boldsymbol{\rho}',\omega)]\,\langle\hat{\mathbf{j}}_0^\dagger(\boldsymbol{\rho};\omega)\cdot\hat{\mathbf{j}}_2(\boldsymbol{\rho}';\omega)\rangle_{\text{th}},\end{aligned} \tag{S.VI.26a}$$

$$\begin{aligned}\mathcal{S}_{1,1}(\mathbf{r};\omega;T) &= \langle[\hat{\mathbf{E}}_1^{(+)}(\mathbf{r};\omega)]^\dagger\cdot\hat{\mathbf{E}}_1^{(+)}(\mathbf{r};\omega)\rangle_{\text{th}} \\ &= \omega^2\mu_0^2\iint\limits_{\mathcal{V}} d^3\boldsymbol{\rho}d^3\boldsymbol{\rho}'\text{Tr}[\mathbf{G}^*(\mathbf{r},\boldsymbol{\rho},\omega)\mathbf{G}(\mathbf{r},\boldsymbol{\rho}',\omega)]\,\langle\hat{\mathbf{j}}_1^\dagger(\boldsymbol{\rho};\omega)\cdot\hat{\mathbf{j}}_1(\boldsymbol{\rho}';\omega)\rangle_{\text{th}},\end{aligned} \tag{S.VI.26b}$$

noticing that $\mathcal{S}_{2,0}(\mathbf{r};\omega;T) = \langle[\hat{\mathbf{E}}_2^{(+)}(\mathbf{r};\omega)]^\dagger\cdot\hat{\mathbf{E}}_0^{(+)}(\mathbf{r};\omega)\rangle_{\text{th}} = [\langle[\hat{\mathbf{E}}_0^{(+)}(\mathbf{r};\omega)]^\dagger\cdot\hat{\mathbf{E}}_2^{(+)}(\mathbf{r};\omega)\rangle_{\text{th}}]^\dagger = [\mathcal{S}_{0,2}(\mathbf{r};\omega;T)]^\dagger$. Likewise, the current density correlations involved in the above expressions are given by:

$$\langle\hat{\mathbf{j}}_0^\dagger(\boldsymbol{\rho};\omega)\cdot\hat{\mathbf{j}}_2(\boldsymbol{\rho}';\omega)\rangle_{\text{th}} = \frac{\omega\mu_0}{\pi}\int\limits_{\mathcal{V}} d^3\boldsymbol{\rho}''\left[\int\limits_{-\infty}^{+\infty} d\omega''\omega''\Delta\tilde{\chi}(\boldsymbol{\rho}',\omega-\omega'')\text{Tr}[\mathbf{G}(\boldsymbol{\rho}',\boldsymbol{\rho}'',\omega'')]\,\langle\hat{\mathbf{j}}_0^\dagger(\boldsymbol{\rho};\omega)\cdot\hat{\mathbf{j}}_1(\boldsymbol{\rho}'';\omega'')\rangle_{\text{th}}\right],$$

$$\langle\hat{\mathbf{j}}_0^\dagger(\boldsymbol{\rho};\omega)\cdot\hat{\mathbf{j}}_1(\boldsymbol{\rho}'';\omega'')\rangle_{\text{th}} = \frac{\omega''\mu_0}{\pi}\int\limits_{\mathcal{V}} d^3\boldsymbol{\rho}'''\left[\int\limits_{-\infty}^{+\infty} d\omega'''\omega'''\Delta\tilde{\chi}(\boldsymbol{\rho}'',\omega''-\omega''')\text{Tr}[\mathbf{G}(\boldsymbol{\rho}'',\boldsymbol{\rho}''',\omega''')]\,\langle\hat{\mathbf{j}}_0^\dagger(\boldsymbol{\rho};\omega)\cdot\hat{\mathbf{j}}_0(\boldsymbol{\rho}''';\omega''')\rangle_{\text{th}}\right],$$

$$\begin{aligned}\langle\hat{\mathbf{j}}_1^\dagger(\boldsymbol{\rho};\omega)\cdot\hat{\mathbf{j}}_1(\boldsymbol{\rho}';\omega)\rangle_{\text{th}} = \frac{\omega^2\mu_0^2}{\pi^2}\iint\limits_{\mathcal{V}} d^3\tilde{\boldsymbol{\rho}}d^3\tilde{\boldsymbol{\rho}}'\iint\limits_{-\infty}^{+\infty} d\tilde{\omega}d\tilde{\omega}'\tilde{\omega}\tilde{\omega}'\Delta\tilde{\chi}^*(\boldsymbol{\rho},\omega-\tilde{\omega})\Delta\tilde{\chi}(\boldsymbol{\rho}',\omega-\tilde{\omega}') \\ \Big[\text{Tr}[\mathbf{G}^*(\boldsymbol{\rho},\tilde{\boldsymbol{\rho}},\tilde{\omega})\mathbf{G}(\boldsymbol{\rho}',\tilde{\boldsymbol{\rho}}',\tilde{\omega}')]\,\langle\hat{\mathbf{j}}_0^\dagger(\tilde{\boldsymbol{\rho}};\tilde{\omega})\cdot\hat{\mathbf{j}}_0(\tilde{\boldsymbol{\rho}}';\tilde{\omega}')\rangle_{\text{th}} + \text{Tr}[\mathbf{G}(\boldsymbol{\rho},\tilde{\boldsymbol{\rho}},\tilde{\omega})\mathbf{G}^*(\boldsymbol{\rho}',\tilde{\boldsymbol{\rho}}',\tilde{\omega}')]\,\langle\hat{\mathbf{j}}_0(\tilde{\boldsymbol{\rho}};\tilde{\omega})\cdot\hat{\mathbf{j}}_0^\dagger(\tilde{\boldsymbol{\rho}}';\tilde{\omega}')\rangle_{\text{th}}\Big].\end{aligned}$$

For simplicity, as well as convenience, henceforth we shall particularize to time-harmonic modulations of the form:

$$\Delta\tilde{\chi}(\mathbf{r},t) = \varepsilon_0\delta\chi\Delta\tilde{\chi}(t) = \varepsilon_0\delta\chi\sin(\Omega t) = \frac{\varepsilon_0\delta\chi}{2i}\left[e^{i\Omega t} - e^{-i\Omega t}\right], \tag{S.VI.27}$$

and whose Laplace transform is simply given by:

$$\begin{aligned}\Delta\tilde{\chi}(\mathbf{r},\omega) = \varepsilon_0\delta\chi\Delta\tilde{\chi}(\omega) = \varepsilon_0\delta\chi\mathcal{L}_\omega[\Delta\tilde{\chi}(t)] &= \frac{\varepsilon_0\delta\chi}{2i}\left\{\mathcal{L}_\omega\left[e^{i\Omega t}\right] - \mathcal{L}_\omega\left[e^{-i\Omega t}\right]\right\} \\ &= \frac{\varepsilon_0\delta\chi}{2i}\left\{\frac{i}{\omega+\Omega+i0^+} - \frac{i}{\omega-\Omega+i0^+}\right\} \\ &= \frac{\varepsilon_0\delta\chi\pi}{2i}\left(\delta[\omega+\Omega] - \delta[\omega-\Omega] + i\left\{\frac{1}{\pi}\mathcal{P}\left[\frac{1}{\omega+\Omega}\right] - \frac{1}{\pi}\mathcal{P}\left[\frac{1}{\omega-\Omega}\right]\right\}\right) \\ &\to \frac{\varepsilon_0\delta\chi\pi}{i}\left(\delta[\omega+\Omega] - \delta[\omega-\Omega]\right).\end{aligned} \tag{S.VI.28}$$

Therefore,

$$\begin{aligned}\langle\hat{\mathbf{j}}_0^\dagger(\boldsymbol{\rho};\omega)\cdot\hat{\mathbf{j}}_1(\boldsymbol{\rho}'';\omega'')\rangle_{\text{th}} &= \frac{\delta\chi\omega''}{ic^2}\int\limits_{\mathcal{V}} d^3\boldsymbol{\rho}'''(\omega''+\Omega)\text{Tr}[\mathbf{G}(\boldsymbol{\rho}'',\boldsymbol{\rho}''',\omega''+\Omega)]\,\langle\hat{\mathbf{j}}_0^\dagger(\boldsymbol{\rho};\omega)\cdot\hat{\mathbf{j}}_0(\boldsymbol{\rho}''';\omega''+\Omega)\rangle_{\text{th}} \\ &- \frac{\delta\chi\omega''}{ic^2}\int\limits_{\mathcal{V}} d^3\boldsymbol{\rho}'''(\omega''-\Omega)\text{Tr}[\mathbf{G}(\boldsymbol{\rho}'',\boldsymbol{\rho}''',\omega''-\Omega)]\,\langle\hat{\mathbf{j}}_0^\dagger(\boldsymbol{\rho};\omega)\cdot\hat{\mathbf{j}}_0(\boldsymbol{\rho}''';\omega''-\Omega)\rangle_{\text{th}} \\ &= \frac{4\pi\varepsilon_0\delta\chi}{i}\text{Im}[\varepsilon(\omega)]\hbar\omega\Theta(\omega,T)\frac{\omega''}{c^2}(\omega''+\Omega)^2\text{Tr}[\mathbf{G}(\boldsymbol{\rho}'',\boldsymbol{\rho},\omega''+\Omega)]\delta[\omega-(\omega''+\Omega)] \\ &- \frac{4\pi\varepsilon_0\delta\chi}{i}\text{Im}[\varepsilon(\omega)]\hbar\omega\Theta(\omega,T)\frac{\omega''}{c^2}(\omega''-\Omega)^2\text{Tr}[\mathbf{G}(\boldsymbol{\rho}'',\boldsymbol{\rho},\omega''-\Omega)]\delta[\omega-(\omega''-\Omega)],\end{aligned}$$

$$
\begin{aligned}
\langle \hat{\mathbf{j}}_0^\dagger(\boldsymbol{\rho};\omega)\cdot\hat{\mathbf{j}}_2(\boldsymbol{\rho}';\omega)\rangle_{\rm th} &= \frac{\delta\chi^2\omega}{ic^2}\int\limits_{\mathcal{V}} d^3\boldsymbol{\rho}''(\omega+\Omega){\rm Tr}[\mathbf{G}(\boldsymbol{\rho}',\boldsymbol{\rho}'',\omega+\Omega)]\,\langle\hat{\mathbf{j}}_0^\dagger(\boldsymbol{\rho};\omega)\cdot\hat{\mathbf{j}}_1(\boldsymbol{\rho}'';\omega+\Omega)\rangle_{\rm th}\\
&\quad-\frac{\delta\chi^2\omega}{ic^2}\int\limits_{\mathcal{V}} d^3\boldsymbol{\rho}''(\omega-\Omega){\rm Tr}[\mathbf{G}(\boldsymbol{\rho}',\boldsymbol{\rho}'',\omega-\Omega)]\,\langle\hat{\mathbf{j}}_0^\dagger(\boldsymbol{\rho};\omega)\cdot\hat{\mathbf{j}}_1(\boldsymbol{\rho}'';\omega-\Omega)\rangle_{\rm th}\\
&=4\pi\varepsilon_0\delta\chi^2{\rm Im}[\varepsilon(\omega)]\hbar\omega\Theta(\omega,T)\frac{(\omega+\Omega)^2\omega^3}{c^4}\left[\int\limits_{\mathcal{V}} d^3\boldsymbol{\rho}''{\rm Tr}[\mathbf{G}(\boldsymbol{\rho}',\boldsymbol{\rho}'',\omega+\Omega)\mathbf{G}(\boldsymbol{\rho}'',\boldsymbol{\rho},\omega)]\right]\\
&\quad+4\pi\varepsilon_0\delta\chi^2{\rm Im}[\varepsilon(\omega)]\hbar\omega\Theta(\omega,T)\frac{(\omega-\Omega)^2\omega^3}{c^4}\left[\int\limits_{\mathcal{V}} d^3\boldsymbol{\rho}''{\rm Tr}[\mathbf{G}(\boldsymbol{\rho}',\boldsymbol{\rho}'',\omega-\Omega)\mathbf{G}(\boldsymbol{\rho}'',\boldsymbol{\rho},\omega)]\right],
\end{aligned}
$$

thereby leading to:

$$
\mathcal{S}_{0,2}(\mathbf{r};\omega;T)=\frac{4\pi\delta\chi^2}{\varepsilon_0}\frac{\omega^5}{c^8}{\rm Im}[\varepsilon(\omega)]\hbar\omega\Theta(\omega,T)\left\{\left(\omega^+\right)^2\bar{\bar{\mathcal{G}}}_{0,2}^{(\omega^+)}+\left(\omega^-\right)^2\bar{\bar{\mathcal{G}}}_{0,2}^{(\omega^-)}\right\}, \tag{S.VI.29}
$$

with

$$
\bar{\bar{\mathcal{G}}}_{0,2}^{(\omega^\pm)}\equiv\iiint\limits_{\mathcal{V}} d^3\boldsymbol{\rho}d^3\boldsymbol{\rho}'d^3\boldsymbol{\rho}''{\rm Tr}[\mathbf{G}^*(\mathbf{r},\boldsymbol{\rho},\omega)\mathbf{G}(\mathbf{r},\boldsymbol{\rho}',\omega)\mathbf{G}(\boldsymbol{\rho}',\boldsymbol{\rho}'',\omega^\pm)\mathbf{G}(\boldsymbol{\rho}'',\boldsymbol{\rho},\omega)], \tag{S.VI.30}
$$

and where it has been defined $\omega^\pm\equiv\omega\pm\Omega$.

Similarly,

$$
\begin{aligned}
\langle \hat{\mathbf{j}}_1^\dagger(\boldsymbol{\rho};\omega)\cdot\hat{\mathbf{j}}_1(\boldsymbol{\rho}';\omega)\rangle_{\rm th} &= 4\pi\varepsilon_0\delta\chi^2\frac{\omega^2}{c^4}\left\{(\omega^+)^3{\rm Im}\big[\varepsilon(\omega^+)\big]\hbar\omega^+\Theta(\omega^+,T)\int\limits_{\mathcal{V}} d^3\boldsymbol{\rho}''{\rm Tr}[\mathbf{G}^*(\boldsymbol{\rho},\boldsymbol{\rho}'',\omega^+)\mathbf{G}(\boldsymbol{\rho}',\boldsymbol{\rho}'',\omega^+)]\right\}\\
&\quad+4\pi\varepsilon_0\delta\chi^2\frac{\omega^2}{c^4}\left\{(\omega^+)^3{\rm Im}\big[\varepsilon(\omega^+)\big]\hbar\omega^+\left[\Theta(\omega^+,T)+1\right]\int\limits_{\mathcal{V}} d^3\boldsymbol{\rho}''{\rm Tr}[\mathbf{G}(\boldsymbol{\rho},\boldsymbol{\rho}'',\omega^+)\mathbf{G}^*(\boldsymbol{\rho}',\boldsymbol{\rho}'',\omega^+)]\right\}\\
&\quad+4\pi\varepsilon_0\delta\chi^2\frac{\omega^2}{c^4}\left\{(\omega^-)^3{\rm Im}\big[\varepsilon(\omega^-)\big]\hbar\omega^-\Theta(\omega^-,T)\int\limits_{\mathcal{V}} d^3\boldsymbol{\rho}''{\rm Tr}[\mathbf{G}^*(\boldsymbol{\rho},\boldsymbol{\rho}'',\omega^-)\mathbf{G}(\boldsymbol{\rho}',\boldsymbol{\rho}'',\omega^-)]\right\}\\
&\quad+4\pi\varepsilon_0\delta\chi^2\frac{\omega^2}{c^4}\left\{(\omega^-)^3{\rm Im}\big[\varepsilon(\omega^-)\big]\hbar\omega^-\left[\Theta(\omega^-,T)+1\right]\int\limits_{\mathcal{V}} d^3\boldsymbol{\rho}''{\rm Tr}[\mathbf{G}(\boldsymbol{\rho},\boldsymbol{\rho}'',\omega^-)\mathbf{G}^*(\boldsymbol{\rho}',\boldsymbol{\rho}'',\omega^-)]\right\},
\end{aligned}
$$

so that,

$$
\mathcal{S}_{1,1}(\mathbf{r};\omega;T)=\mathcal{S}_{1,1}^{(\omega^+)}(\mathbf{r};\omega;T)+\mathcal{S}_{1,1}^{(\omega^-)}(\mathbf{r};\omega;T), \tag{S.VI.31}
$$

where

$$
\mathcal{S}_{1,1}^{(\omega^\pm)}(\mathbf{r};\omega;T)=\frac{4\pi\delta\chi^2}{\varepsilon_0}\frac{\omega^5}{c^8}{\rm Im}\big[\varepsilon(\omega^\pm)\big]\hbar\omega^\pm(\omega^\pm)^2(1+\tilde{\Omega})\left\{\Theta(\omega^\pm,T)\bar{\mathcal{G}}_{1,1}^{(\omega^\pm)}+\left[\Theta(\omega^\pm,T)+1\right]\tilde{\mathcal{G}}_{1,1}^{(\omega^\pm)}\right\}, \tag{S.VI.32}
$$

with $\tilde{\Omega}\equiv\Omega/\omega$, and

$$
\bar{\mathcal{G}}_{1,1}^{(\omega^\pm)}\equiv\iiint\limits_{\mathcal{V}} d^3\boldsymbol{\rho}d^3\boldsymbol{\rho}'d^3\boldsymbol{\rho}''{\rm Tr}[\mathbf{G}^*(\mathbf{r},\boldsymbol{\rho},\omega)\mathbf{G}^*(\boldsymbol{\rho},\boldsymbol{\rho}'',\omega^\pm)\mathbf{G}(\mathbf{r},\boldsymbol{\rho}',\omega)\mathbf{G}(\boldsymbol{\rho}',\boldsymbol{\rho}'',\omega^\pm)], \tag{S.VI.33a}
$$

$$
\tilde{\mathcal{G}}_{1,1}^{(\omega^\pm)}\equiv\iiint\limits_{\mathcal{V}} d^3\boldsymbol{\rho}d^3\boldsymbol{\rho}'d^3\boldsymbol{\rho}''{\rm Tr}[\mathbf{G}^*(\mathbf{r},\boldsymbol{\rho},\omega)\mathbf{G}(\boldsymbol{\rho},\boldsymbol{\rho}'',\omega^\pm)\mathbf{G}(\mathbf{r},\boldsymbol{\rho}',\omega)\mathbf{G}^*(\boldsymbol{\rho}',\boldsymbol{\rho}'',\omega^\pm)]. \tag{S.VI.33b}
$$

Calculation of $\bar{\mathcal{G}}_{1,1}^{(\omega^{\pm})}$:

To carry on with the calculation of $\bar{\mathcal{G}}_{1,1}^{(\omega^{\pm})}$, first of all it is important to ensure that all of the matrices involved are properly arranged:

$$[\hat{E}_{1,\alpha}^{(+)}]^{\dagger}\cdot\hat{E}_{1,\alpha}^{(+)}\propto G_{\alpha,\gamma}^{*}\hat{j}_{1,\gamma}^{\dagger}G_{\alpha,\delta}\hat{j}_{1,\delta}\propto G_{\alpha,\gamma}^{*}G_{\gamma,\beta}^{*}\hat{j}_{0,\beta}^{\dagger}G_{\alpha,\delta}G_{\delta,\zeta}\hat{j}_{0,\zeta}\propto G_{\alpha,\gamma}^{*}G_{\gamma,\beta}^{*}G_{\alpha,\delta}G_{\delta,\zeta}\delta_{\beta,\zeta}\propto G_{\alpha,\gamma}^{*}G_{\gamma,\beta}^{*}G_{\alpha,\delta}G_{\delta,\beta}.$$

In that way it is easy to see that the four matrices can be grouped as:

$$\begin{aligned}\mathbf{G}^{*}(\mathbf{r},\boldsymbol{\rho},\omega)\mathbf{G}^{*}(\boldsymbol{\rho},\boldsymbol{\rho}'',\omega^{\pm})\mathbf{G}(\mathbf{r},\boldsymbol{\rho}',\omega)\mathbf{G}(\boldsymbol{\rho}',\boldsymbol{\rho}'',\omega^{\pm}) &= G_{\alpha,\gamma}^{*}(\mathbf{r},\boldsymbol{\rho},\omega)G_{\gamma,\beta}^{*}(\boldsymbol{\rho},\boldsymbol{\rho}'',\omega^{\pm})G_{\alpha,\delta}(\mathbf{r},\boldsymbol{\rho}',\omega)G_{\delta,\beta}(\boldsymbol{\rho}',\boldsymbol{\rho}'',\omega^{\pm})\\ &= \bar{G}_{\alpha,\beta}^{*}(\mathbf{r},\boldsymbol{\rho},\boldsymbol{\rho}'',\omega,\omega^{\pm})\bar{G}_{\alpha,\beta}(\mathbf{r},\boldsymbol{\rho}',\boldsymbol{\rho}'',\omega,\omega^{\pm}),\end{aligned}$$

where $\bar{G}_{\alpha,\beta}^{*}(\mathbf{r},\boldsymbol{\rho},\boldsymbol{\rho}'',\omega,\omega^{\pm})\equiv G_{\alpha,\gamma}^{*}(\mathbf{r},\boldsymbol{\rho},\omega)G_{\gamma,\beta}^{*}(\boldsymbol{\rho},\boldsymbol{\rho}'',\omega^{\pm})$ and $\bar{G}_{\alpha,\beta}(\mathbf{r},\boldsymbol{\rho}',\boldsymbol{\rho}'',\omega,\omega^{\pm})\equiv G_{\alpha,\delta}(\mathbf{r},\boldsymbol{\rho}',\omega)G_{\delta,\beta}(\boldsymbol{\rho}',\boldsymbol{\rho}'',\omega^{\pm})$, noticing that the summation over γ and δ actually refers to the customary matrix product. Inserting this latter expression into (S.VI.33a), and making use of the expansion given in Eq. (S.VI.1), it follows that:

$$\begin{aligned}\bar{\mathcal{G}}_{1,1}^{(\omega^{\pm})} &= \iiint\limits_{\mathcal{V}} d^{3}\boldsymbol{\rho}d^{3}\boldsymbol{\rho}'d^{3}\boldsymbol{\rho}''\mathrm{Tr}[\mathbf{G}^{*}(\mathbf{r},\boldsymbol{\rho},\omega)\mathbf{G}^{*}(\boldsymbol{\rho},\boldsymbol{\rho}'',\omega^{\pm})\mathbf{G}(\mathbf{r},\boldsymbol{\rho}',\omega)\mathbf{G}(\boldsymbol{\rho}',\boldsymbol{\rho}'',\omega^{\pm})]\\ &= \frac{k_0^2}{4\pi^2}\iiint\limits_{-\infty}^{0} d\rho_z d\rho_z' d\rho_z''\iint\limits_{-\infty}^{+\infty} d^2\boldsymbol{\kappa}_{\parallel}\mathrm{Tr}[\hat{\mathbf{G}}^{*}(\kappa_x,\kappa_y;\omega|z,\rho_z)\hat{\mathbf{G}}^{*}(\kappa_x^{\pm},\kappa_y^{\pm};\omega^{\pm}|\rho_z,\rho_z'')\hat{\mathbf{G}}(\kappa_x,\kappa_y;\omega|z,\rho_z')\hat{\mathbf{G}}(\kappa_x^{\pm},\kappa_y^{\pm};\omega^{\pm}|\rho_z',\rho_z'')],\end{aligned}$$

where $\kappa_i^{\pm}\equiv\kappa_i/(1\pm\tilde{\Omega})$, with $i=\{x,y\}$[§]. Hence:

$$\bar{\mathcal{G}}_{1,1}^{(\omega^{\pm})}=\frac{k_0^2}{4\pi^2}\iiint\limits_{-\infty}^{0} d\rho_z d\rho_z' d\rho_z''\int\limits_{0}^{+\infty} d\kappa_R\kappa_R\int\limits_{0}^{2\pi} d\kappa_{\varphi}\left\|\left[\hat{\bar{\mathbf{G}}}_{z\leftarrow\rho_z\leftarrow\rho_z''}^{(\omega^{\pm})[(\uparrow/\uparrow)(\updownarrow/\updownarrow)]}\right]^{*}\hat{\bar{\mathbf{G}}}_{z\leftarrow\rho_z'\leftarrow\rho_z''}^{(\omega^{\pm})[(\uparrow/\uparrow)(\updownarrow/\updownarrow)]}\right\|_{\mathcal{F}},\tag{S.VI.34}$$

where

$$\left[\hat{\bar{\mathbf{G}}}_{z\leftarrow\rho_z\leftarrow\rho_z''}^{(\omega^{\pm})[(\uparrow/\uparrow)(\updownarrow/\updownarrow)]}\right]^{*}\equiv\left[\hat{\bar{\mathbf{G}}}_{z\leftarrow\rho_z\leftarrow\rho_z''}^{(\omega^{\pm})[(\updownarrow/\updownarrow)]}\right]^{*}=\left[\hat{\mathbf{G}}^{(\uparrow/\uparrow)}(\kappa_x,\kappa_y;\omega|z,\rho_z)\right]^{*}\cdot\left[\hat{\mathbf{G}}^{(\updownarrow/\updownarrow)}(\kappa_x^{\pm},\kappa_y^{\pm};\omega^{\pm}|\rho_z,\rho_z'')\right]^{*},\tag{S.VI.35a}$$

$$\hat{\bar{\mathbf{G}}}_{z\leftarrow\rho_z'\leftarrow\rho_z''}^{(\omega^{\pm})[(\uparrow/\uparrow)(\updownarrow/\updownarrow)]}\equiv\hat{\bar{\mathbf{G}}}_{z\leftarrow\rho_z'\leftarrow\rho_z''}^{(\omega^{\pm})[(\updownarrow/\updownarrow)]}=\hat{\mathbf{G}}^{(\uparrow/\uparrow)}(\kappa_x,\kappa_y;\omega|z,\rho_z')\cdot\hat{\mathbf{G}}^{(\updownarrow/\updownarrow)}(\kappa_x^{\pm},\kappa_y^{\pm};\omega^{\pm}|\rho_z',\rho_z'').\tag{S.VI.35b}$$

There are some similarities between these results and those obtained for the zeroth-order contribution [compare Eq. (S.VI.34) with Eq. (S.VI.17)]. However, in this case the matrices in the product of Eq. (S.VI.34) are not the same, thus resulting not in the *Frobenius norm*, but in the so-called *Frobenius inner product*. Furthermore, in each of the products given in Eqs. (S.VI.35a) and (S.VI.35b), the second matrix involves variables lying in the same medium. So, whilst the first matrix will be of the form of Eq. (S.VI.5), the second one will be like that given in Eq. (S.VI.10). At any rate, one can proceed just by following the notation sketched out in Eqs. (S.VI.5)–(S.VI.13):

$$\begin{aligned}\left[\hat{\bar{\mathbf{G}}}_{z\leftarrow\rho_z\leftarrow\rho_z''}^{(\omega^{\pm})[(\updownarrow/\updownarrow)]}\right]^{*} &= -\frac{1}{4}\frac{1}{k_{z,2}^{*}k_{z,2}^{\pm*}}\left[t^{(\mathrm{s})}\hat{\mathbf{g}}^{(\mathrm{s})}+t^{(\mathrm{p})}\left(\hat{\mathbf{p}}_{\rho_z}^{\uparrow}\cdot\hat{\mathbf{p}}_{\rho_z}^{(\omega^{\pm})\updownarrow}\right)\left(\hat{\mathbf{p}}_{z}^{\uparrow}\otimes\hat{\mathbf{p}}_{\rho_z''}^{(\omega^{\pm})\updownarrow}\right)\right]^{*}e^{-i\left[k_{z,1}^{*}z-k_{z,2}^{*}\rho_z\right]}e^{-ik_{z,2}^{\pm*}|\rho_z-\rho_z''|}\\ &\quad-\frac{1}{4}\frac{1}{k_{z,2}^{*}k_{z,2}^{\pm*}}\left[t^{(\mathrm{s})}r^{(\mathrm{s})(\omega^{\pm})}\hat{\mathbf{g}}^{(\mathrm{s})}+t^{(\mathrm{p})}r^{(\mathrm{p})(\omega^{\pm})}\left(\hat{\mathbf{p}}_{\rho_z}^{\uparrow}\cdot\hat{\mathbf{p}}_{\rho_z}^{(\omega^{\pm})\downarrow}\right)\left(\hat{\mathbf{p}}_{z}^{\uparrow}\otimes\hat{\mathbf{p}}_{\rho_z''}^{(\omega^{\pm})\uparrow}\right)\right]^{*}e^{-i\left[k_{z,1}^{*}z-k_{z,2}^{*}\rho_z\right]}e^{ik_{z,2}^{\pm*}[\rho_z+\rho_z'']}\\ &\quad+\frac{i}{2}\frac{\delta[\rho_z-\rho_z'']}{k_{z,2}^{*}k_0^2(1\pm\tilde{\Omega})^2\varepsilon^{*}(\omega^{\pm})}t^{(\mathrm{p})*}\left(\left[\hat{\mathbf{p}}_{\rho_z}^{\uparrow}\right]^{*}\cdot\mathbf{z}\right)\left(\left[\hat{\mathbf{p}}_{z}^{\uparrow}\right]^{*}\otimes\hat{\mathbf{z}}\right)e^{-i\left[k_{z,1}^{*}z-k_{z,2}^{*}\rho_z\right]}\\ &= \left[\hat{\bar{\mathbf{T}}}_{z\leftarrow\rho_z\leftarrow\rho_z''}^{(\omega^{\pm})[(\updownarrow/\updownarrow)]}\right]^{*}+\left[\hat{\bar{\mathbf{R}}}_{z\leftarrow\rho_z\leftarrow\rho_z''}^{(\omega^{\pm})[(\downarrow/\uparrow)]}\right]^{*}+\left[\hat{\bar{\mathbf{Z}}}_{z\leftarrow\rho_z\leftarrow\rho_z''}^{(\omega^{\pm})}\right]^{*},\end{aligned}$$

[§] Notice that the frequency-shifted longitudinal wavevector can be expressed as $k_{z,i}^{\pm}=k_0(1\pm\tilde{\Omega})\sqrt{\varepsilon_i(\omega^{\pm})}\kappa_{z,i}^{\pm}$, where, according to Eq. (S.VI.2), it can be recast as $k_{z,i}^{\pm}=k_0\tilde{k}_{z,i}^{\pm}$ with $\tilde{k}_{z,i}^{\pm}=(1\pm\tilde{\Omega})\sqrt{\varepsilon_i(\omega^{\pm})}\kappa_{z,i}^{\pm}$, and $\kappa_{z,i}^{\pm}=\sqrt{1-\kappa_R^2/[(1\pm\tilde{\Omega})^2\varepsilon_i(\omega^{\pm})]}$.

$$\begin{aligned}\hat{\bar{\mathbf{G}}}^{(\omega^\pm)[(\updownarrow/\updownarrow)]}_{z\leftarrow\rho'_z\leftarrow\rho''_z} &= -\frac{1}{4}\frac{1}{k_{z,2}k^\pm_{z,2}}\left[t^{(\mathrm{s})}\hat{\mathbf{g}}^{(\mathrm{s})} + t^{(\mathrm{p})}\left(\hat{\mathbf{p}}^\uparrow_{\rho'_z}\cdot\hat{\mathbf{p}}^{(\omega^\pm)\updownarrow}_{\rho'_z}\right)\left(\hat{\mathbf{p}}^\uparrow_z\otimes\hat{\mathbf{p}}^{(\omega^\pm)\updownarrow}_{\rho''_z}\right)\right]e^{i\left[k_{z,1}z-k_{z,2}\rho'_z\right]}e^{ik^\pm_{z,2}\left|\rho'_z-\rho''_z\right|}\\
&\quad -\frac{1}{4}\frac{1}{k_{z,2}k^\pm_{z,2}}\left[t^{(\mathrm{s})}r^{(\mathrm{s})(\omega^\pm)}\hat{\mathbf{g}}^{(\mathrm{s})} + t^{(\mathrm{p})}r^{(\mathrm{p})(\omega^\pm)}\left(\hat{\mathbf{p}}^\uparrow_{\rho'_z}\cdot\hat{\mathbf{p}}^{(\omega^\pm)\downarrow}_{\rho'_z}\right)\left(\hat{\mathbf{p}}^\uparrow_z\otimes\hat{\mathbf{p}}^{(\omega^\pm)\uparrow}_{\rho''_z}\right)\right]e^{i\left[k_{z,1}z-k_{z,2}\rho'_z\right]}e^{-ik^\pm_{z,2}\left[\rho'_z+\rho''_z\right]}\\
&\quad -\frac{i}{2}\frac{\delta[\rho'_z-\rho''_z]}{k_{z,2}k_0^2(1\pm\tilde{\Omega})^2\varepsilon(\omega^\pm)}t^{(\mathrm{p})}\left(\hat{\mathbf{p}}^\uparrow_{\rho'_z}\cdot\hat{\mathbf{z}}\right)\left(\hat{\mathbf{p}}^\uparrow_z\otimes\hat{\mathbf{z}}\right)e^{i\left[k_{z,1}z-k_{z,2}\rho'_z\right]}\\
&= \hat{\bar{\mathbf{T}}}^{(\omega^\pm)[(\updownarrow/\updownarrow)]}_{z\leftarrow\rho'_z\leftarrow\rho''_z} + \hat{\bar{\mathbf{R}}}^{(\omega^\pm)[(\downarrow/\uparrow)]}_{z\leftarrow\rho'_z\leftarrow\rho''_z} + \hat{\bar{\mathbf{Z}}}^{(\omega^\pm)}_{z\leftarrow\rho'_z\leftarrow\rho''_z}.\end{aligned}$$

Then, according to this latter expressions, Eq. (S.VI.34) can be recast as:

$$\bar{\mathcal{G}}^{(\omega^\pm)}_{1,1} = \bar{\mathcal{G}}^{(\omega^\pm)}_{\mathrm{T-T}\,(1,1)} + \bar{\mathcal{G}}^{(\omega^\pm)}_{\mathrm{R-R}\,(1,1)} + 2\mathrm{Re}\left[\bar{\mathcal{G}}^{(\omega^\pm)}_{\mathrm{T-R}\,(1,1)}\right] + \bar{\mathcal{G}}^{(\omega^\pm)}_{\mathrm{Z-Z}\,(1,1)} + 2\mathrm{Re}\left[\bar{\mathcal{G}}^{(\omega^\pm)}_{\mathrm{T-Z}\,(1,1)}\right] + 2\mathrm{Re}\left[\bar{\mathcal{G}}^{(\omega^\pm)}_{\mathrm{R-Z}\,(1,1)}\right]. \quad \text{(S.VI.36)}$$

In the following we will proceed with the calculation of Eq. (S.VI.36) by analyzing separately each of the above contributions:

$$\left\|\left[\hat{\bar{\mathbf{T}}}^{(\omega^\pm)(\updownarrow/\updownarrow)}_{z\leftarrow\rho_z\leftarrow\rho''_z}\right]^*\hat{\bar{\mathbf{T}}}^{(\omega^\pm)(\updownarrow/\updownarrow)}_{z\leftarrow\rho'_z\leftarrow\rho''_z}\right\|_{\mathcal{F}} = \frac{1}{16}\frac{1}{\left|k_{z,2}\right|^2\left|k^\pm_{z,2}\right|^2}\sum_{i=\mathrm{s,p}}\left\|\left[\hat{\bar{\mathbf{t}}}^{(\omega^\pm)(\updownarrow/\updownarrow)}_{z\leftarrow\rho_z\leftarrow\rho''_z}\right]^*\hat{\bar{\mathbf{t}}}^{(\omega^\pm)(\updownarrow/\updownarrow)}_{z\leftarrow\rho'_z\leftarrow\rho''_z}\right\|^{(\mathrm{i})}_{\mathcal{F}}\bar{\Gamma}^{(\omega^\pm)(\updownarrow/\updownarrow)}_{\mathrm{T-T}\,(1,1)}, \quad \text{(S.VI.37)}$$

with (cf. Appendix VI.A for the demonstration of the p-like contribution),

$$\left\|\left[\hat{\bar{\mathbf{t}}}^{(\omega^\pm)(\updownarrow/\updownarrow)}_{z\leftarrow\rho_z\leftarrow\rho''_z}\right]^*\hat{\bar{\mathbf{t}}}^{(\omega^\pm)(\updownarrow/\updownarrow)}_{z\leftarrow\rho'_z\leftarrow\rho''_z}\right\|^{(\mathrm{s})}_{\mathcal{F}} = \left|t^{(\mathrm{s})}\right|^2\left\|\hat{\mathbf{g}}^{(\mathrm{s})}\right\|^2_{\mathcal{F}},$$

$$\left\|\left[\hat{\bar{\mathbf{t}}}^{(\omega^\pm)(\updownarrow/\updownarrow)}_{z\leftarrow\rho_z\leftarrow\rho''_z}\right]^*\hat{\bar{\mathbf{t}}}^{(\omega^\pm)(\updownarrow/\updownarrow)}_{z\leftarrow\rho'_z\leftarrow\rho''_z}\right\|^{(\mathrm{p})}_{\mathcal{F}} = \left|t^{(\mathrm{p})}\right|^2\left(\hat{\mathbf{p}}^\uparrow_{\rho_z}\cdot\hat{\mathbf{p}}^{(\omega^\pm)\updownarrow}_{\rho_z}\right)^*\left(\hat{\mathbf{p}}^\uparrow_{\rho'_z}\cdot\hat{\mathbf{p}}^{(\omega^\pm)\updownarrow}_{\rho'_z}\right)\left(\left[\hat{\mathbf{p}}^\uparrow_z\right]^*\cdot\hat{\mathbf{p}}^\uparrow_z\right)\left(\left[\hat{\mathbf{p}}^{(\omega^\pm)\updownarrow}_{\rho''_z}\right]^*\cdot\hat{\mathbf{p}}^{(\omega^\pm)\updownarrow}_{\rho''_z}\right).$$

Likewise, regarding the spatial integrals,

$$\begin{aligned}\bar{\Gamma}^{(\omega^\pm)(\updownarrow/\updownarrow)}_{\mathrm{T-T}\,(1,1)}(z,\rho_z,\rho'_z,\rho''_z) &= e^{-i\left[k^*_{z,1}z-k^*_{z,2}\rho_z\right]}e^{-ik^{\pm*}_{z,2}\left|\rho_z-\rho''_z\right|}e^{i\left[k_{z,1}z-k_{z,2}\rho'_z\right]}e^{ik^\pm_{z,2}\left|\rho'_z-\rho''_z\right|}\\
&= e^{-2\mathrm{Im}[k_{z,1}]z}e^{ik^*_{z,2}\rho_z}e^{-ik_{z,2}\rho'_z}e^{-ik^{\pm*}_{z,2}\left|\rho_z-\rho''_z\right|}e^{ik^\pm_{z,2}\left|\rho'_z-\rho''_z\right|}. \quad \text{(S.VI.38)}\end{aligned}$$

According to Eq. (S.VI.34), this latter expression has to be integrated over ρ_z, ρ'_z, and ρ''_z. To do so, it should be noted the presence of the absolute value in some of the exponents so as to distinguish between the four possible cases:

- **Case** $(\uparrow/\uparrow)$: $\rho_z > \rho''_z$ and $\rho'_z > \rho''_z \iff (\rho_z,\rho'_z) > \rho''_z$;
- **Case** $(\uparrow/\downarrow)$: $\rho_z > \rho''_z$ and $\rho'_z < \rho''_z \iff \rho_z > \rho''_z > \rho'_z$;
- **Case** $(\downarrow/\uparrow)$: $\rho_z < \rho''_z$ and $\rho'_z > \rho''_z \iff \rho'_z > \rho''_z > \rho_z$;
- **Case** $(\downarrow/\downarrow)$: $\rho_z < \rho''_z$ and $\rho'_z < \rho''_z \iff (\rho_z,\rho'_z) < \rho''_z$.

Therefore, the spatial integral in Eq. (S.VI.34) can be recast as:

$$e^{-2\mathrm{Im}[k_{z,1}]z}\int_{-\infty}^{0}d\rho''_z\left[\underbrace{\iint_{\rho''_z}^{0}d\rho'_z d\rho_z}_{\text{Case }(\uparrow/\uparrow)} + \underbrace{\int_{-\infty}^{\rho''_z}d\rho'_z\int_{\rho''_z}^{0}d\rho_z}_{\text{Case }(\uparrow/\downarrow)} + \underbrace{\int_{\rho''_z}^{0}d\rho'_z\int_{-\infty}^{\rho''_z}d\rho_z}_{\text{Case }(\downarrow/\uparrow)} + \underbrace{\iint_{-\infty}^{\rho''_z}d\rho'_z d\rho_z}_{\text{Case }(\downarrow/\downarrow)}\right], \quad \text{(S.VI.39)}$$

thereby covering the whole time-modulated medium. Obviously, all of these integrals are to be put in correspondence with the dyadic Green's functions given above. Thus, by putting it all together, the first term of Eq. (S.VI.36) can be expressed as:

$$\bar{\mathcal{G}}^{(\omega^{\pm})}_{\mathrm{T-T}\,(1,1)} = \frac{1}{32\pi k_0^5} \int_0^{+\infty} d\kappa_R \frac{\kappa_R}{|\tilde{k}_{z,2}|^2 |\tilde{k}^{\pm}_{z,2}|^2} \sum_{i,j=\{\uparrow,\downarrow\}} \bar{\mathcal{G}}^{(\omega^{\pm})(i/j)}_{\mathrm{T-T}\,(1,1)} \bar{\Gamma}^{(\omega^{\pm})(i/j)}_{\mathrm{T-T}\,(1,1)}, \tag{S.VI.40}$$

where

$$\bar{\mathcal{G}}^{(\omega^{\pm})(\uparrow/\uparrow)}_{\mathrm{T-T}\,(1,1)} = \left|t^{(\mathrm{s})}\right|^2 + \left|t^{(\mathrm{p})}\right|^2 \frac{\mathrm{T}^{(\omega^{\pm})}}{(1 \pm \tilde{\Omega})^2 \, |\varepsilon(\omega)| \, |\varepsilon(\omega^{\pm})|} \left\{ \left[\tilde{k}^*_{z,2}\tilde{k}^{\pm *}_{z,2} + \kappa_R^2\right] \left[\tilde{k}_{z,2}\tilde{k}^{\pm}_{z,2} + \kappa_R^2\right] \right\},$$

$$\bar{\Gamma}^{(\omega^{\pm})(\uparrow/\uparrow)}_{\mathrm{T-T}\,(1,1)} = \frac{2e^{-2k_0 \mathrm{Im}[\tilde{k}_{z,1}]z}}{|\tilde{k}_{z,2} - \tilde{k}^{\pm}_{z,2}|^2} \left[\frac{\mathrm{Im}[\tilde{k}_{z,2}] + \mathrm{Im}[\tilde{k}^{\pm}_{z,2}]}{4\mathrm{Im}[\tilde{k}_{z,2}]\mathrm{Im}[\tilde{k}^{\pm}_{z,2}]} - \frac{\mathrm{Im}[\tilde{k}_{z,2}] + \mathrm{Im}[\tilde{k}^{\pm}_{z,2}]}{|\tilde{k}_{z,2} - \tilde{k}^{\pm *}_{z,2}|^2} \right],$$

$$\bar{\mathcal{G}}^{(\omega^{\pm})(\uparrow/\downarrow)}_{\mathrm{T-T}\,(1,1)} = \left|t^{(\mathrm{s})}\right|^2 + \left|t^{(\mathrm{p})}\right|^2 \frac{\mathrm{R}^{(\omega^{\pm})}}{(1 \pm \tilde{\Omega})^2 \, |\varepsilon(\omega)| \, |\varepsilon(\omega^{\pm})|} \left\{ \left[\tilde{k}^*_{z,2}\tilde{k}^{\pm *}_{z,2} + \kappa_R^2\right] \left[\tilde{k}_{z,2}\tilde{k}^{\pm}_{z,2} - \kappa_R^2\right] \right\},$$

$$\bar{\Gamma}^{(\omega^{\pm})(\uparrow/\downarrow)}_{\mathrm{T-T}\,(1,1)} = \frac{e^{-2k_0 \mathrm{Im}[\tilde{k}_{z,1}]z}}{2\mathrm{Im}[\tilde{k}^*_{z,2}][\tilde{k}_{z,2} - \tilde{k}^{\pm *}_{z,2}][\tilde{k}_{z,2} + \tilde{k}^{\pm}_{z,2}]},$$

$$\bar{\mathcal{G}}^{(\omega^{\pm})(\downarrow/\uparrow)}_{\mathrm{T-T}\,(1,1)} = \left|t^{(\mathrm{s})}\right|^2 + \left|t^{(\mathrm{p})}\right|^2 \frac{\mathrm{R}^{(\omega^{\pm})}}{(1 \pm \tilde{\Omega})^2 \, |\varepsilon(\omega)| \, |\varepsilon(\omega^{\pm})|} \left\{ \left[\tilde{k}^*_{z,2}\tilde{k}^{\pm *}_{z,2} - \kappa_R^2\right] \left[\tilde{k}_{z,2}\tilde{k}^{\pm}_{z,2} + \kappa_R^2\right] \right\},$$

$$\bar{\Gamma}^{(\omega^{\pm})(\downarrow/\uparrow)}_{\mathrm{T-T}\,(1,1)} = \frac{e^{-2k_0 \mathrm{Im}[\tilde{k}_{z,1}]z}}{2\mathrm{Im}[\tilde{k}^*_{z,2}][\tilde{k}^*_{z,2} - \tilde{k}^{\pm}_{z,2}][\tilde{k}^*_{z,2} + \tilde{k}^{\pm *}_{z,2}]},$$

$$\bar{\mathcal{G}}^{(\omega^{\pm})(\downarrow/\downarrow)}_{\mathrm{T-T}\,(1,1)} = \left|t^{(\mathrm{s})}\right|^2 + \left|t^{(\mathrm{p})}\right|^2 \frac{\mathrm{T}^{(\omega^{\pm})}}{(1 \pm \tilde{\Omega})^2 \, |\varepsilon(\omega)| \, |\varepsilon(\omega^{\pm})|} \left\{ \left[\tilde{k}^*_{z,2}\tilde{k}^{\pm *}_{z,2} - \kappa_R^2\right] \left[\tilde{k}_{z,2}\tilde{k}^{\pm}_{z,2} - \kappa_R^2\right] \right\},$$

$$\bar{\Gamma}^{(\omega^{\pm})(\downarrow/\downarrow)}_{\mathrm{T-T}\,(1,1)} = \frac{e^{-2\mathrm{Im}[\tilde{k}_{z,1}]z}}{2\mathrm{Im}[\tilde{k}_{z,2}]|\tilde{k}_{z,2} + \tilde{k}^{\pm}_{z,2}|^2},$$

with

$$\mathrm{T}^{(\omega^{\pm})} = \frac{1}{(1 \pm \tilde{\Omega})^2 \, |\varepsilon(\omega^{\pm})|} \left\{ |\tilde{k}_{z,1}|^2 |\tilde{k}^{\pm}_{z,2}|^2 + \kappa_R^2 \left[|\tilde{k}^{\pm}_{z,2}|^2 + |\tilde{k}_{z,1}|^2 + \kappa_R^2 \right] \right\}, \tag{S.VI.41a}$$

$$\mathrm{R}^{(\omega^{\pm})} = \frac{1}{(1 \pm \tilde{\Omega})^2 \, |\varepsilon(\omega^{\pm})|} \left\{ |\tilde{k}_{z,1}|^2 |\tilde{k}^{\pm}_{z,2}|^2 + \kappa_R^2 \left[|\tilde{k}^{\pm}_{z,2}|^2 - |\tilde{k}_{z,1}|^2 - \kappa_R^2 \right] \right\}. \tag{S.VI.41b}$$

Proceeding similarly with the rest of contributions of Eq. (S.VI.36):

$$\bar{\mathcal{G}}^{(\omega^{\pm})}_{\mathrm{R-R}\,(1,1)} = \frac{1}{32\pi k_0^5} \int_0^{+\infty} d\kappa_R \frac{\kappa_R}{|\tilde{k}_{z,2}|^2 |\tilde{k}^{\pm}_{z,2}|^2} \bar{\mathcal{G}}^{(\omega^{\pm})(\downarrow/\downarrow)}_{\mathrm{R-R}\,(1,1)} \bar{\Gamma}^{(\omega^{\pm})}_{\mathrm{R-R}\,(1,1)}, \tag{S.VI.42}$$

where

$$\bar{\mathcal{G}}^{(\omega^{\pm})(\downarrow/\downarrow)}_{\mathrm{R-R}\,(1,1)} = \left|t^{(\mathrm{s})}\right|^2 \left|r^{(\mathrm{s})(\omega^{\pm})}\right|^2 + \left|t^{(\mathrm{p})}\right|^2 \left|r^{(\mathrm{p})(\omega^{\pm})}\right|^2 \frac{\mathrm{T}^{(\omega^{\pm})}}{(1 \pm \tilde{\Omega})^2 \, |\varepsilon(\omega)| \, |\varepsilon(\omega^{\pm})|} \left\{ \left[\tilde{k}^*_{z,2}\tilde{k}^{\pm *}_{z,2} - \kappa_R^2\right] \left[\tilde{k}_{z,2}\tilde{k}^{\pm}_{z,2} - \kappa_R^2\right] \right\},$$

$$\bar{\Gamma}^{(\omega^{\pm})}_{\mathrm{R-R}\,(1,1)} = \frac{e^{-2k_0 \mathrm{Im}[\tilde{k}_{z,1}]z}}{2\mathrm{Im}[\tilde{k}^{\pm}_{z,2}]|\tilde{k}_{z,2} + \tilde{k}^{\pm}_{z,2}|^2},$$

$$\bar{\mathcal{G}}^{(\omega^\pm)}_{\mathrm{T-R}\,(1,1)} = \frac{1}{32\pi k_0^5}\int_0^{+\infty} d\kappa_R \frac{\kappa_R}{|\tilde{k}_{z,2}|^2|\tilde{k}^{\pm}_{z,2}|^2}\sum_{i=\{\uparrow,\downarrow\}}\bar{\mathcal{G}}^{(\omega^\pm)(i/\downarrow)}_{\mathrm{T-R}\,(1,1)}\bar{\Gamma}^{(\omega^\pm)(i)}_{\mathrm{T-R}\,(1,1)}, \tag{S.VI.43}$$

where

$$\bar{\mathcal{G}}^{(\omega^\pm)(\uparrow/\downarrow)}_{\mathrm{T-R}\,(1,1)} = \left|t^{(\mathrm{s})}\right|^2 r^{(\mathrm{s})(\omega^\pm)} + \left|t^{(\mathrm{p})}\right|^2 r^{(\mathrm{p})(\omega^\pm)}\frac{\mathrm{T}^{(\omega^\pm)}}{(1\pm\tilde{\Omega})^2\,|\varepsilon(\omega)|\,|\varepsilon(\omega^\pm)|}\left\{\left[\tilde{k}^*_{z,2}\tilde{k}^{\pm *}_{z,2}+\kappa_R^2\right]\left[\tilde{k}_{z,2}\tilde{k}^{\pm}_{z,2}-\kappa_R^2\right]\right\},$$

$$\bar{\Gamma}^{(\omega^\pm)(\uparrow)}_{\mathrm{T-R}\,(1,1)} = \frac{e^{-2k_0\mathrm{Im}[\tilde{k}_{z,1}]z}}{2\mathrm{Im}[\tilde{k}^{\pm}_{z,2}][\tilde{k}_{z,2}+\tilde{k}^{\pm}_{z,2}][\tilde{k}^*_{z,2}-\tilde{k}^{\pm}_{z,2}]},$$

$$\bar{\mathcal{G}}^{(\omega^\pm)(\downarrow/\downarrow)}_{\mathrm{T-R}\,(1,1)} = \left|t^{(\mathrm{s})}\right|^2 r^{(\mathrm{s})(\omega^\pm)} + \left|t^{(\mathrm{p})}\right|^2 r^{(\mathrm{p})(\omega^\pm)}\frac{\mathrm{R}^{(\omega^\pm)}}{(1\pm\tilde{\Omega})^2\,|\varepsilon(\omega)|\,|\varepsilon(\omega^\pm)|}\left\{\left[\tilde{k}^*_{z,2}\tilde{k}^{\pm *}_{z,2}-\kappa_R^2\right]\left[\tilde{k}_{z,2}\tilde{k}^{\pm}_{z,2}-\kappa_R^2\right]\right\},$$

$$\bar{\Gamma}^{(\omega^\pm)(\downarrow)}_{\mathrm{T-R}\,(1,1)} = \frac{e^{-2k_0\mathrm{Im}[\tilde{k}_{z,1}]z}}{i|\tilde{k}_{z,2}+\tilde{k}^{\pm}_{z,2}|^2[\tilde{k}^*_{z,2}-\tilde{k}^{\pm}_{z,2}]},$$

$$\bar{\mathcal{G}}^{(\omega^\pm)}_{\mathrm{Z-Z}\,(1,1)} = \frac{1}{32\pi k_0^5}\int_0^{+\infty} d\kappa_R \frac{2\kappa_R^3(|\tilde{k}_{z,1}|^2+\kappa_R^2)}{|\tilde{k}_{z,2}|^2(1\pm\tilde{\Omega})^4\,|\varepsilon(\omega)|\,|\varepsilon(\omega^\pm)|^2}\left|t^{(\mathrm{p})}\right|^2\frac{e^{-2k_0\mathrm{Im}[\tilde{k}_{z,1}]z}}{\mathrm{Im}[\tilde{k}_{z,2}]}, \tag{S.VI.44}$$

$$\bar{\mathcal{G}}^{(\omega^\pm)}_{\mathrm{T-Z}\,(1,1)} = \frac{1}{32\pi k_0^5}\int_0^{+\infty} d\kappa_R \frac{2\kappa_R^3(|\tilde{k}_{z,1}|^2+\kappa_R^2)}{|\tilde{k}_{z,2}|^2(1\pm\tilde{\Omega})^4\,|\varepsilon(\omega)|\,|\varepsilon(\omega^\pm)|^2}\left|t^{(\mathrm{p})}\right|^2\sum_{i=\{\uparrow,\downarrow\}}\bar{\mathcal{G}}^{(\omega^\pm)(i)}_{\mathrm{T-Z}\,(1,1)}\bar{\Gamma}^{(\omega^\pm)(i)}_{\mathrm{T-Z}\,(1,1)}, \tag{S.VI.45}$$

where

$$\bar{\mathcal{G}}^{(\omega^\pm)(\uparrow)}_{\mathrm{T-Z}\,(1,1)} = \left[\tilde{k}^*_{z,2}\tilde{k}^{\pm *}_{z,2}+\kappa_R^2\right],$$

$$\bar{\Gamma}^{(\omega^\pm)(\uparrow)}_{\mathrm{T-Z}\,(1,1)} = \frac{e^{-2k_0\mathrm{Im}[\tilde{k}_{z,1}]z}}{2\mathrm{Im}[\tilde{k}^*_{z,2}][\tilde{k}_{z,2}-\tilde{k}^{\pm *}_{z,2}]\tilde{k}^{\pm *}_{z,2}},$$

$$\bar{\mathcal{G}}^{(\omega^\pm)(\downarrow)}_{\mathrm{T-Z}\,(1,1)} = \left[\tilde{k}^*_{z,2}\tilde{k}^{\pm *}_{z,2}-\kappa_R^2\right],$$

$$\bar{\Gamma}^{(\omega^\pm)(\downarrow)}_{\mathrm{T-Z}\,(1,1)} = \frac{e^{-2k_0\mathrm{Im}[\tilde{k}_{z,1}]z}}{2\mathrm{Im}[\tilde{k}^*_{z,2}][\tilde{k}^*_{z,2}+\tilde{k}^{\pm *}_{z,2}]\tilde{k}^{\pm *}_{z,2}};$$

$$\bar{\mathcal{G}}^{(\omega^\pm)}_{\mathrm{R-Z}\,(1,1)} = \frac{1}{32\pi k_0^5}\int_0^{+\infty} d\kappa_R \frac{2\kappa_R^3(|\tilde{k}_{z,1}|^2+\kappa_R^2)}{|\tilde{k}_{z,2}|^2(1\pm\tilde{\Omega})^4\,|\varepsilon(\omega)|\,|\varepsilon(\omega^\pm)|^2}\left|t^{(\mathrm{p})}\right|^2\bar{\mathcal{G}}^{(\omega^\pm)(\downarrow)}_{\mathrm{R-Z}\,(1,1)}\bar{\Gamma}^{(\omega^\pm)}_{\mathrm{R-Z}\,(1,1)}, \tag{S.VI.46}$$

where

$$\bar{\mathcal{G}}^{(\omega^\pm)(\downarrow)}_{\mathrm{R-Z}\,(1,1)} = r^{(\mathrm{p})(\omega^\pm)*}\left[\tilde{k}^*_{z,2}\tilde{k}^{\pm *}_{z,2}-\kappa_R^2\right],$$

$$\bar{\Gamma}^{(\omega^\pm)}_{\mathrm{R-Z}\,(1,1)} = \frac{e^{-2k_0\mathrm{Im}[\tilde{k}_{z,1}]z}}{-i[\tilde{k}_{z,2}-\tilde{k}^{\pm *}_{z,2}][\tilde{k}^*_{z,2}+\tilde{k}^{\pm *}_{z,2}]\tilde{k}^{\pm *}_{z,2}}.$$

Calculation of $\tilde{\mathcal{G}}_{1,1}^{(\omega^\pm)}$:

Now, following a similar procedure, we address the calculation of $\tilde{\mathcal{G}}_{1,1}^{(\omega^\pm)}$. In this case, the order of the matrices involved is to be:

$$[\hat{E}_{1,\alpha}^{(+)}]^\dagger \cdot \hat{E}_{1,\alpha}^{(+)} \propto G^*_{\alpha,\gamma}\hat{j}^\dagger_{1,\gamma}G_{\alpha,\delta}\hat{j}_{1,\delta} \propto G^*_{\alpha,\gamma}G_{\gamma,\beta}\hat{j}_{0,\beta}G_{\alpha,\delta}G^*_{\delta,\zeta}\hat{j}^\dagger_{0,\zeta} \propto G^*_{\alpha,\gamma}G_{\gamma,\beta}G_{\alpha,\delta}G^*_{\delta,\zeta}\delta_{\beta,\zeta} \propto G^*_{\alpha,\gamma}G_{\gamma,\beta}G_{\alpha,\delta}G^*_{\delta,\beta}.$$

In that way, it is easy to see that the four matrices can again be grouped in two pairs of customary products:

$$\begin{aligned}\mathbf{G}^*(\mathbf{r},\boldsymbol{\rho},\omega)\mathbf{G}(\boldsymbol{\rho},\boldsymbol{\rho}'',\omega^\pm)\mathbf{G}(\mathbf{r},\boldsymbol{\rho}',\omega)\mathbf{G}^*(\boldsymbol{\rho}',\boldsymbol{\rho}'',\omega^\pm) &= G^*_{\alpha,\gamma}(\mathbf{r},\boldsymbol{\rho},\omega)G_{\gamma,\beta}(\boldsymbol{\rho},\boldsymbol{\rho}'',\omega^\pm)G_{\alpha,\delta}(\mathbf{r},\boldsymbol{\rho}',\omega)G^*_{\delta,\beta}(\boldsymbol{\rho}',\boldsymbol{\rho}'',\omega^\pm)\\ &= \left|\tilde{G}_{\alpha,\beta}(\mathbf{r},\boldsymbol{\rho},\boldsymbol{\rho}'',\omega,\omega^\pm)\right|^2\left|\tilde{G}_{\alpha,\beta}(\mathbf{r},\boldsymbol{\rho}',\boldsymbol{\rho}'',\omega,\omega^\pm)\right|^2,\end{aligned}$$

with $|\tilde{G}_{\alpha,\beta}(\mathbf{r},\boldsymbol{\rho},\boldsymbol{\rho}'',\omega,\omega^\pm)|^2 \equiv G^*_{\alpha,\gamma}(\mathbf{r},\boldsymbol{\rho},\omega)G_{\gamma,\beta}(\boldsymbol{\rho},\boldsymbol{\rho}'',\omega^\pm)$, $|\tilde{G}_{\alpha,\beta}(\mathbf{r},\boldsymbol{\rho}',\boldsymbol{\rho}'',\omega,\omega^\pm)|^2 \equiv G_{\alpha,\delta}(\mathbf{r},\boldsymbol{\rho}',\omega)G^*_{\delta,\beta}(\boldsymbol{\rho}',\boldsymbol{\rho}'',\omega^\pm)$, and noticing, once again, that the summation over γ and δ is actually the customary matrix product. Inserting this latter expression into (S.VI.33b), and applying the expansion given in Eq. (S.VI.1), it follows that:

$$\begin{aligned}\tilde{\mathcal{G}}_{1,1}^{(\omega^\pm)} &= \iiint_{\mathcal{V}} d^3\boldsymbol{\rho}d^3\boldsymbol{\rho}'d^3\boldsymbol{\rho}''\mathrm{Tr}[\mathbf{G}^*(\mathbf{r},\boldsymbol{\rho},\omega)\mathbf{G}(\boldsymbol{\rho},\boldsymbol{\rho}'',\omega^\pm)\mathbf{G}(\mathbf{r},\boldsymbol{\rho}',\omega)\mathbf{G}^*(\boldsymbol{\rho}',\boldsymbol{\rho}'',\omega^\pm)]\\ &= \frac{k_0^2}{4\pi^2}\iiint_{-\infty}^{0} d\rho_z d\rho'_z d\rho''_z \iint_{-\infty}^{+\infty} d^2\boldsymbol{\kappa}_\parallel \mathrm{Tr}[\hat{\mathbf{G}}^*(\kappa_x,\kappa_y;\omega|z,\rho_z)\hat{\mathbf{G}}(\kappa_x^\pm,\kappa_y^\pm;\omega^\pm|\rho_z,\rho''_z)\hat{\mathbf{G}}(\kappa_x,\kappa_y;\omega|z,\rho'_z)\hat{\mathbf{G}}^*(\kappa_x^\pm,\kappa_y^\pm;\omega^\pm|\rho'_z,\rho''_z)].\end{aligned}$$

Hence:

$$\tilde{\mathcal{G}}_{1,1}^{(\omega^\pm)} = \frac{k_0^2}{4\pi^2}\iiint_{-\infty}^{0} d\rho_z d\rho'_z d\rho''_z \int_0^{+\infty} d\kappa_R\kappa_R \int_0^{2\pi} d\kappa_\varphi \left\|\left|\hat{\tilde{\mathbf{G}}}^{(\omega^\pm)[(\uparrow/\uparrow)(\updownarrow/\updownarrow)]}_{z\leftarrow\rho_z\leftarrow\rho''_z}\right|\left|\hat{\tilde{\mathbf{G}}}^{(\omega^\pm)[(\uparrow/\uparrow)(\updownarrow/\updownarrow)]}_{z\leftarrow\rho'_z\leftarrow\rho''_z}\right|\right\|_{\mathcal{F}}, \quad \text{(S.VI.47)}$$

where

$$\left|\hat{\tilde{\mathbf{G}}}^{(\omega^\pm)[(\uparrow/\uparrow)(\updownarrow/\updownarrow)]}_{z\leftarrow\rho_z\leftarrow\rho''_z}\right| \equiv \left|\hat{\tilde{\mathbf{G}}}^{(\omega^\pm)[(\updownarrow/\updownarrow)]}_{z\leftarrow\rho_z\leftarrow\rho''_z}\right| = \left[\hat{\mathbf{G}}^{(\uparrow/\uparrow)}(\kappa_x,\kappa_y;\omega|z,\rho_z)\right]^*\cdot\left[\hat{\mathbf{G}}^{(\updownarrow/\updownarrow)}(\kappa_x^\pm,\kappa_y^\pm;\omega^\pm|\rho_z,\rho''_z)\right], \quad \text{(S.VI.48a)}$$

$$\left|\hat{\tilde{\mathbf{G}}}^{(\omega^\pm)[(\uparrow/\uparrow)(\updownarrow/\updownarrow)]}_{z\leftarrow\rho'_z\leftarrow\rho''_z}\right| \equiv \left|\hat{\tilde{\mathbf{G}}}^{(\omega^\pm)[(\updownarrow/\updownarrow)]}_{z\leftarrow\rho'_z\leftarrow\rho''_z}\right| = \hat{\mathbf{G}}^{(\uparrow/\uparrow)}(\kappa_x,\kappa_y;\omega|z,\rho'_z)\cdot\left[\hat{\mathbf{G}}^{(\updownarrow/\updownarrow)}(\kappa_x^\pm,\kappa_y^\pm;\omega^\pm|\rho'_z,\rho''_z)\right]^*. \quad \text{(S.VI.48b)}$$

Then, proceeding as previously,

$$\begin{aligned}\left|\hat{\tilde{\mathbf{G}}}^{(\omega^\pm)[(\updownarrow/\updownarrow)]}_{z\leftarrow\rho_z\leftarrow\rho''_z}\right| &= \frac{1}{4}\frac{1}{k^*_{z,2}k^\pm_{z,2}}\left[t^{(\mathrm{s})*}\hat{\mathbf{g}}^{(\mathrm{s})} + t^{(\mathrm{p})*}\left(\left[\hat{\mathbf{p}}^\uparrow_{\rho_z}\right]^*\cdot\hat{\mathbf{p}}^{(\omega^\pm)\updownarrow}_{\rho_z}\right)\left(\left[\hat{\mathbf{p}}^\uparrow_z\right]^*\otimes\hat{\mathbf{p}}^{(\omega^\pm)\updownarrow}_{\rho''_z}\right)\right]e^{-i\left[k^*_{z,1}z-k^*_{z,2}\rho_z\right]}e^{ik^\pm_{z,2}|\rho_z-\rho''_z|}\\ &+\frac{1}{4}\frac{1}{k^*_{z,2}k^\pm_{z,2}}\left[t^{(\mathrm{s})*}r^{(\mathrm{s})(\omega^\pm)}\hat{\mathbf{g}}^{(\mathrm{s})} + t^{(\mathrm{p})*}r^{(\mathrm{p})(\omega^\pm)}\left(\left[\hat{\mathbf{p}}^\uparrow_{\rho_z}\right]^*\cdot\hat{\mathbf{p}}^{(\omega^\pm)\downarrow}_{\rho_z}\right)\left(\left[\hat{\mathbf{p}}^\uparrow_z\right]^*\otimes\hat{\mathbf{p}}^{(\omega^\pm)\uparrow}_{\rho''_z}\right)\right]e^{-i\left[k^*_{z,1}z-k^*_{z,2}\rho_z\right]}e^{-ik^\pm_{z,2}\left[\rho_z+\rho''_z\right]}\\ &+\frac{i}{2}\frac{\delta[\rho_z-\rho''_z]}{k^*_{z,2}k_0^2(1\pm\tilde{\Omega})^2\varepsilon(\omega^\pm)}t^{(\mathrm{p})*}\left(\left[\hat{\mathbf{p}}^\uparrow_{\rho_z}\right]^*\cdot\hat{\mathbf{z}}\right)\left(\left[\hat{\mathbf{p}}^\uparrow_z\right]^*\otimes\hat{\mathbf{z}}\right)e^{-i\left[k^*_{z,1}z-k^*_{z,2}\rho_z\right]}\\ &= \left|\hat{\tilde{\mathbf{T}}}^{(\omega^\pm)[(\updownarrow/\updownarrow)]}_{z\leftarrow\rho_z\leftarrow\rho''_z}\right| + \left|\hat{\tilde{\mathbf{R}}}^{(\omega^\pm)[(\downarrow/\uparrow)]}_{z\leftarrow\rho_z\leftarrow\rho''_z}\right| + \left|\hat{\tilde{\mathbf{Z}}}^{(\omega^\pm)}_{z\leftarrow\rho_z\leftarrow\rho''_z}\right|,\end{aligned}$$

$$\begin{aligned}\left|\hat{\tilde{\mathbf{G}}}^{(\omega^\pm)[(\updownarrow/\updownarrow)]}_{z\leftarrow\rho'_z\leftarrow\rho''_z}\right| &= \frac{1}{4}\frac{1}{k_{z,2}k^{\pm*}_{z,2}}\left[t^{(\mathrm{s})}\hat{\mathbf{g}}^{(\mathrm{s})} + t^{(\mathrm{p})}\left(\hat{\mathbf{p}}^\uparrow_{\rho'_z}\cdot\left[\hat{\mathbf{p}}^{(\omega^\pm)\updownarrow}_{\rho'_z}\right]^*\right)\left(\hat{\mathbf{p}}^\uparrow_z\otimes\left[\hat{\mathbf{p}}^{(\omega^\pm)\updownarrow}_{\rho''_z}\right]^*\right)\right]e^{i\left[k_{z,1}z-k_{z,2}\rho'_z\right]}e^{-ik^{\pm*}_{z,2}|\rho'_z-\rho''_z|}\\ &+\frac{1}{4}\frac{1}{k_{z,2}k^{\pm*}_{z,2}}\left[t^{(\mathrm{s})}r^{(\mathrm{s})(\omega^\pm)*}\hat{\mathbf{g}}^{(\mathrm{s})} + t^{(\mathrm{p})}r^{(\mathrm{p})(\omega^\pm)*}\left(\hat{\mathbf{p}}^\uparrow_{\rho'_z}\cdot\left[\hat{\mathbf{p}}^{(\omega^\pm)\downarrow}_{\rho'_z}\right]^*\right)\left(\hat{\mathbf{p}}^\uparrow_z\otimes\left[\hat{\mathbf{p}}^{(\omega^\pm)\uparrow}_{\rho''_z}\right]^*\right)\right]e^{i\left[k_{z,1}z-k_{z,2}\rho'_z\right]}e^{ik^{\pm*}_{z,2}\left[\rho'_z+\rho''_z\right]}\\ &-\frac{i}{2}\frac{\delta[\rho'_z-\rho''_z]}{k_{z,2}k_0^2(1\pm\tilde{\Omega})^2\varepsilon^*(\omega^\pm)}t^{(\mathrm{p})}\left(\hat{\mathbf{p}}^\uparrow_{\rho'_z}\cdot\hat{\mathbf{z}}\right)\left(\hat{\mathbf{p}}^\uparrow_z\otimes\hat{\mathbf{z}}\right)e^{i\left[k_{z,1}z-k_{z,2}\rho'_z\right]}\\ &= \left|\hat{\tilde{\mathbf{T}}}^{(\omega^\pm)[(\updownarrow/\updownarrow)]}_{z\leftarrow\rho'_z\leftarrow\rho''_z}\right| + \left|\hat{\tilde{\mathbf{R}}}^{(\omega^\pm)[(\downarrow/\uparrow)]}_{z\leftarrow\rho'_z\leftarrow\rho''_z}\right| + \left|\hat{\tilde{\mathbf{Z}}}^{(\omega^\pm)}_{z\leftarrow\rho'_z\leftarrow\rho''_z}\right|.\end{aligned}$$

Therefore, as in the previous case, Eq. (S.VI.47) can be recast as:

$$\tilde{\mathcal{G}}^{(\omega^\pm)}_{1,1} = \tilde{\mathcal{G}}^{(\omega^\pm)}_{\mathrm{T-T}\,(1,1)} + \tilde{\mathcal{G}}^{(\omega^\pm)}_{\mathrm{R-R}\,(1,1)} + 2\mathrm{Re}\Big[\tilde{\mathcal{G}}^{(\omega^\pm)}_{\mathrm{T-R}\,(1,1)}\Big] + \tilde{\mathcal{G}}^{(\omega^\pm)}_{\mathrm{Z-Z}\,(1,1)} + 2\mathrm{Re}\Big[\tilde{\mathcal{G}}^{(\omega^\pm)}_{\mathrm{T-Z}\,(1,1)}\Big] + 2\mathrm{Re}\Big[\tilde{\mathcal{G}}^{(\omega^\pm)}_{\mathrm{R-Z}\,(1,1)}\Big]. \quad \text{(S.VI.49)}$$

From this, we proceed with the calculation of Eq. (S.VI.49) by analyzing separately each of the above contributions (cf. Appendix VI.B for the demonstration of the p-like contribution to the (T-T)-term):

$$\tilde{\mathcal{G}}^{(\omega^\pm)}_{\mathrm{T-T}\,(1,1)} = \frac{1}{32\pi k_0^5}\int_0^{+\infty} d\kappa_R \frac{\kappa_R}{|\tilde{k}_{z,2}|^2|\tilde{k}^\pm_{z,2}|^2}\sum_{i,j=\{\uparrow,\downarrow\}} \tilde{\mathcal{G}}^{(\omega^\pm)(i/j)}_{\mathrm{T-T}\,(1,1)}\tilde{\Gamma}^{(\omega^\pm)(i/j)}_{\mathrm{T-T}\,(1,1)}, \quad \text{(S.VI.50)}$$

where

$$\tilde{\mathcal{G}}^{(\omega^\pm)(\uparrow/\uparrow)}_{\mathrm{T-T}\,(1,1)} = \left|t^{(\mathrm{s})}\right|^2 + \left|t^{(\mathrm{p})}\right|^2 \frac{\mathrm{T}^{(\omega^\pm)}}{(1\pm\tilde{\Omega})^2\,|\varepsilon(\omega)|\,|\varepsilon(\omega^\pm)|}\left\{\left[\tilde{k}^*_{z,2}\tilde{k}^\pm_{z,2} + \kappa_R^2\right]\left[\tilde{k}_{z,2}\tilde{k}^{\pm*}_{z,2} + \kappa_R^2\right]\right\},$$

$$\tilde{\Gamma}^{(\omega^\pm)(\uparrow/\uparrow)}_{\mathrm{T-T}\,(1,1)} = \frac{2e^{-2k_0\mathrm{Im}[\tilde{k}_{z,1}]z}}{|\tilde{k}_{z,2}+\tilde{k}^{\pm*}_{z,2}|^2}\left[\frac{\mathrm{Im}[\tilde{k}_{z,2}]+\mathrm{Im}[\tilde{k}^\pm_{z,2}]}{4\mathrm{Im}[\tilde{k}_{z,2}]\mathrm{Im}[\tilde{k}^\pm_{z,2}]} - \frac{\mathrm{Im}[\tilde{k}_{z,2}]+\mathrm{Im}[\tilde{k}^\pm_{z,2}]}{|\tilde{k}_{z,2}+\tilde{k}^\pm_{z,2}|^2}\right],$$

$$\tilde{\mathcal{G}}^{(\omega^\pm)(\uparrow/\downarrow)}_{\mathrm{T-T}\,(1,1)} = \left|t^{(\mathrm{s})}\right|^2 + \left|t^{(\mathrm{p})}\right|^2 \frac{\mathrm{R}^{(\omega^\pm)}}{(1\pm\tilde{\Omega})^2\,|\varepsilon(\omega)|\,|\varepsilon(\omega^\pm)|}\left\{\left[\tilde{k}^*_{z,2}\tilde{k}^\pm_{z,2} + \kappa_R^2\right]\left[\tilde{k}_{z,2}\tilde{k}^{\pm*}_{z,2} - \kappa_R^2\right]\right\},$$

$$\tilde{\Gamma}^{(\omega^\pm)(\uparrow/\downarrow)}_{\mathrm{T-T}\,(1,1)} = \frac{e^{-2k_0\mathrm{Im}[\tilde{k}_{z,1}]z}}{2\mathrm{Im}[\tilde{k}^*_{z,2}][\tilde{k}_{z,2}-\tilde{k}^{\pm*}_{z,2}][\tilde{k}_{z,2}+\tilde{k}^\pm_{z,2}]},$$

$$\tilde{\mathcal{G}}^{(\omega^\pm)(\downarrow/\uparrow)}_{\mathrm{T-T}\,(1,1)} = \left|t^{(\mathrm{s})}\right|^2 + \left|t^{(\mathrm{p})}\right|^2 \frac{\mathrm{R}^{(\omega^\pm)}}{(1\pm\tilde{\Omega})^2\,|\varepsilon(\omega)|\,|\varepsilon(\omega^\pm)|}\left\{\left[\tilde{k}^*_{z,2}\tilde{k}^\pm_{z,2} - \kappa_R^2\right]\left[\tilde{k}_{z,2}\tilde{k}^{\pm*}_{z,2} + \kappa_R^2\right]\right\},$$

$$\tilde{\Gamma}^{(\omega^\pm)(\downarrow/\uparrow)}_{\mathrm{T-T}\,(1,1)} = \frac{e^{-2k_0\mathrm{Im}[\tilde{k}_{z,1}]z}}{2\mathrm{Im}[\tilde{k}^*_{z,2}][\tilde{k}^*_{z,2}-\tilde{k}^\pm_{z,2}][\tilde{k}^*_{z,2}+\tilde{k}^{\pm*}_{z,2}]},$$

$$\tilde{\mathcal{G}}^{(\omega^\pm)(\downarrow/\downarrow)}_{\mathrm{T-T}\,(1,1)} = \left|t^{(\mathrm{s})}\right|^2 + \left|t^{(\mathrm{p})}\right|^2 \frac{\mathrm{T}^{(\omega^\pm)}}{(1\pm\tilde{\Omega})^2\,|\varepsilon(\omega)|\,|\varepsilon(\omega^\pm)|}\left\{\left[\tilde{k}^*_{z,2}\tilde{k}^\pm_{z,2} - \kappa_R^2\right]\left[\tilde{k}_{z,2}\tilde{k}^{\pm*}_{z,2} - \kappa_R^2\right]\right\},$$

$$\tilde{\Gamma}^{(\omega^\pm)(\downarrow/\downarrow)}_{\mathrm{T-T}\,(1,1)} = \frac{e^{-2k_0\mathrm{Im}[\tilde{k}_{z,1}]z}}{2\mathrm{Im}[\tilde{k}_{z,2}]|\tilde{k}_{z,2}-\tilde{k}^{\pm*}_{z,2}|^2},$$

$$\tilde{\mathcal{G}}^{(\omega^\pm)}_{\mathrm{R-R}\,(1,1)} = \frac{1}{32\pi k_0^5}\int_0^{+\infty} d\kappa_R \frac{\kappa_R}{|\tilde{k}_{z,2}|^2|\tilde{k}^\pm_{z,2}|^2}\tilde{\mathcal{G}}^{(\omega^\pm)(\downarrow/\downarrow)}_{\mathrm{R-R}\,(1,1)}\tilde{\Gamma}^{(\omega^\pm)}_{\mathrm{R-R}\,(1,1)}, \quad \text{(S.VI.51)}$$

where

$$\tilde{\mathcal{G}}^{(\omega^\pm)(\downarrow/\downarrow)}_{\mathrm{R-R}\,(1,1)} = \left|t^{(\mathrm{s})}\right|^2\left|r^{(\mathrm{s})(\omega^\pm)}\right|^2 + \left|t^{(\mathrm{p})}\right|^2\left|r^{(\mathrm{p})(\omega^\pm)}\right|^2 \frac{\mathrm{T}^{(\omega^\pm)}}{(1\pm\tilde{\Omega})\,|\varepsilon(\omega)|\,|\varepsilon(\omega^\pm)|}\left\{\left[\tilde{k}^*_{z,2}\tilde{k}^\pm_{z,2} - \kappa_R^2\right]\left[\tilde{k}_{z,2}\tilde{k}^{\pm*}_{z,2} - \kappa_R^2\right]\right\},$$

$$\tilde{\Gamma}^{(\omega^\pm)}_{\mathrm{R-R}\,(1,1)} = \frac{e^{-2k_0\mathrm{Im}[\tilde{k}_{z,1}]z}}{2\mathrm{Im}[\tilde{k}^\pm_{z,2}]|\tilde{k}_{z,2}-\tilde{k}^{\pm*}_{z,2}|^2},$$

$$\tilde{\mathcal{G}}^{(\omega^{\pm})}_{\mathrm{T-R}\,(1,1)} = \frac{1}{32\pi k_0^5}\int_0^{+\infty} d\kappa_R \frac{\kappa_R}{|\tilde{k}_{z,2}|^2|\tilde{k}^{\pm}_{z,2}|^2}\sum_{i=\{\uparrow,\downarrow\}}\tilde{\mathcal{G}}^{(\omega^{\pm})(i/\downarrow)}_{\mathrm{T-R}\,(1,1)}\tilde{\Gamma}^{(\omega^{\pm})(i)}_{\mathrm{T-R}\,(1,1)}, \tag{S.VI.52}$$

where

$$\tilde{\mathcal{G}}^{(\omega^{\pm})(\uparrow/\downarrow)}_{\mathrm{T-R}\,(1,1)} = \left|t^{(\mathrm{s})}\right|^2 r^{(\mathrm{s})(\omega^{\pm})*} + \left|t^{(\mathrm{p})}\right|^2 r^{(\mathrm{p})(\omega^{\pm})*}\frac{\mathrm{T}^{(\omega^{\pm})}}{(1\pm\tilde{\Omega})^2\,|\varepsilon(\omega)|\,|\varepsilon(\omega^{\pm})|}\left\{\left[\tilde{k}^*_{z,2}\tilde{k}^{\pm}_{z,2}+\kappa_R^2\right]\left[\tilde{k}_{z,2}\tilde{k}^{\pm*}_{z,2}-\kappa_R^2\right]\right\},$$

$$\tilde{\Gamma}^{(\omega^{\pm})(\uparrow)}_{\mathrm{T-R}\,(1,1)} = \frac{e^{-2k_0\mathrm{Im}[\tilde{k}_{z,1}]z}}{2\mathrm{Im}[\tilde{k}^{\pm}_{z,2}][\tilde{k}_{z,2}-\tilde{k}^{\pm*}_{z,2}][\tilde{k}^*_{z,2}+\tilde{k}^{\pm*}_{z,2}]},$$

$$\tilde{\mathcal{G}}^{(\omega^{\pm})(\downarrow/\downarrow)}_{\mathrm{T-R}\,(1,1)} = \left|t^{(\mathrm{s})}\right|^2 r^{(\mathrm{s})(\omega^{\pm})*} + \left|t^{(\mathrm{p})}\right|^2 r^{(\mathrm{p})(\omega^{\pm})*}\frac{\mathrm{R}^{(\omega^{\pm})}}{(1\pm\tilde{\Omega})^2\,|\varepsilon(\omega)|\,|\varepsilon(\omega^{\pm})|}\left\{\left[\tilde{k}^*_{z,2}\tilde{k}^{\pm}_{z,2}-\kappa_R^2\right]\left[\tilde{k}_{z,2}\tilde{k}^{\pm*}_{z,2}-\kappa_R^2\right]\right\},$$

$$\tilde{\Gamma}^{(\omega^{\pm})(\downarrow)}_{\mathrm{T-R}\,(1,1)} = \frac{e^{-2k_0\mathrm{Im}[\tilde{k}_{z,1}]z}}{i|\tilde{k}_{z,2}-\tilde{k}^{\pm*}_{z,2}|^2[\tilde{k}^*_{z,2}+\tilde{k}^{\pm*}_{z,2}]}.$$

with $\mathrm{T}^{(\omega^{\pm})}$ and $\mathrm{R}^{(\omega^{\pm})}$ given, respectively, in Eq. (S.VI.41a) and (S.VI.41b).

$$\tilde{\mathcal{G}}^{(\omega^{\pm})}_{\mathrm{Z-Z}\,(1,1)} = \frac{1}{32\pi k_0^5}\int_0^{+\infty} d\kappa_R \frac{2\kappa_R^3(|\tilde{k}_{z,1}|^2+\kappa_R^2)}{|\tilde{k}_{z,2}|^2(1\pm\tilde{\Omega})^4\,|\varepsilon(\omega)|\,|\varepsilon(\omega^{\pm})|^2}\left|t^{(\mathrm{p})}\right|^2\frac{e^{-2k_0\mathrm{Im}[\tilde{k}_{z,1}]z}}{\mathrm{Im}[\tilde{k}_{z,2}]}, \tag{S.VI.53}$$

$$\tilde{\mathcal{G}}^{(\omega^{\pm})}_{\mathrm{T-Z}\,(1,1)} = \frac{1}{32\pi k_0^5}\int_0^{+\infty} d\kappa_R \frac{2\kappa_R^3(|\tilde{k}_{z,1}|^2+\kappa_R^2)}{|\tilde{k}_{z,2}|^2(1\pm\tilde{\Omega})^4\,|\varepsilon(\omega)|\,|\varepsilon(\omega^{\pm})|^2}\left|t^{(\mathrm{p})}\right|^2\sum_{i=\{\uparrow,\downarrow\}}\tilde{\mathcal{G}}^{(\omega^{\pm})(i)}_{\mathrm{T-Z}\,(1,1)}\tilde{\Gamma}^{(\omega^{\pm})(i)}_{\mathrm{T-Z}\,(1,1)}, \tag{S.VI.54}$$

where

$$\tilde{\mathcal{G}}^{(\omega^{\pm})(\uparrow)}_{\mathrm{T-Z}\,(1,1)} = \left[\tilde{k}^*_{z,2}\tilde{k}^{\pm}_{z,2}+\kappa_R^2\right],$$

$$\tilde{\Gamma}^{(\omega^{\pm})(\uparrow)}_{\mathrm{T-Z}\,(1,1)} = \frac{e^{-2k_0\mathrm{Im}[\tilde{k}_{z,1}]z}}{2\mathrm{Im}[\tilde{k}_{z,2}][\tilde{k}_{z,2}+\tilde{k}^{\pm}_{z,2}]\tilde{k}^{\pm}_{z,2}},$$

$$\tilde{\mathcal{G}}^{(\omega^{\pm})(\downarrow)}_{\mathrm{T-Z}\,(1,1)} = \left[\tilde{k}^*_{z,2}\tilde{k}^{\pm}_{z,2}-\kappa_R^2\right],$$

$$\tilde{\Gamma}^{(\omega^{\pm})(\downarrow)}_{\mathrm{T-Z}\,(1,1)} = \frac{e^{-2k_0\mathrm{Im}[\tilde{k}_{z,1}]z}}{2\mathrm{Im}[\tilde{k}_{z,2}][\tilde{k}^*_{z,2}-\tilde{k}^{\pm}_{z,2}]\tilde{k}^{\pm}_{z,2}},$$

$$\tilde{\mathcal{G}}^{(\omega^{\pm})}_{\mathrm{R-Z}\,(1,1)} = \frac{1}{32\pi k_0^5}\int_0^{+\infty} d\kappa_R \frac{2\kappa_R^3(|\tilde{k}_{z,1}|^2+\kappa_R^2)}{|\tilde{k}_{z,2}|^2(1\pm\tilde{\Omega})^4\,|\varepsilon(\omega)|\,|\varepsilon(\omega^{\pm})|^2}\left|t^{(\mathrm{p})}\right|^2\tilde{\mathcal{G}}^{(\omega^{\pm})(\downarrow)}_{\mathrm{R-Z}\,(1,1)}\tilde{\Gamma}^{(\omega^{\pm})}_{\mathrm{R-Z}\,(1,1)}, \tag{S.VI.55}$$

where

$$\tilde{\mathcal{G}}^{(\omega^{\pm})(\downarrow)}_{\mathrm{R-Z}\,(1,1)} = r^{(\mathrm{p})(\omega^{\pm})}\left[\tilde{k}^*_{z,2}\tilde{k}^{\pm}_{z,2}-\kappa_R^2\right],$$

$$\tilde{\Gamma}^{(\omega^{\pm})}_{\mathrm{R-Z}\,(1,1)} = \frac{e^{-2k_0\mathrm{Im}[\tilde{k}_{z,1}]z}}{i[\tilde{k}^*_{z,2}-\tilde{k}^{\pm}_{z,2}][\tilde{k}_{z,2}+\tilde{k}^{\pm}_{z,2}]\tilde{k}^{\pm}_{z,2}}.$$

Calculation of $\bar{\bar{\mathcal{G}}}_{0,2}^{(\omega^{\pm})}$:

Finally, we proceed with the calculation of $\bar{\bar{\mathcal{G}}}_{0,2}^{(\omega^{\pm})}$. Again, we start by checking the order of the matrices involved:

$$[\hat{E}_{0,\alpha}^{(+)}]^{\dagger}\cdot\hat{E}_{2,\alpha}^{(+)}\propto G^{*}_{\alpha,\beta}\hat{j}^{\dagger}_{0,\beta}G_{\alpha,\gamma}\hat{j}_{2,\gamma}\propto G^{*}_{\alpha,\beta}\hat{j}^{\dagger}_{0,\beta}G_{\alpha,\gamma}G_{\gamma,\delta}\hat{j}_{1,\delta}\propto G^{*}_{\alpha,\beta}G_{\alpha,\gamma}G_{\gamma,\delta}G_{\delta,\zeta}\delta_{\beta,\zeta}\propto G^{*}_{\alpha,\beta}G_{\alpha,\gamma}G_{\gamma,\delta}G_{\delta,\beta}.$$

In this way we can see that the four matrices can again be grouped in two sets, now consisting in a single matrix and an ordinary product of three matrices:

$$\begin{aligned}\mathbf{G}^{*}(\mathbf{r},\boldsymbol{\rho},\omega)\mathbf{G}(\mathbf{r},\boldsymbol{\rho}',\omega)\mathbf{G}(\boldsymbol{\rho}',\boldsymbol{\rho}'',\omega^{\pm})\mathbf{G}^{*}(\boldsymbol{\rho}'',\boldsymbol{\rho},\omega)&=G^{*}_{\alpha,\beta}(\mathbf{r},\boldsymbol{\rho},\omega)G_{\alpha,\gamma}(\mathbf{r},\boldsymbol{\rho}',\omega)G_{\gamma,\delta}(\boldsymbol{\rho}',\boldsymbol{\rho}'',\omega^{\pm})G_{\delta,\beta}(\boldsymbol{\rho}'',\boldsymbol{\rho},\omega)\\&=\bar{\bar{G}}^{*}_{\alpha,\beta}(\mathbf{r},\boldsymbol{\rho},\omega)\bar{\bar{G}}_{\alpha,\beta}(\mathbf{r},\boldsymbol{\rho},\boldsymbol{\rho}',\boldsymbol{\rho}'',\omega,\omega^{\pm}),\end{aligned}$$

with $\bar{\bar{G}}^{*}_{\alpha,\beta}(\mathbf{r},\boldsymbol{\rho},\omega)\equiv G^{*}_{\alpha,\beta}(\mathbf{r},\boldsymbol{\rho},\omega)$, $\bar{\bar{G}}_{\alpha,\beta}(\mathbf{r},\boldsymbol{\rho},\boldsymbol{\rho}',\boldsymbol{\rho}'',\omega,\omega^{\pm})\equiv G_{\alpha,\gamma}(\mathbf{r},\boldsymbol{\rho}',\omega)G_{\gamma,\delta}(\boldsymbol{\rho}',\boldsymbol{\rho}'',\omega^{\pm})G_{\delta,\beta}(\boldsymbol{\rho}'',\boldsymbol{\rho},\omega)$, and noticing, once again, that the summations over γ and δ stand for the ordinary matrix product. Inserting this latter expression into (S.VI.30), and applying the expansion given in Eq. (S.VI.1), it follows that:

$$\begin{aligned}\bar{\bar{\mathcal{G}}}_{0,2}^{(\omega^{\pm})}&=\iiint\limits_{\mathcal{V}}d^3\boldsymbol{\rho}d^3\boldsymbol{\rho}'d^3\boldsymbol{\rho}''\mathrm{Tr}[\mathbf{G}^{*}(\mathbf{r},\boldsymbol{\rho},\omega)\mathbf{G}(\mathbf{r},\boldsymbol{\rho}',\omega)\mathbf{G}(\boldsymbol{\rho}',\boldsymbol{\rho}'',\omega^{\pm})\mathbf{G}^{*}(\boldsymbol{\rho}'',\boldsymbol{\rho},\omega)]\\&=\frac{k_0^2}{4\pi^2}\iiint_{-\infty}^{0}d\rho_z d\rho_z' d\rho_z''\iint_{-\infty}^{+\infty}d^2\boldsymbol{\kappa}_{\parallel}\mathrm{Tr}[\hat{\mathbf{G}}^{*}(\kappa_x,\kappa_y;\omega|z,\rho_z)\hat{\mathbf{G}}(\kappa_x,\kappa_y;\omega|z,\rho_z')\hat{\mathbf{G}}(\kappa_x^{\pm},\kappa_y^{\pm};\omega^{\pm}|\rho_z',\rho_z'')\hat{\mathbf{G}}(\kappa_x,\kappa_y;\omega|\rho_z'',\rho_z)].\end{aligned}$$

Hence:

$$\bar{\bar{\mathcal{G}}}_{0,2}^{(\omega^{\pm})}=\frac{k_0^2}{4\pi^2}\iiint_{-\infty}^{0}d\rho_z d\rho_z' d\rho_z''\int_0^{+\infty}d\kappa_R\kappa_R\int_0^{2\pi}d\kappa_{\varphi}\left\|\left[\hat{\bar{\bar{\mathbf{G}}}}{}^{[(\uparrow/\uparrow)]}_{z\leftarrow\rho_z}\right]^{*}\hat{\bar{\bar{\mathbf{G}}}}{}^{(\omega^{\pm})[(\uparrow/\uparrow)(\updownarrow/\updownarrow)(\updownarrow/\updownarrow)]}_{z\leftarrow\rho_z'\leftarrow\rho_z''\leftarrow\rho_z}\right\|_{\mathcal{F}},\qquad\text{(S.VI.56)}$$

where

$$\left[\hat{\bar{\bar{\mathbf{G}}}}{}^{[(\uparrow/\uparrow)]}_{z\leftarrow\rho_z}\right]^{*}\equiv\left[\hat{\bar{\bar{\mathbf{G}}}}_{z\leftarrow\rho_z}\right]^{*}=\left[\hat{\mathbf{G}}^{(\uparrow/\uparrow)}(\kappa_x,\kappa_y;\omega|z,\rho_z)\right]^{*},\qquad\text{(S.VI.57a)}$$

$$\hat{\bar{\bar{\mathbf{G}}}}{}^{(\omega^{\pm})[(\uparrow/\uparrow)(\updownarrow/\updownarrow)(\updownarrow/\updownarrow)]}_{z\leftarrow\rho_z'\leftarrow\rho_z''\leftarrow\rho_z}\equiv\hat{\bar{\bar{\mathbf{G}}}}{}^{(\omega^{\pm})[(\updownarrow/\updownarrow)(\updownarrow/\updownarrow)]}_{z\leftarrow\rho_z'\leftarrow\rho_z''\leftarrow\rho_z}=\hat{\mathbf{G}}^{(\uparrow/\uparrow)}(\kappa_x,\kappa_y;\omega|z,\rho_z')\cdot\hat{\mathbf{G}}^{(\updownarrow/\updownarrow)}(\kappa_x^{\pm},\kappa_y^{\pm};\omega^{\pm}|\rho_z',\rho_z'')\cdot\hat{\mathbf{G}}^{(\updownarrow/\updownarrow)}(\kappa_x,\kappa_y;\omega|\rho_z'',\rho_z).\qquad\text{(S.VI.57b)}$$

Then, from the notation given in Eqs. (S.VI.5)–(S.VI.12d), in this case it follows that:

$$\left[\hat{\bar{\bar{\mathbf{G}}}}_{z\leftarrow\rho_z}\right]^{*}=\frac{-i}{2}\frac{1}{k_{z,2}^{*}}\left[t^{(\mathrm{s})}\hat{\mathbf{g}}^{(\mathrm{s})}+t^{(\mathrm{p})}\left(\hat{\mathbf{p}}_z^{\uparrow}\otimes\hat{\mathbf{p}}_{\rho_z}^{\uparrow}\right)\right]^{*}e^{-i\left[k_{z,1}^{*}z-k_{z,2}^{*}\rho_z\right]},$$

$$\begin{aligned}\hat{\bar{\bar{\mathbf{G}}}}{}^{(\omega^{\pm})[(\updownarrow/\updownarrow)(\updownarrow/\updownarrow)]}_{z\leftarrow\rho_z'\leftarrow\rho_z''\leftarrow\rho_z}&=\hat{\bar{\bar{\mathbf{T}\mathbf{T}}}}{}^{(\omega^{\pm})[(\updownarrow/\updownarrow)(\updownarrow/\updownarrow)]}_{z\leftarrow\rho_z'\leftarrow\rho_z''\leftarrow\rho_z}+\hat{\bar{\bar{\mathbf{T}\mathbf{R}}}}{}^{(\omega^{\pm})[(\updownarrow/\updownarrow)(\downarrow/\uparrow)]}_{z\leftarrow\rho_z'\leftarrow\rho_z''\leftarrow\rho_z}+\hat{\bar{\bar{\mathbf{T}\mathbf{Z}}}}{}^{(\omega^{\pm})[(\updownarrow/\updownarrow)]}_{z\leftarrow\rho_z'\leftarrow\rho_z''\leftarrow\rho_z}\\&+\hat{\bar{\bar{\mathbf{R}\mathbf{T}}}}{}^{(\omega^{\pm})[(\downarrow/\uparrow)(\updownarrow/\updownarrow)]}_{z\leftarrow\rho_z'\leftarrow\rho_z''\leftarrow\rho_z}+\hat{\bar{\bar{\mathbf{R}\mathbf{R}}}}{}^{(\omega^{\pm})[(\downarrow/\uparrow)(\downarrow/\uparrow)]}_{z\leftarrow\rho_z'\leftarrow\rho_z''\leftarrow\rho_z}+\hat{\bar{\bar{\mathbf{R}\mathbf{Z}}}}{}^{(\omega^{\pm})[(\downarrow/\uparrow)]}_{z\leftarrow\rho_z'\leftarrow\rho_z''\leftarrow\rho_z}\\&+\hat{\bar{\bar{\mathbf{Z}\mathbf{T}}}}{}^{(\omega^{\pm})[(\updownarrow/\updownarrow)]}_{z\leftarrow\rho_z'\leftarrow\rho_z''\leftarrow\rho_z}+\hat{\bar{\bar{\mathbf{Z}\mathbf{R}}}}{}^{(\omega^{\pm})[(\downarrow/\uparrow)]}_{z\leftarrow\rho_z'\leftarrow\rho_z''\leftarrow\rho_z}+\hat{\bar{\bar{\mathbf{Z}\mathbf{Z}}}}{}^{(\omega^{\pm})}_{z\leftarrow\rho_z'\leftarrow\rho_z''\leftarrow\rho_z},\end{aligned}$$

with

$$\hat{\bar{\bar{\mathbf{T}\mathbf{T}}}}{}^{(\omega^{\pm})[(\updownarrow/\updownarrow)(\updownarrow/\updownarrow)]}_{z\leftarrow\rho_z'\leftarrow\rho_z''\leftarrow\rho_z}=\frac{-i}{8}\frac{1}{k_{z,2}^2k_{z,2}^{\pm}}\left[t^{(\mathrm{s})}\hat{\mathbf{g}}^{(\mathrm{s})}+t^{(\mathrm{p})}\left(\hat{\mathbf{p}}_{\rho_z'}^{\uparrow}\cdot\hat{\mathbf{p}}_{\rho_z'}^{(\omega^{\pm})\updownarrow}\right)\left(\hat{\mathbf{p}}_{\rho_z''}^{(\omega^{\pm})\updownarrow}\cdot\hat{\mathbf{p}}_{\rho_z''}^{\updownarrow}\right)\left(\hat{\mathbf{p}}_z^{\uparrow}\otimes\hat{\mathbf{p}}_{\rho_z}^{\updownarrow}\right)\right]\bar{\bar{\Gamma}}{}^{(\omega^{\pm})(\updownarrow/\updownarrow)}_{\mathrm{T-T}\,(0,2)},$$

$$\hat{\bar{\bar{\mathbf{T}\mathbf{R}}}}{}^{(\omega^{\pm})[(\updownarrow/\updownarrow)(\downarrow/\uparrow)]}_{z\leftarrow\rho_z'\leftarrow\rho_z''\leftarrow\rho_z}=\frac{-i}{8}\frac{1}{k_{z,2}^2k_{z,2}^{\pm}}\left[t^{(\mathrm{s})}r^{(\mathrm{s})}\hat{\mathbf{g}}^{(\mathrm{s})}+t^{(\mathrm{p})}r^{(\mathrm{p})}\left(\hat{\mathbf{p}}_{\rho_z'}^{\uparrow}\cdot\hat{\mathbf{p}}_{\rho_z'}^{(\omega^{\pm})\updownarrow}\right)\left(\hat{\mathbf{p}}_{\rho_z''}^{(\omega^{\pm})\updownarrow}\cdot\hat{\mathbf{p}}_{\rho_z''}^{\downarrow}\right)\left(\hat{\mathbf{p}}_z^{\uparrow}\otimes\hat{\mathbf{p}}_{\rho_z}^{\uparrow}\right)\right]\bar{\bar{\Gamma}}{}^{(\omega^{\pm})(\updownarrow)}_{\mathrm{T-R}\,(0,2)},$$

$$\hat{\bar{\bar{\mathbf{T}\mathbf{Z}}}}{}^{(\omega^{\pm})[(\updownarrow/\updownarrow)]}_{z\leftarrow\rho_z'\leftarrow\rho_z''\leftarrow\rho_z}=\frac{1}{4}\frac{1}{k_{z,2}k_{z,2}^{\pm}k_0^2\varepsilon(\omega)}\left[t^{(\mathrm{p})}\left(\hat{\mathbf{p}}_{\rho_z'}^{\uparrow}\cdot\hat{\mathbf{p}}_{\rho_z'}^{(\omega^{\pm})\updownarrow}\right)\left(\hat{\mathbf{p}}_{\rho_z''}^{(\omega^{\pm})\updownarrow}\cdot\hat{\mathbf{z}}\right)\left(\hat{\mathbf{p}}_z^{\uparrow}\otimes\hat{\mathbf{z}}\right)\right]\bar{\bar{\Gamma}}{}^{(\omega^{\pm})(\updownarrow)}_{\mathrm{T-Z}\,(0,2)},$$

$$\bar{\bar{\Gamma}}^{(\omega^{\pm})(\updownarrow/\updownarrow)}_{\mathrm{T-T}\,(0,2)} = e^{i\left[k_{z,1}z - k_{z,2}\rho'_z\right]} e^{ik^{\pm}_{z,2}\left|\rho'_z-\rho''_z\right|} e^{ik_{z,2}\left|\rho''_z-\rho_z\right|},$$
$$\bar{\bar{\Gamma}}^{(\omega^{\pm})(\updownarrow)}_{\mathrm{T-R}\,(0,2)} = e^{i\left[k_{z,1}z - k_{z,2}\rho'_z\right]} e^{ik^{\pm}_{z,2}\left|\rho'_z-\rho''_z\right|} e^{-ik_{z,2}\left[\rho''_z+\rho_z\right]},$$
$$\bar{\bar{\Gamma}}^{(\omega^{\pm})(\updownarrow)}_{\mathrm{T-Z}\,(0,2)} = e^{i\left[k_{z,1}z - k_{z,2}\rho'_z\right]} e^{ik^{\pm}_{z,2}\left|\rho'_z-\rho''_z\right|} \delta[\rho''_z-\rho_z],$$

$$\mathbf{R}\bar{\bar{\mathbf{T}}}^{(\omega^{\pm})[(\downarrow/\uparrow)(\updownarrow/\updownarrow)]}_{z\leftarrow\rho'_z\leftarrow\rho''_z\leftarrow\rho_z} = \frac{-i}{8}\frac{1}{k^2_{z,2}k^{\pm}_{z,2}}\left[t^{(\mathrm{s})}r^{(\mathrm{s})(\omega^{\pm})}\hat{\mathbf{g}}^{(\mathrm{s})} + t^{(\mathrm{p})}r^{(\mathrm{p})(\omega^{\pm})}\left(\hat{\mathbf{p}}^{\uparrow}_{\rho'_z}\cdot\hat{\mathbf{p}}^{(\omega^{\pm})\downarrow}_{\rho'_z}\right)\left(\hat{\mathbf{p}}^{(\omega^{\pm})\uparrow}_{\rho''_z}\cdot\hat{\mathbf{p}}^{\updownarrow}_{\rho''_z}\right)\left(\hat{\mathbf{p}}^{\uparrow}_{z}\otimes\hat{\mathbf{p}}^{\updownarrow}_{\rho_z}\right)\right]\bar{\bar{\Gamma}}^{(\omega^{\pm})(\updownarrow)}_{\mathrm{R-T}\,(0,2)},$$
$$\mathbf{R}\bar{\bar{\mathbf{R}}}^{(\omega^{\pm})[(\downarrow/\uparrow)(\downarrow/\uparrow)]}_{z\leftarrow\rho'_z\leftarrow\rho''_z\leftarrow\rho_z} = \frac{-i}{8}\frac{1}{k^2_{z,2}k^{\pm}_{z,2}}\left[t^{(\mathrm{s})}r^{(\mathrm{s})(\omega^{\pm})}r^{(\mathrm{s})}\hat{\mathbf{g}}^{(\mathrm{s})} + t^{(\mathrm{p})}r^{(\mathrm{p})(\omega^{\pm})}r^{(\mathrm{p})}\left(\hat{\mathbf{p}}^{\uparrow}_{\rho'_z}\cdot\hat{\mathbf{p}}^{(\omega^{\pm})\downarrow}_{\rho'_z}\right)\left(\hat{\mathbf{p}}^{(\omega^{\pm})\uparrow}_{\rho''_z}\cdot\hat{\mathbf{p}}^{\downarrow}_{\rho''_z}\right)\left(\hat{\mathbf{p}}^{\uparrow}_{z}\otimes\hat{\mathbf{p}}^{\uparrow}_{\rho_z}\right)\right]\bar{\bar{\Gamma}}^{(\omega^{\pm})}_{\mathrm{R-R}\,(0,2)},$$
$$\mathbf{R}\bar{\bar{\mathbf{Z}}}^{(\omega^{\pm})[(\downarrow/\uparrow)]}_{z\leftarrow\rho'_z\leftarrow\rho''_z\leftarrow\rho_z} = \frac{1}{4}\frac{1}{k_{z,2}k^{\pm}_{z,2}k^2_0\varepsilon(\omega)}\left[t^{(\mathrm{p})}r^{(\mathrm{p})(\omega^{\pm})}\left(\hat{\mathbf{p}}^{\uparrow}_{\rho'_z}\cdot\hat{\mathbf{p}}^{(\omega^{\pm})\downarrow}_{\rho'_z}\right)\left(\hat{\mathbf{p}}^{(\omega^{\pm})\uparrow}_{\rho''_z}\cdot\hat{\mathbf{z}}\right)\left(\hat{\mathbf{p}}^{\uparrow}_{z}\otimes\hat{\mathbf{z}}\right)\right]\bar{\bar{\Gamma}}^{(\omega^{\pm})}_{\mathrm{R-Z}\,(0,2)},$$

$$\bar{\bar{\Gamma}}^{(\omega^{\pm})(\updownarrow)}_{\mathrm{R-T}\,(0,2)} = e^{i\left[k_{z,1}z - k_{z,2}\rho'_z\right]} e^{-ik^{\pm}_{z,2}\left[\rho'_z+\rho''_z\right]} e^{ik_{z,2}\left|\rho''_z-\rho_z\right|},$$
$$\bar{\bar{\Gamma}}^{(\omega^{\pm})}_{\mathrm{R-R}\,(0,2)} = e^{i\left[k_{z,1}z - k_{z,2}\rho'_z\right]} e^{-ik^{\pm}_{z,2}\left[\rho'_z+\rho''_z\right]} e^{-ik_{z,2}\left[\rho''_z+\rho_z\right]},$$
$$\bar{\bar{\Gamma}}^{(\omega^{\pm})}_{\mathrm{R-Z}\,(0,2)} = e^{i\left[k_{z,1}z - k_{z,2}\rho'_z\right]} e^{-ik^{\pm}_{z,2}\left[\rho'_z+\rho''_z\right]} \delta[\rho''_z-\rho_z],$$

$$\mathbf{Z}\bar{\bar{\mathbf{T}}}^{(\omega^{\pm})[(\updownarrow/\updownarrow)]}_{z\leftarrow\rho'_z\leftarrow\rho''_z\leftarrow\rho_z} = \frac{1}{4}\frac{1}{k^2_{z,2}k^2_0(1\pm\tilde{\Omega})^2\varepsilon(\omega^{\pm})}\left[t^{(\mathrm{p})}\left(\hat{\mathbf{p}}^{\uparrow}_{\rho'_z}\cdot\hat{\mathbf{z}}\right)\left(\hat{\mathbf{z}}\cdot\hat{\mathbf{p}}^{\updownarrow}_{\rho''_z}\right)\left(\hat{\mathbf{p}}^{\uparrow}_{z}\otimes\hat{\mathbf{p}}^{\updownarrow}_{\rho_z}\right)\right]\bar{\bar{\Gamma}}^{(\omega^{\pm})(\updownarrow)}_{\mathrm{Z-T}\,(0,2)},$$
$$\mathbf{Z}\bar{\bar{\mathbf{R}}}^{(\omega^{\pm})[(\downarrow/\uparrow)]}_{z\leftarrow\rho'_z\leftarrow\rho''_z\leftarrow\rho_z} = \frac{1}{4}\frac{1}{k^2_{z,2}k^2_0(1\pm\tilde{\Omega})^2\varepsilon(\omega^{\pm})}\left[t^{(\mathrm{p})}r^{(\mathrm{p})}\left(\hat{\mathbf{p}}^{\uparrow}_{\rho'_z}\cdot\hat{\mathbf{z}}\right)\left(\hat{\mathbf{z}}\cdot\hat{\mathbf{p}}^{\downarrow}_{\rho''_z}\right)\left(\hat{\mathbf{p}}^{\uparrow}_{z}\otimes\hat{\mathbf{p}}^{\uparrow}_{\rho_z}\right)\right]\bar{\bar{\Gamma}}^{(\omega^{\pm})}_{\mathrm{Z-R}\,(0,2)},$$
$$\mathbf{Z}\bar{\bar{\mathbf{Z}}}^{(\omega^{\pm})}_{z\leftarrow\rho'_z\leftarrow\rho''_z\leftarrow\rho_z} = \frac{i}{2}\frac{1}{k_{z,2}k^4_0(1\pm\tilde{\Omega})^2\varepsilon(\omega)\varepsilon(\omega^{\pm})}\left[t^{(\mathrm{p})}\left(\hat{\mathbf{p}}^{\uparrow}_{\rho'_z}\cdot\hat{\mathbf{z}}\right)\left(\hat{\mathbf{z}}\cdot\hat{\mathbf{z}}\right)\left(\hat{\mathbf{p}}^{\uparrow}_{z}\otimes\hat{\mathbf{z}}\right)\right]\bar{\bar{\Gamma}}^{(\omega^{\pm})}_{\mathrm{Z-Z}\,(0,2)},$$

$$\bar{\bar{\Gamma}}^{(\omega^{\pm})(\updownarrow)}_{\mathrm{Z-T}\,(0,2)} = e^{i\left[k_{z,1}z - k_{z,2}\rho'_z\right]} \delta[\rho'_z-\rho''_z] e^{ik_{z,2}\left|\rho''_z-\rho_z\right|},$$
$$\bar{\bar{\Gamma}}^{(\omega^{\pm})}_{\mathrm{Z-R}\,(0,2)} = e^{i\left[k_{z,1}z - k_{z,2}\rho'_z\right]} \delta[\rho'_z-\rho''_z] e^{-ik_{z,2}\left[\rho''_z+\rho_z\right]},$$
$$\bar{\bar{\Gamma}}^{(\omega^{\pm})}_{\mathrm{Z-Z}\,(0,2)} = e^{i\left[k_{z,1}z - k_{z,2}\rho'_z\right]} \delta[\rho'_z-\rho''_z] \delta[\rho''_z-\rho_z].$$

According to the above expressions, we rewrite Eq. (S.VI.56) in the following way:

$$\bar{\bar{\mathcal{G}}}^{(\omega^{\pm})}_{0,2} = \bar{\bar{\mathcal{G}}}^{(\omega^{\pm})}_{\mathrm{T-T}\,(0,2)} + \bar{\bar{\mathcal{G}}}^{(\omega^{\pm})}_{\mathrm{R-R}\,(0,2)} + \bar{\bar{\mathcal{G}}}^{(\omega^{\pm})}_{\mathrm{T-R}\,(0,2)} + \bar{\bar{\mathcal{G}}}^{(\omega^{\pm})}_{\mathrm{R-T}\,(0,2)} + \bar{\bar{\mathcal{G}}}^{(\omega^{\pm})}_{\mathrm{Z-Z}\,(0,2)} + \bar{\bar{\mathcal{G}}}^{(\omega^{\pm})}_{\mathrm{T-Z}\,(0,2)} + \bar{\bar{\mathcal{G}}}^{(\omega^{\pm})}_{\mathrm{Z-T}\,(0,2)} + \bar{\bar{\mathcal{G}}}^{(\omega^{\pm})}_{\mathrm{R-Z}\,(0,2)} + \bar{\bar{\mathcal{G}}}^{(\omega^{\pm})}_{\mathrm{Z-R}\,(0,2)}. \quad \text{(S.VI.58)}$$

Now, we will proceed with the calculation of Eq. (S.VI.58) by analyzing separately each of the contributions (cf. Appendix VI.C for the demonstration of the p-like contribution to the (T-T)-term):

$$\bar{\bar{\mathcal{G}}}^{(\omega^{\pm})}_{\mathrm{T-T}\,(0,2)} = \frac{1}{32\pi k^5_0}\int_0^{+\infty} d\kappa_R \frac{-\kappa_R}{|\tilde{k}_{z,2}|^2\tilde{k}_{z,2}\tilde{k}^{\pm}_{z,2}} \sum_{i,j=\{\uparrow,\downarrow\}} \bar{\bar{\mathcal{G}}}^{(\omega^{\pm})(i/j)}_{\mathrm{T-T}\,(0,2)}\bar{\bar{\Gamma}}^{(\omega^{\pm})(i/j)}_{\mathrm{T-T}\,(0,2)}, \quad \text{(S.VI.59)}$$

where

$$\bar{\bar{\mathcal{G}}}^{(\omega^{\pm})(\uparrow/\uparrow)}_{\mathrm{T-T}\,(0,2)} = \left|t^{(\mathrm{s})}\right|^2 + \left|t^{(\mathrm{p})}\right|^2 \frac{\mathrm{T}}{(1\pm\tilde{\Omega})^2\varepsilon(\omega)\varepsilon(\omega^{\pm})}\left[\tilde{k}_{z,2}\tilde{k}^{\pm}_{z,2}+\kappa^2_R\right]\left[\tilde{k}_{z,2}\tilde{k}^{\pm}_{z,2}+\kappa^2_R\right],$$
$$\bar{\bar{\Gamma}}^{(\omega^{\pm})(\uparrow/\uparrow)}_{\mathrm{T-T}\,(0,2)} = \frac{e^{-2k_0\mathrm{Im}[\tilde{k}_{z,1}]z}}{4i[\mathrm{Im}[\tilde{k}_{z,2}]]^2[\tilde{k}^{*}_{z,2}-\tilde{k}^{\pm}_{z,2}]},$$

$$\bar{\bar{\mathcal{G}}}^{(\omega^{\pm})(\uparrow/\downarrow)}_{\mathrm{T-T}\,(0,2)} = \left|t^{(\mathrm{s})}\right|^2 + \left|t^{(\mathrm{p})}\right|^2 \frac{\mathrm{R}}{(1\pm\tilde{\Omega})^2\varepsilon(\omega)\varepsilon(\omega^{\pm})} \left[\tilde{k}_{z,2}\tilde{k}^{\pm}_{z,2} + \kappa_R^2\right] \left[\tilde{k}_{z,2}\tilde{k}^{\pm}_{z,2} - \kappa_R^2\right],$$

$$\bar{\bar{\Gamma}}^{(\omega^{\pm})(\uparrow/\downarrow)}_{\mathrm{T-T}\,(0,2)} = \frac{e^{-2k_0\mathrm{Im}[\tilde{k}_{z,1}]z}}{4[\tilde{k}_{z,2} - \tilde{k}^{\pm}_{z,2}]} \left[\frac{4i}{[\tilde{k}_{z,2} + \tilde{k}^{\pm}_{z,2}][\tilde{k}^{*}_{z,2} - \tilde{k}^{\pm}_{z,2}]} + \frac{1}{\tilde{k}_{z,2}\mathrm{Im}[\tilde{k}_{z,2}]}\right],$$

$$\bar{\bar{\mathcal{G}}}^{(\omega^{\pm})(\downarrow/\uparrow)}_{\mathrm{T-T}\,(0,2)} = \left|t^{(\mathrm{s})}\right|^2 + \left|t^{(\mathrm{p})}\right|^2 \frac{\mathrm{T}}{(1\pm\tilde{\Omega})^2\varepsilon(\omega)\varepsilon(\omega^{\pm})} \left[\tilde{k}_{z,2}\tilde{k}^{\pm}_{z,2} - \kappa_R^2\right] \left[\tilde{k}_{z,2}\tilde{k}^{\pm}_{z,2} - \kappa_R^2\right],$$

$$\bar{\bar{\Gamma}}^{(\omega^{\pm})(\downarrow/\uparrow)}_{\mathrm{T-T}\,(0,2)} = \frac{e^{-2k_0\mathrm{Im}[\tilde{k}_{z,1}]z}}{4i\mathrm{Im}[\tilde{k}_{z,2}]\mathrm{Im}[\tilde{k}^{*}_{z,2}][\tilde{k}_{z,2} + \tilde{k}^{\pm}_{z,2}]},$$

$$\bar{\bar{\mathcal{G}}}^{(\omega^{\pm})(\downarrow/\downarrow)}_{\mathrm{T-T}\,(0,2)} = \left|t^{(\mathrm{s})}\right|^2 + \left|t^{(\mathrm{p})}\right|^2 \frac{\mathrm{R}}{(1\pm\tilde{\Omega})^2\varepsilon(\omega)\varepsilon(\omega^{\pm})} \left[\tilde{k}_{z,2}\tilde{k}^{\pm}_{z,2} - \kappa_R^2\right] \left[\tilde{k}_{z,2}\tilde{k}^{\pm}_{z,2} + \kappa_R^2\right],$$

$$\bar{\bar{\Gamma}}^{(\omega^{\pm})(\downarrow/\downarrow)}_{\mathrm{T-T}\,(0,2)} = \frac{e^{-2k_0\mathrm{Im}[\tilde{k}_{z,1}]z}}{4\mathrm{Im}[\tilde{k}^{*}_{z,2}][\tilde{k}_{z,2} + \tilde{k}^{\pm}_{z,2}]k_{z,2}},$$

with

$$\mathrm{T} = \frac{1}{|\varepsilon(\omega)|}\left\{|\tilde{k}_{z,1}|^2|\tilde{k}_{z,2}|^2 + \kappa_R^2\left[|\tilde{k}_{z,2}|^2 + |\tilde{k}_{z,1}|^2 + \kappa_R^2\right]\right\}, \tag{S.VI.60a}$$

$$\mathrm{R} = \frac{1}{|\varepsilon(\omega)|}\left\{|\tilde{k}_{z,1}|^2|\tilde{k}_{z,2}|^2 + \kappa_R^2\left[|\tilde{k}_{z,2}|^2 - |\tilde{k}_{z,1}|^2 - \kappa_R^2\right]\right\}, \tag{S.VI.60b}$$

$$\bar{\bar{\mathcal{G}}}^{(\omega^{\pm})}_{\mathrm{R-R}\,(0,2)} = \frac{1}{32\pi k_0^5}\int_0^{+\infty} d\kappa_R \frac{-\kappa_R}{|\tilde{k}_{z,2}|^2\tilde{k}_{z,2}\tilde{k}^{\pm}_{z,2}} \bar{\bar{\mathcal{G}}}^{(\omega^{\pm})(\downarrow/\uparrow)}_{\mathrm{R-R}\,(0,2)} \bar{\bar{\Gamma}}^{(\omega^{\pm})}_{\mathrm{R-R}\,(0,2)}, \tag{S.VI.61}$$

where

$$\bar{\bar{\mathcal{G}}}^{(\omega^{\pm})(\downarrow/\uparrow)}_{\mathrm{R-R}\,(0,2)} = \left|t^{(\mathrm{s})}\right|^2 r^{(\mathrm{s})(\omega^{\pm})}r^{(\mathrm{s})} + \left|t^{(\mathrm{p})}\right|^2 r^{(\mathrm{p})(\omega^{\pm})}r^{(\mathrm{p})} \frac{\mathrm{T}}{(1\pm\tilde{\Omega})^2\varepsilon(\omega)\varepsilon(\omega^{\pm})} \left[\tilde{k}_{z,2}\tilde{k}^{\pm}_{z,2} - \kappa_R^2\right] \left[\tilde{k}_{z,2}\tilde{k}^{\pm}_{z,2} - \kappa_R^2\right],$$

$$\bar{\bar{\Gamma}}^{(\omega^{\pm})}_{\mathrm{R-R}\,(0,2)} = \frac{e^{-2k_0\mathrm{Im}[\tilde{k}_{z,1}]z}}{2\mathrm{Im}[\tilde{k}^{*}_{z,2}][\tilde{k}_{z,2} + \tilde{k}^{\pm}_{z,2}]^2},$$

$$\bar{\bar{\mathcal{G}}}^{(\omega^{\pm})}_{\mathrm{T-R}\,(0,2)} = \frac{1}{32\pi k_0^5}\int_0^{+\infty} d\kappa_R \frac{-\kappa_R}{|\tilde{k}_{z,2}|^2\tilde{k}_{z,2}\tilde{k}^{\pm}_{z,2}} \sum_{i=\{\uparrow,\downarrow\}} \bar{\bar{\mathcal{G}}}^{(\omega^{\pm})(i/\uparrow)}_{\mathrm{T-R}\,(0,2)} \bar{\bar{\Gamma}}^{(\omega^{\pm})(i)}_{\mathrm{T-R}\,(0,2)}, \tag{S.VI.62}$$

where

$$\bar{\bar{\mathcal{G}}}^{(\omega^{\pm})(\uparrow/\uparrow)}_{\mathrm{T-R}\,(0,2)} = \left|t^{(\mathrm{s})}\right|^2 r^{(\mathrm{s})} + \left|t^{(\mathrm{p})}\right|^2 r^{(\mathrm{p})} \frac{\mathrm{T}}{(1\pm\tilde{\Omega})^2\varepsilon(\omega)\varepsilon(\omega^{\pm})} \left[\tilde{k}_{z,2}\tilde{k}^{\pm}_{z,2} + \kappa_R^2\right] \left[\tilde{k}_{z,2}\tilde{k}^{\pm}_{z,2} - \kappa_R^2\right],$$

$$\bar{\bar{\Gamma}}^{(\omega^{\pm})(\uparrow)}_{\mathrm{T-R}\,(0,2)} = \frac{e^{-2k_0\mathrm{Im}[\tilde{k}_{z,1}]z}}{4\mathrm{Im}[\tilde{k}^{*}_{z,2}][\tilde{k}_{z,2} + \tilde{k}^{\pm}_{z,2}]\tilde{k}_{z,2}},$$

$$\bar{\bar{\mathcal{G}}}^{(\omega^{\pm})(\downarrow/\uparrow)}_{\mathrm{T-R}\,(0,2)} = \left|t^{(\mathrm{s})}\right|^2 r^{(\mathrm{s})} + \left|t^{(\mathrm{p})}\right|^2 r^{(\mathrm{p})} \frac{\mathrm{T}}{(1\pm\tilde{\Omega})^2\varepsilon(\omega)\varepsilon(\omega^{\pm})} \left[\tilde{k}_{z,2}\tilde{k}^{\pm}_{z,2} - \kappa_R^2\right] \left[\tilde{k}_{z,2}\tilde{k}^{\pm}_{z,2} + \kappa_R^2\right],$$

$$\bar{\bar{\Gamma}}^{(\omega^{\pm})(\downarrow)}_{\mathrm{T-R}\,(0,2)} = \frac{e^{-2k_0\mathrm{Im}[\tilde{k}_{z,1}]z}}{4\mathrm{Im}[\tilde{k}^{*}_{z,2}][\tilde{k}_{z,2} + \tilde{k}^{\pm}_{z,2}]\tilde{k}_{z,2}},$$

$$\bar{\bar{\mathcal{G}}}^{(\omega^\pm)}_{\mathrm{R-T}\,(0,2)} = \frac{1}{32\pi k_0^5}\int_0^{+\infty} d\kappa_R \frac{-\kappa_R}{|\tilde{k}_{z,2}|^2\tilde{k}_{z,2}\tilde{k}^{\pm}_{z,2}} \sum_{i=\{\uparrow,\downarrow\}} \bar{\bar{\mathcal{G}}}^{(\omega^\pm)(\downarrow/i)}_{\mathrm{R-T}\,(0,2)} \bar{\bar{\Gamma}}^{(\omega^\pm)(i)}_{\mathrm{R-T}\,(0,2)}, \tag{S.VI.63}$$

where

$$\bar{\bar{\mathcal{G}}}^{(\omega^\pm)(\downarrow/\uparrow)}_{\mathrm{R-T}\,(0,2)} = \left|t^{(\mathrm{s})}\right|^2 r^{(\mathrm{s})(\omega^\pm)} + \left|t^{(\mathrm{p})}\right|^2 r^{(\mathrm{p})(\omega^\pm)} \frac{\mathrm{T}}{(1\pm\tilde{\Omega})^2\varepsilon(\omega)\varepsilon(\omega^\pm)} \left[\tilde{k}_{z,2}\tilde{k}^{\pm}_{z,2} - \kappa_R^2\right]\left[\tilde{k}_{z,2}\tilde{k}^{\pm}_{z,2} + \kappa_R^2\right],$$

$$\bar{\bar{\Gamma}}^{(\omega^\pm)(\uparrow)}_{\mathrm{R-T}\,(0,2)} = \frac{e^{-2k_0\mathrm{Im}[\tilde{k}_{z,1}]z}}{2\mathrm{Im}[\tilde{k}_{z,2}][\tilde{k}_{z,2}+\tilde{k}^{\pm}_{z,2}][\tilde{k}^*_{z,2}-\tilde{k}^{\pm}_{z,2}]},$$

$$\bar{\bar{\mathcal{G}}}^{(\omega^\pm)(\downarrow/\downarrow)}_{\mathrm{R-T}\,(0,2)} = \left|t^{(\mathrm{s})}\right|^2 r^{(\mathrm{s})(\omega^\pm)} + \left|t^{(\mathrm{p})}\right|^2 r^{(\mathrm{p})(\omega^\pm)} \frac{\mathrm{R}}{(1\pm\tilde{\Omega})^2\varepsilon(\omega)\varepsilon(\omega^\pm)} \left[\tilde{k}_{z,2}\tilde{k}^{\pm}_{z,2} - \kappa_R^2\right]\left[\tilde{k}_{z,2}\tilde{k}^{\pm}_{z,2} - \kappa_R^2\right],$$

$$\bar{\bar{\Gamma}}^{(\omega^\pm)(\downarrow)}_{\mathrm{R-T}\,(0,2)} = \frac{e^{-2k_0\mathrm{Im}[\tilde{k}_{z,1}]z}}{i[\tilde{k}_{z,2}+\tilde{k}^{\pm}_{z,2}]^2[\tilde{k}^{\pm}_{z,2}-\tilde{k}^*_{z,2}]},$$

$$\bar{\bar{\mathcal{G}}}^{(\omega^\pm)}_{\mathrm{Z-Z}\,(0,2)} = \frac{1}{32\pi k_0^5}\int_0^{+\infty} d\kappa_R \frac{2\kappa_R^3(|\tilde{k}_{z,1}|^2+\kappa_R^2)}{|\tilde{k}_{z,2}|^2(1\pm\tilde{\Omega})^2\,|\varepsilon(\omega)|\,\varepsilon(\omega)\varepsilon(\omega^\pm)} \left|t^{(\mathrm{p})}\right|^2 \frac{e^{-2k_0\mathrm{Im}[\tilde{k}_{z,1}]z}}{\mathrm{Im}[\tilde{k}_{z,2}]}, \tag{S.VI.64}$$

$$\bar{\bar{\mathcal{G}}}^{(\omega^\pm)}_{\mathrm{T-Z}\,(0,2)} = \frac{1}{32\pi k_0^5}\int_0^{+\infty} d\kappa_R \frac{2\kappa_R^3(|\tilde{k}_{z,1}|^2+\kappa_R^2)}{|\tilde{k}_{z,2}|^2(1\pm\tilde{\Omega})^2\,|\varepsilon(\omega)|\,\varepsilon(\omega)\varepsilon(\omega^\pm)} \left|t^{(\mathrm{p})}\right|^2 \sum_{i=\{\uparrow,\downarrow\}} \bar{\bar{\mathcal{G}}}^{(\omega^\pm)(i)}_{\mathrm{T-Z}\,(0,2)} \bar{\bar{\Gamma}}^{(\omega^\pm)(i)}_{\mathrm{T-Z}\,(0,2)}, \tag{S.VI.65}$$

where

$$\bar{\bar{\mathcal{G}}}^{(\omega^\pm)(\uparrow)}_{\mathrm{T-Z}\,(0,2)} = \left[\tilde{k}_{z,2}\tilde{k}^{\pm}_{z,2} + \kappa_R^2\right],$$

$$\bar{\bar{\Gamma}}^{(\omega^\pm)(\uparrow)}_{\mathrm{T-Z}\,(0,2)} = \frac{e^{-2k_0\mathrm{Im}[\tilde{k}_{z,1}]z}}{2\mathrm{Im}[\tilde{k}^*_{z,2}][\tilde{k}^*_{z,2}-\tilde{k}^{\pm}_{z,2}]\tilde{k}^{\pm}_{z,2}},$$

$$\bar{\bar{\mathcal{G}}}^{(\omega^\pm)(\downarrow)}_{\mathrm{T-Z}\,(0,2)} = \left[\tilde{k}_{z,2}\tilde{k}^{\pm}_{z,2} - \kappa_R^2\right],$$

$$\bar{\bar{\Gamma}}^{(\omega^\pm)(\downarrow)}_{\mathrm{T-Z}\,(0,2)} = \frac{e^{-2k_0\mathrm{Im}[\tilde{k}_{z,1}]z}}{2\mathrm{Im}[\tilde{k}^*_{z,2}][\tilde{k}_{z,2}+\tilde{k}^{\pm}_{z,2}]\tilde{k}^{\pm}_{z,2}},$$

$$\bar{\bar{\mathcal{G}}}^{(\omega^\pm)}_{\mathrm{Z-T}\,(0,2)} = \frac{1}{32\pi k_0^5}\int_0^{+\infty} d\kappa_R \frac{2\kappa_R^3}{|\tilde{k}_{z,2}|^2(1\pm\tilde{\Omega})^2\sqrt{\varepsilon(\omega)}\sqrt{\varepsilon(\omega^\pm)}\varepsilon(\omega^\pm)} \left|t^{(\mathrm{p})}\right|^2 \sum_{i=\{\uparrow,\downarrow\}} \bar{\bar{\mathcal{G}}}^{(\omega^\pm)(i)}_{\mathrm{Z-T}\,(0,2)} \bar{\bar{\Gamma}}^{(\omega^\pm)(i)}_{\mathrm{Z-T}\,(0,2)}, \tag{S.VI.66}$$

where

$$\bar{\bar{\mathcal{G}}}^{(\omega^\pm)(\uparrow)}_{\mathrm{Z-T}\,(0,2)} = \frac{\mathrm{T}}{(1\pm\tilde{\Omega})},$$

$$\bar{\bar{\Gamma}}^{(\omega^\pm)(\uparrow)}_{\mathrm{Z-T}\,(0,2)} = \frac{e^{-2k_0\mathrm{Im}[\tilde{k}_{z,1}]z}}{4i[\mathrm{Im}[\tilde{k}_{z,2}]]^2\tilde{k}_{z,2}},$$

$$\bar{\bar{\mathcal{G}}}^{(\omega^{\pm})(\downarrow)}_{\mathrm{Z-T}\,(0,2)} = \frac{\mathrm{R}}{(1 \pm \tilde{\Omega})},$$
$$\bar{\bar{\Gamma}}^{(\omega^{\pm})(\downarrow)}_{\mathrm{Z-T}\,(0,2)} = \frac{e^{-2k_0 \mathrm{Im}[\tilde{k}_{z,1}]z}}{4\mathrm{Im}[\tilde{k}^*_{z,2}]\tilde{k}^2_{z,2}},$$

$$\bar{\bar{\mathcal{G}}}^{(\omega^{\pm})}_{\mathrm{R-Z}\,(0,2)} = \frac{1}{32\pi k_0^5} \int_0^{+\infty} d\kappa_R \frac{2\kappa_R^3(|\tilde{k}_{z,1}|^2 + \kappa_R^2)}{|\tilde{k}_{z,2}|^2(1 \pm \tilde{\Omega})^2\,|\varepsilon(\omega)|\,\varepsilon(\omega)\varepsilon(\omega^{\pm})} \left|t^{(\mathrm{p})}\right|^2 \frac{r^{(\mathrm{p})(\omega^{\pm})}(\tilde{k}_{z,2}\tilde{k}^{\pm}_{z,2} - \kappa_R^2)e^{-2k_0\mathrm{Im}[\tilde{k}_{z,1}]z}}{i[\tilde{k}_{z,2} + \tilde{k}^{\pm}_{z,2}][\tilde{k}^*_{z,2} - \tilde{k}^{\pm}_{z,2}]\tilde{k}^{\pm}_{z,2}}, \quad \text{(S.VI.67)}$$

$$\bar{\bar{\mathcal{G}}}^{(\omega^{\pm})}_{\mathrm{Z-R}\,(0,2)} = \frac{1}{32\pi k_0^5} \int_0^{+\infty} d\kappa_R \frac{2\kappa_R^3}{|\tilde{k}_{z,2}|^2(1 \pm \tilde{\Omega})^2\varepsilon(\omega)\varepsilon(\omega^{\pm})} \left|t^{(\mathrm{p})}\right|^2 \frac{r^{(\mathrm{p})}\mathrm{T}e^{-2k_0\mathrm{Im}[\tilde{k}_{z,1}]z}}{4\mathrm{Im}[\tilde{k}^*_{z,2}]\tilde{k}^2_{z,2}}. \quad \text{(S.VI.68)}$$

APPENDIX

Appendix VI.A: Calculation of Frobenius inner product associated to $\mathcal{G}^{(\omega^{\pm})}_{1,1}$

$$\begin{aligned}
\left\| \left[\hat{\bar{\mathbf{G}}}^{(\omega^{\pm})[(\uparrow/\uparrow)(\updownarrow/\updownarrow)]}_{z\leftarrow\rho_z\leftarrow\rho''_z}\right]^* \hat{\bar{\mathbf{G}}}^{(\omega^{\pm})[(\uparrow/\uparrow)(\updownarrow/\updownarrow)]}_{z\leftarrow\rho'_z\leftarrow\rho''_z} \right\|^{(\mathrm{p})}_{\mathcal{F}} &\propto \left\| \hat{\mathbf{g}}^{(\mathrm{p})(\uparrow/\uparrow)*}_{z\leftarrow\rho_z} \hat{\mathbf{g}}^{(\mathrm{p})(\omega^{\pm})(\updownarrow/\updownarrow)*}_{\rho_z\leftarrow\rho''_z} \hat{\mathbf{g}}^{(\mathrm{p})(\uparrow/\uparrow)}_{z\leftarrow\rho'_z} \hat{\mathbf{g}}^{(\mathrm{p})(\omega^{\pm})(\updownarrow/\updownarrow)}_{\rho'_z\leftarrow\rho''_z} \right\|_{\mathcal{F}} \\
&= \left\| \left(\hat{\mathbf{p}}^{\uparrow}_z \otimes \hat{\mathbf{p}}^{\uparrow}_{\rho_z}\right)^* \left(\hat{\mathbf{p}}^{(\omega^{\pm})\updownarrow}_{\rho_z} \otimes \hat{\mathbf{p}}^{(\omega^{\pm})\updownarrow}_{\rho''_z}\right)^* \left(\hat{\mathbf{p}}^{\uparrow}_z \otimes \hat{\mathbf{p}}^{\uparrow}_{\rho'_z}\right) \left(\hat{\mathbf{p}}^{(\omega^{\pm})\updownarrow}_{\rho'_z} \otimes \hat{\mathbf{p}}^{(\omega^{\pm})\updownarrow}_{\rho''_z}\right) \right\|_{\mathcal{F}} \\
&= \left(\hat{\mathbf{p}}^{\uparrow}_{\rho_z} \cdot \hat{\mathbf{p}}^{(\omega^{\pm})\updownarrow}_{\rho_z}\right)^* \left(\hat{\mathbf{p}}^{\uparrow}_{\rho'_z} \cdot \hat{\mathbf{p}}^{(\omega^{\pm})\updownarrow}_{\rho'_z}\right) \left(\left[\hat{\mathbf{p}}^{\uparrow}_z\right]^* \cdot \hat{\mathbf{p}}^{\uparrow}_z\right) \left(\left[\hat{\mathbf{p}}^{(\omega^{\pm})\updownarrow}_{\rho''_z}\right]^* \cdot \hat{\mathbf{p}}^{(\omega^{\pm})\updownarrow}_{\rho''_z}\right).
\end{aligned}$$

Appendix VI.B: Calculation of Frobenius inner product associated to $\tilde{\mathcal{G}}^{(\omega^{\pm})}_{1,1}$

$$\begin{aligned}
\left\| \left|\hat{\tilde{\mathbf{G}}}^{(\omega^{\pm})[(\uparrow/\uparrow)(\updownarrow/\updownarrow)]}_{z\leftarrow\rho_z\leftarrow\rho''_z}\right| \left|\hat{\tilde{\mathbf{G}}}^{(\omega^{\pm})[(\uparrow/\uparrow)(\updownarrow/\updownarrow)]}_{z\leftarrow\rho'_z\leftarrow\rho''_z}\right| \right\|^{(\mathrm{p})}_{\mathcal{F}} &\propto \left\| \hat{\mathbf{g}}^{(\mathrm{p})(\uparrow/\uparrow)*}_{z\leftarrow\rho_z} \hat{\mathbf{g}}^{(\mathrm{p})(\omega^{\pm})(\updownarrow/\updownarrow)}_{\rho_z\leftarrow\rho''_z} \hat{\mathbf{g}}^{(\mathrm{p})(\uparrow/\uparrow)}_{z\leftarrow\rho'_z} \hat{\mathbf{g}}^{(\mathrm{p})(\omega^{\pm})(\updownarrow/\updownarrow)*}_{\rho'_z\leftarrow\rho''_z} \right\|_{\mathcal{F}} \\
&= \left\| \left(\hat{\mathbf{p}}^{\uparrow}_z \otimes \hat{\mathbf{p}}^{\uparrow}_{\rho_z}\right)^* \left(\hat{\mathbf{p}}^{(\omega^{\pm})\updownarrow}_{\rho_z} \otimes \hat{\mathbf{p}}^{(\omega^{\pm})\updownarrow}_{\rho''_z}\right) \left(\hat{\mathbf{p}}^{\uparrow}_z \otimes \hat{\mathbf{p}}^{\uparrow}_{\rho'_z}\right) \left(\hat{\mathbf{p}}^{(\omega^{\pm})\updownarrow}_{\rho'_z} \otimes \hat{\mathbf{p}}^{(\omega^{\pm})\updownarrow}_{\rho''_z}\right)^* \right\|_{\mathcal{F}} \\
&= \left(\left[\hat{\mathbf{p}}^{\uparrow}_{\rho_z}\right]^* \cdot \hat{\mathbf{p}}^{(\omega^{\pm})\updownarrow}_{\rho_z}\right) \left(\hat{\mathbf{p}}^{\uparrow}_{\rho'_z} \cdot \left[\hat{\mathbf{p}}^{(\omega^{\pm})\updownarrow}_{\rho'_z}\right]^*\right) \left(\left[\hat{\mathbf{p}}^{\uparrow}_z\right]^* \cdot \hat{\mathbf{p}}^{\uparrow}_z\right) \left(\hat{\mathbf{p}}^{(\omega^{\pm})\updownarrow}_{\rho''_z} \cdot \left[\hat{\mathbf{p}}^{(\omega^{\pm})\updownarrow}_{\rho''_z}\right]^*\right).
\end{aligned}$$

Appendix VI.C: Calculation of Frobenius inner product associated to $\bar{\bar{\mathcal{G}}}^{(\omega^{\pm})}_{0,2}$

$$\begin{aligned}
\left\| \left[\hat{\bar{\bar{\mathbf{G}}}}^{[(\uparrow/\uparrow)]}_{z\leftarrow\rho_z}\right]^* \hat{\bar{\bar{\mathbf{G}}}}^{(\omega^{\pm})[(\uparrow/\uparrow)(\updownarrow/\updownarrow)(\updownarrow/\updownarrow)]}_{z\leftarrow\rho'_z\leftarrow\rho''_z\leftarrow\rho_z} \right\|^{(\mathrm{p})}_{\mathcal{F}} &\propto \left\| \hat{\mathbf{g}}^{(\mathrm{p})(\uparrow/\uparrow)*}_{z\leftarrow\rho_z} \hat{\mathbf{g}}^{(\mathrm{p})(\uparrow/\uparrow)}_{z\leftarrow\rho'_z} \hat{\mathbf{g}}^{(\mathrm{p})(\omega^{\pm})(\updownarrow/\updownarrow)}_{\rho'_z\leftarrow\rho''_z} \hat{\mathbf{g}}^{(\mathrm{p})(\updownarrow/\updownarrow)}_{\rho''_z\leftarrow\rho_z} \right\|_{\mathcal{F}} \\
&= \left\| \left(\hat{\mathbf{p}}^{\uparrow}_z \otimes \hat{\mathbf{p}}^{\uparrow}_{\rho_z}\right)^* \left(\hat{\mathbf{p}}^{\uparrow}_z \otimes \hat{\mathbf{p}}^{\uparrow}_{\rho'_z}\right) \left(\hat{\mathbf{p}}^{(\omega^{\pm})\updownarrow}_{\rho'_z} \otimes \hat{\mathbf{p}}^{(\omega^{\pm})\updownarrow}_{\rho''_z}\right) \left(\hat{\mathbf{p}}^{\updownarrow}_{\rho''_z} \otimes \hat{\mathbf{p}}^{\updownarrow}_{\rho_z}\right) \right\|_{\mathcal{F}} \\
&= \left(\hat{\mathbf{p}}^{\uparrow}_{\rho'_z} \cdot \hat{\mathbf{p}}^{(\omega^{\pm})\updownarrow}_{\rho'_z}\right) \left(\hat{\mathbf{p}}^{(\omega^{\pm})\updownarrow}_{\rho''_z} \cdot \hat{\mathbf{p}}^{\updownarrow}_{\rho''_z}\right) \left(\left[\hat{\mathbf{p}}^{\uparrow}_z\right]^* \cdot \hat{\mathbf{p}}^{\uparrow}_z\right) \left(\left[\hat{\mathbf{p}}^{\uparrow}_{\rho_z}\right]^* \cdot \hat{\mathbf{p}}^{\updownarrow}_{\rho_z}\right).
\end{aligned}$$

SUPPLEMENTARY FIGURES

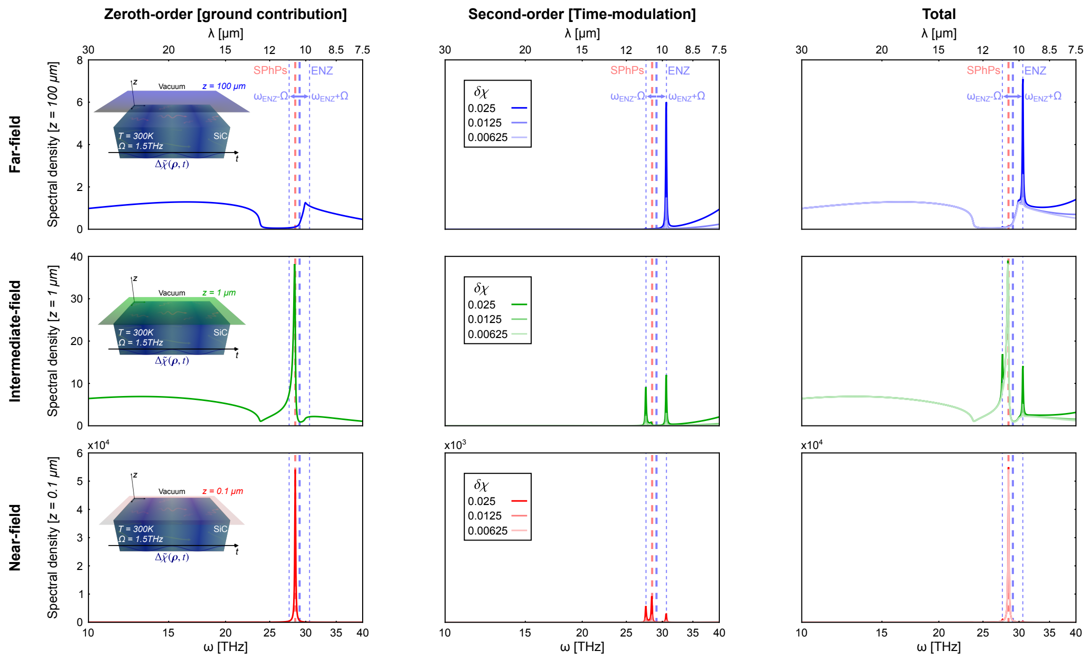

FIG. S1. **Dependence of the modulation strength, $\delta\chi$, on the emission spectra of a semi-infinite planar slab made of SiC with a time-varying susceptibility subjected to a time-harmonic modulation with $\Omega = 1.5$ THz.**

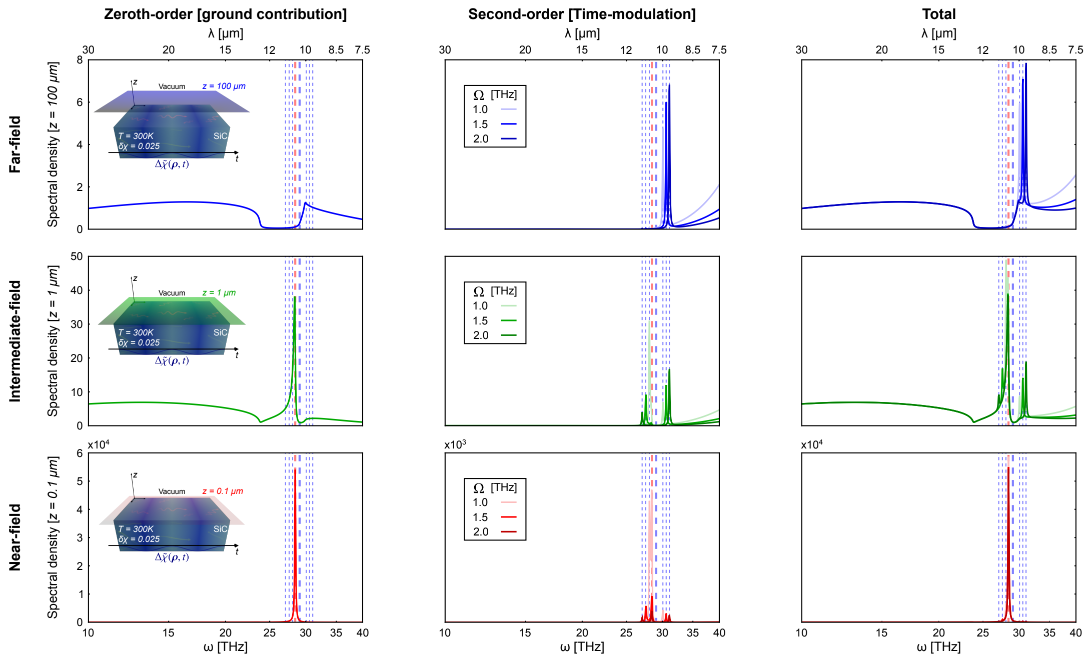

FIG. S2. **Dependence of the modulation frequency, Ω, on the emission spectra of a semi-infinite planar slab made of SiC with a time-varying susceptibility subjected to a time-harmonic modulation with $\delta\chi = 0.025$.**

* enrique.vazquez@unavarra.es
† inigo.liberal@unavarra.es

[1] Loudon, R. *The Quantum Theory of Light* (Oxford Univ. Press, 2000).

[2] Boyd, R. W. *Nonlinear Optics* (Academic Press, 2019).

[3] Vogel, W. & Welsch, D.-G. *Quantum Optics* (Wiley-VCH, 2006).

[4] Delga, A., Feist, J., Bravo-Abad, J. & García-Vidal, F. J. Quantum emitters near a metal nanoparticle: Strong coupling and quenching. *Phys. Rev. Lett.* **112,** 253601 (2014).

[5] Liberal, I., Ederra, I. & Ziolkowski, R. W. Control of a quantum emitter's bandwidth by managing its reactive power. *Phys. Rev. A* **100,** 023830 (2019).

[6] Scheel, S. & Buhmann, S. Y. Macroscopic quantum electrodynamics – Concepts and application. *Acta Phys. Slovaca* **58,** 675 (2008).

[7] Sloan, J., Rivera, N., Joannopoulos, J. D. & Soljačić, M. Casimir light in dispersive nanophotonics. *Phys. Rev. Lett.* **127,** 053603 (2021).

[8] Novotny, L. & Hecht, B. *Principles of Nano-Optics* (Cambridge University Press, 2012).

[9] Mandel, L. & Wolf, E. *Optical Coherence and Quantum Optics* (Cambridge Univ. Press, 1995).

[10] Joulain, K., Mulet, J.-P., Marquier, F., Carminati, R. & Greffet, J.-J. Surface electromagnetic waves thermally excited: Radiative heat transfer, coherence properties and Casimir forces revisited in the near field. *Surf. Sci. Rep.* **57,** 59 (2005).

[11] Sipe, J. E. New Green-function formalism for surface optics. *J. Opt. Soc. Am. B* **4,** 481 (1987).

[12] Carminati, R. & Greffet, J.-J. Near-field effects in spatial coherence of thermal sources. *Phys. Rev. Lett.* **82,** 1660 (1999).

[13] Shchegrov, A. V., Joulain, K., Carminati, R. & Greffet, J.-J. Near-field spectral effects due to electromagnetic surface excitations. *Phys. Rev. Lett.* **85,** 1548 (2000).
